# Ctf4 Links DNA Replication with Sister Chromatid Cohesion Establishment by Recruiting the Chl1 Helicase to the Replisome

**DOI:** 10.1016/j.molcel.2016.05.036

**Published:** 2016-08-04

**Authors:** Catarina P. Samora, Julie Saksouk, Panchali Goswami, Ben O. Wade, Martin R. Singleton, Paul A. Bates, Armelle Lengronne, Alessandro Costa, Frank Uhlmann

**Affiliations:** 1 Chromosome Segregation Laboratory, Francis Crick Institute, London WC2A 3LY, UK; 2 Institute of Human Genetics (IGH), CNRS, 34396 Montpellier, France; 3 Macromolecular Machines Laboratory, Francis Crick Institute, South Mimms EN6 3LD, UK; 4 Structural Biology of Chromosome Segregation Laboratory, Francis Crick Institute, London WC2A 3LY, UK; 5 Biomolecular Modelling Laboratory, Francis Crick Institute, London WC2A 3LY, UK

## Abstract

DNA replication during S phase is accompanied by establishment of sister chromatid cohesion to ensure faithful chromosome segregation. The Eco1 acetyltransferase, helped by factors including Ctf4 and Chl1, concomitantly acetylates the chromosomal cohesin complex to stabilize its cohesive links. Here we show that Ctf4 recruits the Chl1 helicase to the replisome via a conserved interaction motif that Chl1 shares with GINS and polymerase α. We visualize recruitment by EM analysis of a reconstituted Chl1-Ctf4-GINS assembly. The Chl1 helicase facilitates replication fork progression under conditions of nucleotide depletion, partly independently of Ctf4 interaction. Conversely, Ctf4 interaction, but not helicase activity, is required for Chl1’s role in sister chromatid cohesion. A physical interaction between Chl1 and the cohesin complex during S phase suggests that Chl1 contacts cohesin to facilitate its acetylation. Our results reveal how Ctf4 forms a replisomal interaction hub that coordinates replication fork progression and sister chromatid cohesion establishment.

## Highlights

• Ctf4 is a replisomal interaction hub that recruits CIP box client proteins
• Chl1 helicase is the Ctf4 client that links replication and cohesion establishment
• Ctf4 interaction, but not helicase activity, conveys Chl1 cohesion function
• Chl1 physically engages with cohesin during cohesion establishment

## Graphical Abstract

**Figure:**
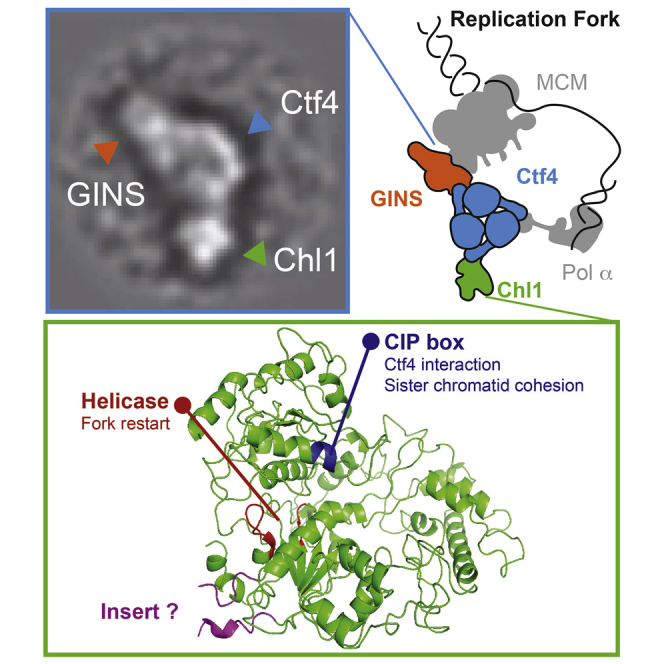

## Teaser

Eukaryotic DNA replication is accompanied by the establishment of sister chromatid cohesion. Samora et al. show that the central replisome component Ctf4 makes this link by recruiting the Chl1 helicase to the replication fork. Chl1 in turn engages in a direct physical contact with the cohesin complex during cohesion establishment.

## Introduction

Cohesion between sister chromatids, from the time of DNA replication in S phase until anaphase onset, is crucial for the faithful distribution of genetic information between daughter cells. Sister chromatid cohesion is mediated by the chromosomal cohesin complex, a large ring-shaped protein assembly that topologically encircles DNA (Nasmyth and Haering, 2009, Uhlmann, 2016). Cohesin is loaded onto chromosomes well before S phase, in late G1 phase in budding yeast and even earlier in fission yeast and human cells. Recent progress has been made in our understanding of how DNA enters the cohesin ring, facilitated by a separate cohesin loader complex (Murayama and Uhlmann, 2014). However, the association of cohesin with chromatin in itself is not sufficient to promote sister chromatid cohesion. The establishment of cohesive linkages between sister chromatids is an active process that occurs concomitantly with DNA replication and poses at least two requirements. First, cohesin must entrap not only one, but two strands of DNA. How this is achieved is not yet known. The replication fork might pass through cohesin rings, or cohesin might sequentially embrace two replicated DNAs in the wake of the replication fork, two possibilities that are not mutually exclusive. Second, cohesin rings that hold together sister chromatids must be stabilized on chromosomes, which in budding yeast is achieved through acetylation by the essential, replication fork-associated acetyltransferase Eco1 (Rolef Ben-Shahar et al., 2008, Ünal et al., 2008). Acetylation targets two DNA sensory lysines on the cohesin ATPase that fuel the DNA entry and exit reactions. Their acetylation switches off the dynamic loading and unloading cycle of the cohesin complex to establish enduring sister chromatid cohesion (Chan et al., 2012, Gerlich et al., 2006, Lopez-Serra et al., 2013, Murayama and Uhlmann, 2015). The importance of cohesin stabilization for cohesion establishment is illustrated by the fact that Eco1 becomes dispensable for viability if cohesin is stabilized on chromosomes in an alternative way. This can be achieved by deletion of the non-essential cohesin subunit Wapl, which promotes the dynamic turnover of the complex (Rolef Ben-Shahar et al., 2008).

In addition to the Eco1 acetyltransferase, a number of further “cohesion establishment factors” have been identified using genetic approaches in budding yeast. These are proteins that are not themselves part of the cohesin complex, but that contribute to the establishment of sister chromatid cohesion. Among them are the PCNA clamp loader and unloader RFC^Ctf18^ (Mayer et al., 2001); the three subunits Tof1, Csm3, and Mrc1 of the replication checkpoint complex (Mayer et al., 2004, Xu et al., 2004); the replisome component Ctf4 (Hanna et al., 2001); as well as the Chl1 helicase (Skibbens, 2004). Individually, these factors are not essential. However, inactivation of numerous pairwise combinations results in additive sister chromatid cohesion defects and lethality (Xu et al., 2007). Cohesin acetylation during S phase is reduced in the absence of any of these cohesion establishment factors, suggesting that they all act at least in part by facilitating the acetylation reaction (Borges et al., 2013). Genetic analysis is consistent with the possibility that RFC^Ctf18^, Tof1, Csm3, and Mrc1 act in a pathway with Eco1, as their deletion hardly increases the growth defect seen in a strain background lacking Eco1. In contrast, Ctf4 or Chl1 deletion causes a marked synthetic growth defect in the absence of Eco1 (Borges et al., 2013). This suggests that Ctf4 and Chl1 support cohesin acetylation by acting in parallel to Eco1. However, the molecular mechanism by which Ctf4 and Chl1 achieve this is not known.

Ctf4 was originally identified as a DNA polymerase α-interacting factor, important for chromosome stability. It was subsequently shown to be important for sister chromatid cohesion (Hanna et al., 2001, Kouprina et al., 1992, Miles and Formosa, 1992). We now know that Ctf4 is a structural component of the replisome, linking the MCM helicase via GINS to the DNA polymerase α-primase complex (Gambus et al., 2006, Gambus et al., 2009, Lengronne et al., 2006, Tanaka et al., 2009a). Recent structural work has shown that Ctf4 is a homotrimer to which GINS and DNA polymerase α bind via a shared interaction motif (Simon et al., 2014). Ctf4 and its role in sister chromatid cohesion are conserved in vertebrates (where Ctf4 is also known as And1; Errico et al., 2009).

Chl1 is encoded by what is probably the first chromosome loss mutant gene to be identified (Haber, 1974). Its cloning suggested that Chl1 is a DNA helicase (Gerring et al., 1990). Biochemical analysis confirmed that the human counterpart of Chl1 (known as ChlR1) is indeed a DNA helicase and that it progresses along single-stranded DNA in the 5′-3′ direction (Farina et al., 2008, Hirota and Lahti, 2000). Consistent with Chl1 function as a DNA helicase, its intact ATPase is required to prevent chromosome loss in both yeast and mice (L Holloway, 2000, Inoue et al., 2007). Chl1 promotes sister chromatid cohesion in yeast and humans (Farina et al., 2008, Mayer et al., 2004, Parish et al., 2006, Skibbens, 2004); however, a formal test of whether the Chl1 ATPase is required for sister chromatid cohesion, or in another way contributes to chromosome stability, is outstanding. Mutations in human ChlR1 are the cause of Warsaw breakage syndrome, a developmental disorder that combines features of defective DNA repair and cohesin function (van der Lelij et al., 2010).

Here, we show that Ctf4 and Chl1 physically interact. Chl1 is recruited to the budding yeast DNA replication fork via a conserved Ctf4-interaction peptide motif that it shares with polymerase α and GINS (which we suggest to be known as “CIP box”). Chl1 and Ctf4 form a multimer that is architecturally reminiscent of the polymerase α-Ctf4-GINS assembly, as observed by single-particle electron microscopy (EM) analysis of a reconstituted protein complex. Ctf4 interaction, but, surprisingly, not helicase activity, is required for Chl1 function in sister chromatid cohesion. This suggests a structural role for Chl1 in cohesion establishment that might involve a direct interaction with cohesin at replication forks. These findings show how Ctf4 forms an interaction hub within the replisome that links replication fork progression to sister chromatid cohesion establishment.

## Results

### Ctf4 and Chl1 Interact during DNA Replication

**Figure 1:**
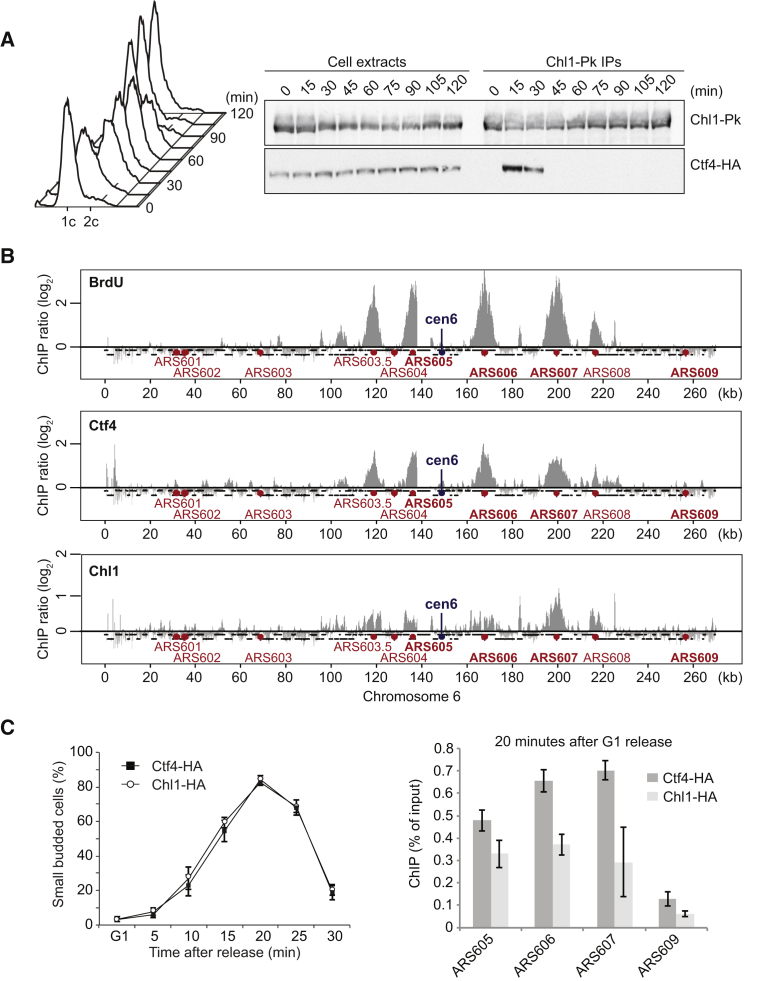
Ctf4 and Chl1 Interact and Co-localize during S Phase (A) Ctf4 and Chl1 interact during S phase. Cell extracts were prepared from aliquots of a culture passing through a synchronous cell cycle at the indicated times. Pk epitope-tagged Chl1 was immunoprecipitated, and co-precipitation of Ctf4 was analyzed by SDS-PAGE followed by immunoblotting. Cell-cycle progression was monitored by FACS analysis of DNA content. (B) Chl1 co-localizes with Ctf4 at HU-arrested replication forks. Cells were synchronized in G1 and released into BrdU- and HU-containing medium for arrest in early S phase. ChIP analysis against BrdU and epitope-tagged Ctf4 and Chl1 was performed. Chromatin immunoprecipitates were hybridized to Affymetrix GeneChip *S. cerevisiae* tiling 1.0 R arrays. Signal intensities, relative to a whole-genome DNA sample, are shown along chromosome 6. Replication origins are indicated; those chosen for subsequent quantitative analysis are highlighted in bold. The microarray data are available from the GEO database under the accession number GEO: GSE80007. (C) Chl1 localizes to replicating regions in early S phase under unchallenged conditions. Chl1 ChIP was performed 20 min after synchronous release from α factor block when cells reached early S phase, as seen by a large fraction of cells with small buds (less than half the diameter of the mother cell). Enrichment close to three early (ARS605, 606, and 607) and a late firing (ARS609) replication origin were compared. Ctf4 ChIP was performed for comparison. The means and SE of three independent experiments are shown. See also Figure S1 for an analysis of Ctf4 and Chl1 protein levels and subcellular localization during the cell cycle.

Previous studies have placed Ctf4 and Chl1 into one genetically defined cohesion establishment pathway. Deletion of the genes encoding either factor has the same impact on sister chromatid cohesion compared to deleting both (Borges et al., 2013, Xu et al., 2007). Physical complex formation is a familiar mechanism for two proteins to act in one pathway. Therefore, we asked whether Ctf4 and Chl1 interact. To test this, we immunoprecipitated Ctf4 from cells progressing synchronously through the cell cycle following α factor block and release. Chl1 co-precipitated with Ctf4 specifically during S phase, but not before or afterward (Figure 1A). This demonstrates that Ctf4 and Chl1 interact during the time of DNA replication.

To investigate whether Ctf4 interacts with Chl1 at the replication fork, we compared the chromosomal localization pattern of Ctf4 and Chl1 by chromatin immunoprecipitation (ChIP). Synchronized cells were arrested in early S phase by hydroxyurea (HU) treatment, and the position of active replication forks was determined by BrdU incorporation into newly synthesized DNA, followed by ChIP against BrdU (Figure 1B). ChIP against Ctf4 confirmed its localization to replication forks, as previously demonstrated (Gambus et al., 2006, Lengronne et al., 2006). When we performed ChIP against Chl1, we found that it was also enriched in replicating regions. This opens the possibility that Chl1 binds Ctf4 at DNA replication forks. Chl1 was detectable at active replication origins during early S phase in the absence of HU, suggesting that Chl1 associates with the replisome also during undisturbed S phase progression (Figure 1C).

The interaction between Ctf4 and Chl1 is restricted to S phase, so we asked whether cell-cycle regulation of either protein could explain this timing. However, levels and migration pattern during gel electrophoresis of both proteins remained constant throughout the cell cycle (Figures 1A and S1A, available online). We furthermore analyzed the subcellular localization of Ctf4 and Chl1 by indirect immunofluorescence. Ctf4 showed nuclear accumulation, while Chl1 displayed a diffuse staining pattern throughout the nucleus and cytoplasm, during all stages of the cell cycle (Figure S1B). Thus, neither protein is subjected to overt cell-cycle regulation. Instead, the interaction between Ctf4 and Chl1 might depend on replisome assembly, when concerted interactions between numerous proteins that are not individually detectable at other times (Gambus et al., 2009) come together to build the replication fork machinery.

### Chl1 Recruitment through a Conserved CIP Box Motif

**Figure 2:**
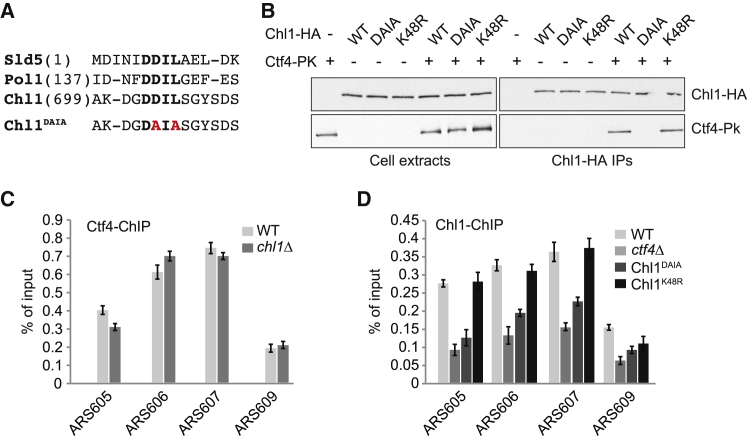
Ctf4 Recruits Chl1 to the Replisome via a Conserved CIP Box (A) Sequence alignment of the Ctf4-interacting peptide (CIP) boxes in *S. cerevisiae* Sld5 and Pol1 and a matching sequence in Chl1. Invariant residues are highlighted in bold. CIP box mutations to generate Chl1^DAIA^ are indicated in red. (B) CIP box-dependent interaction of Chl1 with Ctf4. Cell extracts were prepared from cultures of the indicated strains, synchronized, and arrested in early S phase by HU treatment. Chl1-HA was immunoprecipitated, and co-precipitation of Ctf4 detected by immunoblotting. (C) Ctf4 localizes to the replication fork independently of Chl1. Ctf4 ChIP was performed in synchronized cells arrested in early S phase by HU treatment in the presence or absence of Chl1. Enrichment at three early firing origins, active in HU (ARS605, 606, and 607), and a late-firing, inactive origin (ARS 609) was compared by quantitative real-time PCR. The means and SE of three independent experiments are shown. (D) Chl1 is recruited to replication forks by CIP box-dependent binding to Ctf4. As in (C), but Chl1 ChIP was performed in the indicated strains.

The GINS component Sld5 and the polymerase α large subunit Pol1 share a conserved Ctf4 binding motif that docks onto an exposed helical extension found on each Ctf4 protomer (Simon et al., 2014). By sequence gazing, we detected a match to this motif, “DDIL,” in Chl1 (Figure 2A). To test whether this motif mediates Chl1 interaction with Ctf4, we mutated two residues within this motif to alanines (Chl1^DAIA^). The crystal structure of the corresponding Sld5 peptide bound to Ctf4 shows these residues engaged in prominent contacts (Simon et al., 2014). We generated a budding yeast strain in which *CHL1* was replaced with *chl1*^*DAIA*^ at the endogenous gene locus, then visualized Chl1 interaction with Ctf4 using cells arrested in early S phase (Figure 2B). Ctf4 interaction was lost in the case of Chl1^DAIA^, even though Chl1^DAIA^ was stably expressed at levels equal to wild-type Chl1. As an additional control, a Chl1 variant carrying a mutation in the helicase active site (Chl1^K48R^) was included in the analysis, which retained association with Ctf4. This suggests that a conserved CIP box mediates the interaction of Chl1 with Ctf4.

We next studied the importance of the Ctf4-Chl1 interaction for the recruitment of both proteins to the replication fork. We used ChIP followed by quantitative real-time PCR (ChIP quantitative real-time PCR) from cells arrested in early S phase by HU treatment to assess protein binding near three early firing, active replication origins. A region close to a late-firing origin, inactive in HU-treated cells, served as a control. Ctf4 was readily detected at the three active origins, and the level of association was unaltered in cells lacking Chl1 (Figure 2C). Thus, Ctf4 association with the replisome is independent of Chl1. Conversely, Chl1 binding to the same origins was greatly reduced in the absence of Ctf4, suggesting that Ctf4 recruits Chl1 (Figure 2D). Chl1^DAIA^ binding to origins, in the presence of Ctf4, was reduced compared to wild-type Chl1, demonstrating a role of the CIP box in recruiting Chl1 to the replisome. A lower level of Chl1^DAIA^, which remained detectable, suggests that Chl1 makes additional contacts with Ctf4 or other components of the replisome. Chl1 helicase activity was not required for recruitment to the replisome, as Chl1^K48R^ was detected at levels equal to the wild-type protein.

### Visualization of the Ctf4-Chl1 Interaction

**Figure 3:**
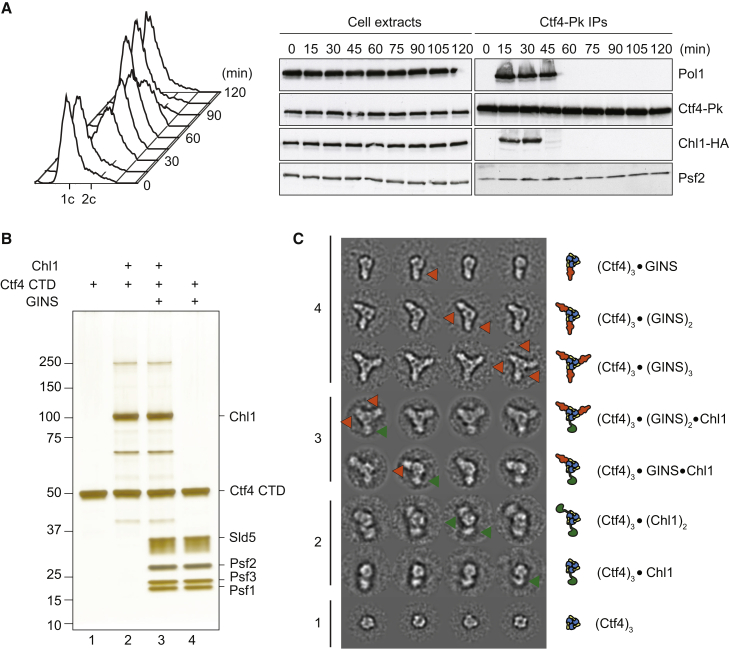
Ctf4 Is a Recruitment Platform at the Replication Fork (A) Cell-cycle pattern of Ctf4 interactions. Cell extracts were prepared at the indicated times from a culture progressing synchronously through the cell cycle. Ctf4 was immunoprecipitated, and co-precipitation of the GINS subunit Psf2, the polymerase α subunit Pol1, and Chl1 was analyzed by immunoblotting. Cell-cycle progression was monitored by FACS analysis of DNA content. (B) Silver-stained gel of the reconstituted Ctf4 client protein assemblies. Ctf4 was prepared in isolation or bound by Chl1, GINS, or both. (C) Two-dimensional averages of Chl1-client protein assemblies imaged by negative-stain EM. Chl1 is highlighted by green arrowheads and in the same color in the diagrams. GINS is orange and Ctf4 is blue/yellow. Box size is 441 Å × 441 Å. See also Figure S2 for more reference-free class averages of the protein assemblies.

Ctf4 forms a homotrimer within the replisome, offering three docking sites to client proteins (Simon et al., 2014). Two of these sites are used to bridge GINS and the polymerase α-primase complex, opening the possibility that the third protomer simultaneously recruits Chl1. To obtain insight into the temporal regulation of Ctf4 interaction with three binding partners, we again immunoprecipitated Ctf4 at intervals from cells that progressed synchronously through the cell cycle and probed for co-precipitation of the three interactors. This analysis revealed a constitutive interaction of Ctf4 with the GINS subunit Psf2, detectable throughout the cell cycle, consistent with a previous report (Gambus et al., 2009; Figure 3A). Chl1 binding became detectable at the onset of S phase, also when the polymerase α subunit Pol1 associated with Ctf4. Chl1 association was lost 30 min later, when most DNA replication was complete. In contrast, Pol1 remained detectable with Ctf4 for another 15 min, before its association was also lost. These findings suggest that Ctf4 interacts stably with GINS, while its interactions with Chl1 and the polymerase α-primase complex occur with overlapping yet distinct temporal regulation.

To provide visual evidence that a replisome-incorporated Ctf4 trimer can associate with Chl1, we recombinantly expressed and purified Chl1, the hetero-tetrameric GINS complex, and the Ctf4 C-terminal trimerization core that contains the CIP box acceptor. Following an established protocol (Simon et al., 2014), we reconstituted various permutations of Ctf4-client protein assemblies for characterization by two-dimensional single-particle EM (Figure 3B). As previously reported, the Ctf4 core forms a trimeric disk. We confirmed that, mixed in equimolar amounts, one, two, or three GINS assemblies bind to one Ctf4 complex and form a rod-like feature that radially departs from the homo-trimerization core. Performing the same reconstitution experiment with Ctf4 and Chl1 yields a partially occupied Ctf4 trimer, with one or two (but not three) hook-shaped Chl1 molecules binding to the Ctf4 disk (Figures 3C and S2A). This is compatible with the notion that Chl1 is a transient Ctf4 interactor in vivo, compared to constitutively bound GINS. A reconstituted Ctf4 hetero-complex containing both client proteins reveals a Ctf4 assembly concurrently associated with both rod-shaped GINS and hook-shaped Chl1 molecules (Figures 3C and S2B). This shows that Ctf4 can physically bridge a replisome component with the Chl1 helicase.

### The Ctf4-Chl1 Interaction Promotes Sister Chromatid Cohesion

**Figure 4:**
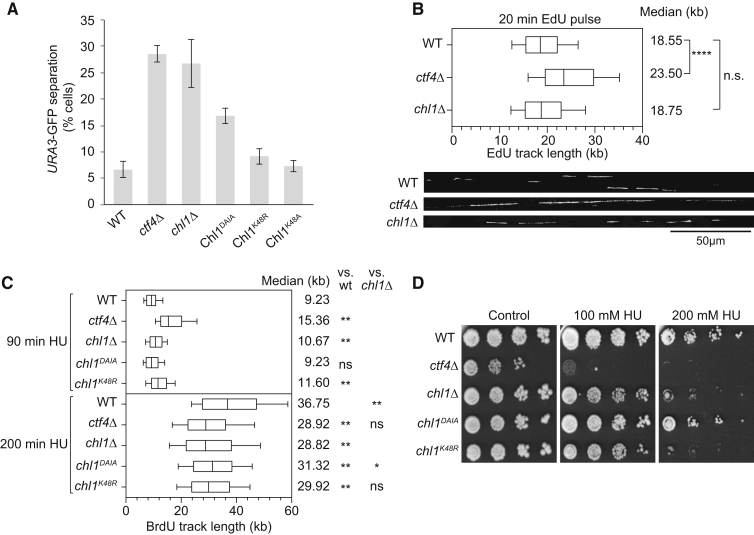
Separable Chl1 Functions in Sister Chromatid Cohesion and Replication Fork Integrity (A) Ctf4 interaction, but not Chl1 helicase activity, contributes to sister chromatid cohesion. Cells of the indicated genotypes were synchronized and arrested in a nocodazole-imposed mitotic arrest. Sister chromatid cohesion was assessed at the GFP-marked *URA3* locus. The means and SE ofthree independent experiments are shown. See also Figure S3, showing that Chl1 overexpression cannot provide Chl1 function without Ctf4 and that ATPase activity is abrogated in recombinant Chl1^K48R^ and Chl1^K48A^ proteins. (B) Chl1 makes no detectable contribution to replication fork progression under unchallenged conditions. Fork speed, measured after pulse incorporation of 5-ethynyl-2**’**-deoxyuridine (EdU) and DNA combing, was analyzed in the indicated strains. Examples of the EdU tracks in each of the strains are shown. The graph depicts the distribution of EdU track lengths. Box and whiskers indicate 25–75 and 10–90 percentiles, respectively. Medians are shown by a line and are listed. Asterisks indicate the significance of the statistical test (^∗∗^p < 0.0001, ^∗^p = 0.0021; n.s., not significant; Mann-Whitney unpaired non-parametric t test). (C) Chl1 and Ctf4 are required for replication fork progression under conditions of dNTP depletion. As in (B), but cells of the indicated genotypes were synchronized by α factor block and release into medium containing BrdU and 200 mM HU. BrdU track lengths were measured at 90 and 200 min after release. (D) Chl1 helicase activity is required for HU-resistant cell growth. Ten-fold serial dilutions of strains of the indicated genotypes were spotted on YPD agar containing the indicated HU concentrations.

We next addressed whether Chl1 interacts with Ctf4 to establish sister chromatid cohesion. For this, we took advantage of the Chl1^DAIA^ mutant, which is expressed at levels equal to wild-type Chl1, but whose Ctf4 interaction and association with replication forks are compromised. To assess sister chromatid cohesion, we monitored the GFP-marked *URA3* locus. Cells were synchronized by α factor block and release and were then arrested in mitosis by nocodazole treatment. *ctf4Δ* and *chl1Δ* strains were included in this experiment, which displayed expected cohesion defects (Hanna et al., 2001, Skibbens, 2004) (Figure 4A). The *chl1*^*DAIA*^ mutant cells also showed a marked cohesion defect, albeit not to the full extent seen in *ctf4Δ* or *chl1Δ* cells. This demonstrates the importance of the Ctf4-Chl1 interaction for sister chromatid cohesion. Residual Chl1^DAIA^ association with the replisome might account for the less severe phenotype. The importance of Ctf4 interaction for Chl1 function was underscored in an experiment in which we overexpressed Chl1 with the aim of providing Chl1 function independently of Ctf4. Expression under control of the galactose-inducible *GAL1* promoter led to greatly increased Chl1 levels. This fully restored sister chromatid cohesion in a *chl1Δ* background, but not in the absence of Ctf4 (Figure S3A). Thus, the ability to interact with Ctf4 is crucial for Chl1’s function in sister chromatid cohesion.

Chl1 is thought to function as a helicase in chromosome stability (L Holloway, 2000, Inoue et al., 2007). We therefore asked whether helicase function is required for sister chromatid cohesion. Previous studies have replaced a conserved lysine in the ATP binding site with arginine, which in the case of the human ChlR1 enzyme abolishes helicase activity in vitro (Farina et al., 2008, Hirota and Lahti, 2000). To our surprise, the Chl1^K48R^ mutation in budding yeast Chl1 did not compromise sister chromatid cohesion (Figure 4A). Chl1^K48R^ might have retained residual helicase function in vivo, so we introduced a more severe K48A mutation. Cells expressing Chl1^K48A^ as the sole source of Chl1 again did not display a noticeable sister chromatid cohesion defect. To confirm that the mutant Chl1 proteins were deficient in helicase activity, we purified budding yeast Chl1, Chl1^K48R^, and Chl1^K48A^ following overexpression in insect cells (Figures S3B and S3C). Biochemical analysis confirmed that both the arginine and alanine substitutions obliterated the ability of Chl1 to hydrolyze ATP in the presence of single-stranded DNA. These results confirm that Chl1^K48R^ and Chl1^K48A^ are helicase deficient and suggest that helicase activity is not required for Chl1 to fulfill its role in sister chromatid cohesion.

### Separable Chl1 Functions in Sister Chromatid Cohesion and Replication Fork Progression

Surprised by the finding that Chl1 helicase activity is dispensable for sister chromatid cohesion, we investigated whether the Chl1 helicase contributes to replication fork progression. The main replicative MCM helicase moves in 3′-5′ direction along the leading strand. As a 5′-3′ helicase, Chl1 could act in parallel, promoting DNA unwinding on the lagging strand. To investigate whether Chl1 contributes to DNA replication, we monitored replication fork progression in an unchallenged, exponentially growing cell population during a 20 min pulse with the thymidine analog 5-ethynyl-2′-deoxyuridine (EdU). EdU tracks were then visualized along individual DNA fibers that were stretched by DNA combing, and the track lengths were measured. There was no significant difference in fork progression rates between *chl1Δ* and wild-type cells (Figure 4B). Therefore, Chl1 does not make a detectable contribution to DNA replication under unchallenged conditions. Consistent with previous observations, replication forks were significantly accelerated in *ctf4Δ* cells, probably due to the increased deoxynucleotide (dNTP) pools in these cells (Poli et al., 2012).

We next analyzed fork progression following release from α factor block into medium containing HU, which slows down replication fork progression due to dNTP depletion, and BrdU. At early times after release (Figure 4C; 90 min), while existing nucleotide pools are used up, track lengths showed a similar pattern to what was observed in unchallenged cells. This situation changed after longer times in the arrest (200 min). Wild-type replication forks continued to progress slowly. Notably, forks progressed even slower in *ctf4Δ*, *chl1Δ*, and *chl1*^*K48R*^ cells, and also *chl1*^*DAIA*^ cells, albeit to a lesser degree in the latter. This suggests that both Ctf4 and Chl1 are required to maintain fork progression under conditions of replication stress. The Chl1 helicase might do so by aiding replication fork restart following repeated rounds of stalling due to the low dNTP levels. Observations that support these conclusions have recently been made in human cells lacking ChlR1 (Calì et al., 2016).

Consistent with a role of Ctf4 and Chl1 in response to replication stress, strains lacking these proteins showed growth retardation on HU-containing medium. This was very pronounced in the case of *ctf4Δ* cells, while growth of *chl1Δ* cells was compromised less severely (Figure 4D). This suggests that Ctf4 plays a role in response to HU in addition to recruiting Chl1. This might include its interaction with the polymerase α-primase or other factors, e.g., Mms22 (Gambus et al., 2009, Mimura et al., 2010). Chl1 function in response to HU depended less on its interaction with Ctf4, as Chl1^DAIA^ conferred HU resistance equal to wild-type Chl1. Helicase activity in turn was required, as a *chl1*^*K48R*^ strain was equally HU sensitive as a *chl1Δ* strain. Taken together, this ascribes two separable functions to Chl1. Its helicase facilitates DNA replication under conditions of dNTP depletion. Cohesion establishment, in contrast, requires that Chl1 binds to Ctf4, but does not involve its helicase activity.

### A Chl1-Specific Insertion in the XPD Helicase Family

**Figure 5:**
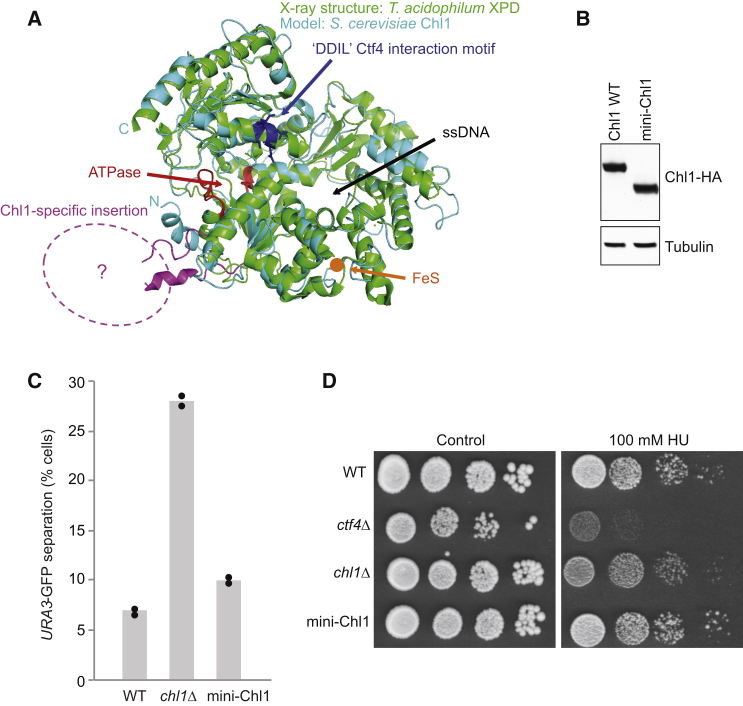
A Chl1-Specific Insert in XPD Family Helicases (A) A structural model of *S. cerevisiae* Chl1 based on the *T. acidophilum* XPD helicase crystal structure (Wolski et al., 2008). Regions containing the ATPase motifs are highlighted in red, the position of the 4Fe4S cluster is indicated in orange, while the CIP box sequence is shown in dark blue. The position of the Chl1-specific insertion is indicated in pink. See also Figure S4 for a sequence alignment of Chl1 and XPD, showing the Chl1-specific insert and how it was removed and the gap sealed to create mini-Chl1. (B) Expression of mini-Chl1, lacking the Chl1-specific insertion. Whole-cell extracts, prepared from asynchronous cultures, were separated by SDS-PAGE. Chl1 and mini-Chl1 were detected by immunoblotting against their C-terminal HA epitope tags. Tubulin served as a loading control. (C) mini-Chl1 promotes sister chromatid cohesion. Sister chromatid cohesion in the indicated strains was analyzed as in Figure 4A. The results and means from two independent experiments are shown. (D) mini-Chl1 promotes HU-resistant cell growth. Ten-fold serial dilutions of the indicated strains were spotted on YPD agar without or containing 100 mM HU.

If not as a helicase, how does Chl1 facilitate cohesion establishment? Chl1 is a member of the XPD family of DNA helicases with roles in genome stability, characterized by a distinctive iron sulfur cluster (White, 2009). Chl1 is singled out within the family by an ∼20 kDa domain insertion between the Walker A and B motifs of the ATPase active site (Figure S4). We therefore wondered whether this Chl1-specific insert mediates Chl1’s role in sister chromatid cohesion. To address this, we constructed a variant Chl1 lacking this insertion. An internal deletion of 179 amino acids was designed, guided by a structural alignment of budding yeast Chl1 with the crystal structure of *T. acidophilum* XPD (Wolski et al., 2008). A 12-amino-acid peptide linker to seal the deletion was created based on the XPD sequence (Figures 5A and S4). The resultant “*mini-CHL1*” gene was used to replace endogenous budding yeast *CHL1*. Mini-Chl1 was expressed as a stable protein at levels comparable to full-length Chl1 (Figure 5B).

We now tested the ability of mini-Chl1 to support the establishment of sister chromatid cohesion. Cells expressing mini-Chl1 showed a small increase of cells with premature sister chromatid separation compared to wild-type cells, but the defect was far less than that in *chl1Δ* cells (Figure 5C). This suggests that the Chl1-specific insert makes only a small contribution to sister chromatid cohesion. The main contribution to sister chromatid cohesion must be made by another part of Chl1. We also found that mini-Chl1 confers HU resistance to a degree indistinguishable from wild-type Chl1 (Figure 5D). Thus, a role for the Chl1-specific insert remains to be assigned in future studies.

### Chl1 and Chromosomal Cohesin Levels

Previous studies reported that chromosomal cohesin levels are reduced to about half in cells lacking Chl1 (Borges et al., 2013, Laha et al., 2011, Rudra and Skibbens, 2013). This reduction by itself is unlikely to explain the cohesion defect in *chl1Δ* cells, as even greater reduction of cohesin levels is inconsequential in the presence of Chl1 (Heidinger-Pauli et al., 2010). However, to know the reason for reduced cohesin levels might help to understand how Chl1 works.

**Figure 6:**
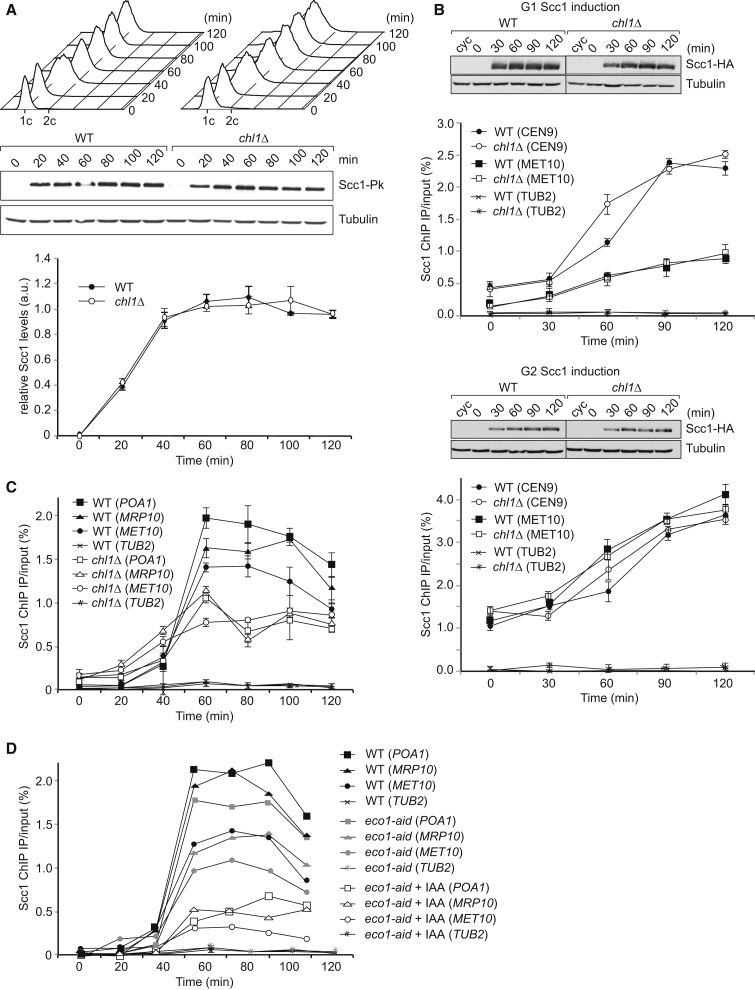
Acetylation Augments Chromosomal Cohesin Levels (A) Cohesin levels in the absence of Chl1. Scc1 levels were analyzed by quantitative immunoblotting in cultures passing synchronously from α factor block into nocodazole-imposed mitotic arrest. α-tubulin served as loading control for normalization. Cell-cycle progression was monitored by FACS analysis of DNA content. Means and SD from three independent experiments are shown. See also Figure S5 for a control that integrity of the cohesin complex is unaffected in cells lacking Chl1, and Figure S6A that Scc1 levels are unaffected in the absence of Ctf4. (B) Ectopic cohesin loading onto chromosomes is unaffected by Chl1. Scc1 was expressed under control of the *GAL1* promoter in G1 or in mitotically arrested cells. Ectopic Scc1 levels were monitored by immunoblotting, and its loading onto chromosomes was quantified by ChIP quantitative real-time PCR at a centromeric (*CEN9*) and a chromosome arm (*MET10*) cohesin-binding site; a negative site (*TUB2*) was included as control. All immunoblot samples were run on the same gel, separated by additional lanes where indicated. See also Figure S5B for confirmation of cell synchrony by FACS analysis of DNA content and Figure S6B, showing that cohesin loading is unaffected in the absence of Ctf4. (C) Chl1 augments chromosomal cohesin levels during S phase. Cohesin association with three chromosome arm regions (*POA1*, *MRP10*, and *MET10*) and a negative control site (*TUB2*) was quantified in wild-type and *chl1Δ* cells passing synchronously from α factor block into nocodazole-imposed mitotic arrest. Means and SD from three independent experiments are shown. See also Figure S5C for confirmation of cell synchrony by FACS analysis of DNA content and Figure S6C, showing that Ctf4 is required for increased cohesin binding to chromosome during S phase. (D) Cohesin acetylation promotes increased cohesin association with chromosomes during S phase. As in (C), but chromosomal cohesin levels were compared between wild-type and *MET3-eco1-aid* cells that were synchronized by α factor addition under permissive (−methionine) or restrictive (+ methionine, + auxin indole acetic acid [IAA]) conditions. See also Figure S5D for confirmation of cell synchrony by FACS analysis of DNA content.

We first established whether total cellular cohesin levels were altered in the absence of Chl1, which could explain reduced chromosomal levels. Quantitative immunoblotting from cultures progressing synchronously through the cell cycle revealed similar Scc1 subunit levels between wild-type and *chl1Δ* cells (Figure 6A). Scc1 was used as a representative cohesin subunit, as its stability depends on Smc1 and Smc3 (Tóth et al., 1999). Furthermore, immunoprecipitation of Scc1 from wild-type and *chl1Δ* cells confirmed equal abundance of the cohesin complex (Figure S5A). Therefore, reduced cohesin levels on chromosomes in *chl1Δ* cells are unlikely due to reduced availability of the cohesin complex.

We next asked whether cohesin loading onto chromosomes is compromised in the absence of Chl1. We arrested cells in either G1 phase or mitosis by α factor or nocodazole treatment, respectively. Once arrested, ectopic expression of Scc1 was induced under control of the galactose-inducible *GAL1* promoter. In G1, separase-resistant Scc1R180,268E was expressed to prevent cleavage by separase that is active at this time (Uhlmann et al., 1999). We then compared accumulation of Scc1 in whole-cell extracts by immunoblotting and on chromosomes by ChIP (Figure 6B). Scc1 accumulated with equal kinetics in wild-type and *chl1Δ* cells, first in cell extracts and then on chromosomes. Thus, Chl1 made no measurable contribution to ectopic cohesin loading. The same was observed in cells lacking Ctf4 (Figures S6A and S6B).

It has been suggested that Chl1 promotes cohesin loading specifically during S phase (Rudra and Skibbens, 2013), an effect that might have been missed in the above experiment. We therefore analyzed chromosomal cohesin levels as wild-type and *chl1Δ* cells passed synchronously through the cell cycle. Chromosomal cohesin initially accumulated at comparable rates in both *chl1Δ* and wild-type cells. Around the time of S phase, the cohesin ChIP signal in wild-type cells displayed a sharp increase. This increase depended on Chl1 and was not observed in its absence (Figure 6C). Similarly, it depended on Ctf4 (Figure S6C). Because cohesin acetylation occurs around this time, and because cohesin acetylation is compromised in the absence of Chl1 (Borges et al., 2013), we investigated whether acetylation was responsible for increased chromosomal cohesin. We performed a similar ChIP time course experiment following auxin-induced degradation of the cohesin acetyltransferase Eco1 (Figure 6D). This analysis revealed that acetylation is required to augment chromosomal cohesin levels during S phase. This can be rationalized if we consider that acetylation stabilizes cohesin on chromosomes (Chan et al., 2012, Lopez-Serra et al., 2013), which is expected to shift the equilibrium between free and chromosomal cohesin toward the chromosome-bound state. Biochemical fractionation similarly indicated that Eco1 augments chromosomal cohesin levels (Tóth et al., 1999). This opens the possibility that Chl1 impacts chromosomal cohesin levels by facilitating cohesin acetylation.

### Chl1 Promotes Efficient Cohesin Usage during Cohesion Establishment

**Figure 7:**
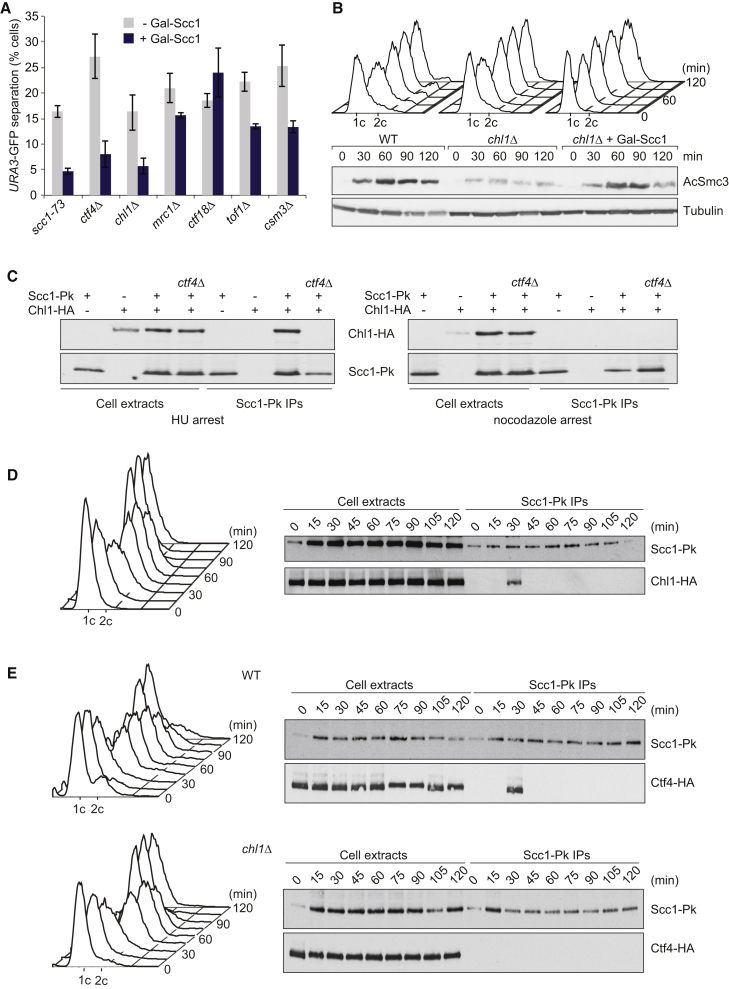
Chl1 Engages Cohesin during S Phase (A) Scc1 overexpression rescues sister chromatid cohesion in cells lacking Chl1 or Ctf4. Sister chromatid cohesion was assessed in the indicated strains as in Figure 4A. The strains were grown in the absence or presence of galactose to induce ectopic Scc1 expression under control of the *GAL1* promoter. Means and SE of three independent experiments are shown. (B) The Smc3 acetylation defect of *chl1Δ* cells is corrected by Scc1 overexpression. Cells of the indicated genotypes were synchronized in G1 and released into nocodazole-imposed mitotic arrest. The Smc3 acetylation status was analyzed by immunoblotting using an acetyl-Smc3-specific antibody. Tubulin served as loading control. FACS analysis of DNA content was used to monitor cell-cycle progression. (C) Chl1 interacts with cohesin at the replication fork. Pk epitope-tagged Scc1 was immunoprecipitated, and co-precipitation of Chl1 was analyzed by immunoblotting. Whole-cell extracts and immunoprecipitates of strains of the indicated genotype were prepared from cultures synchronized in early S phase (HU arrest) or mitosis (nocodazole arrest). See also Figure S7A for a control that the Chl1-cohesin interaction is CIP box dependent and unaltered by benzonase treatment. (D) Chl1 interacts with cohesin during S phase. As in (C), but Scc1 was immunoprecipitated from extracts made from aliquots of a culture synchronized by α factor block and release. (E) Cohesin interaction with the replisome is mediated by Chl1. As in (D), but interaction of Scc1 with Ctf4 was analyzed in strains with or without Chl1. See also Figure S7B for a ChIP experiment showing that Chl1 is enriched in replicating regions, but not cohesin-binding sites, and Figure S7C, which shows that Chl1 localizes to the replisome independently of cohesion.

To address how Chl1 promotes cohesin acetylation, we asked whether sister chromatid cohesion in *chl1Δ* cells could be improved by increased cohesin levels. Scc1 overexpression almost completely rescued sister chromatid cohesion in *chl1Δ*, as well as in *ctf4Δ* cells (Figure 7A). Similar cohesin overexpression did not rescue (or only partially rescued) the cohesion defects seen in other cohesion establishment mutants, *ctf18Δ*, *mrc1Δ*, *tof1Δ*, and *csm3Δ*. Thus, a bigger cohesin pool increases the chances of successful cohesion establishment in the absence of Ctf4 or Chl1, but not, or less so, in the absence of other cohesion establishment factors.

As another readout for cohesion establishment, we analyzed cohesin acetylation, which is greatly reduced in *chl1Δ* cells. Scc1 overexpression rescued acetylation levels to close to those seen in wild-type cells (Figure 7B). This suggests that cohesin is inefficiently used as an acetylation substrate in the absence of Chl1. In other words, Chl1 promotes the efficient use of cohesin for acetylation and thus cohesion establishment.

### Chl1 Engages with Cohesin during S Phase

It has been reported that human ChlR1 and cohesin co-precipitate from crude cell extracts (Parish et al., 2006, Yoshizawa-Sugata and Masai, 2009). We therefore investigated this interaction. Co-immunoprecipitation experiments revealed that the two proteins interacted in a cell-cycle-dependent manner (Figure 7C). The interaction was detected in cells synchronized in S phase by HU treatment, but not if cells were arrested in mitosis using nocodazole. Furthermore, Chl1 must be part of the replication fork to interact with cohesin, as no interaction was observed in the absence of Ctf4 (Figure 7C), or in case of Chl1^DAIA^ (Figure S7A). The cohesin-Chl1 interaction was resistant to benzonase treatment and is therefore likely protein rather than DNA mediated. The interaction was also detected in cells passing synchronously through the cell cycle during a short period in S phase (Figure 7D). Additional evidence that the cohesin-Chl1 interaction is restricted to the replisome came from ChIP analyses. Chl1 localizes to sites of DNA replication, but did not become enriched at cohesin-binding sites during or after S phase (Figure S7B). Furthermore, Chl1 associates with the replisome independently of cohesin (Figure S7C). Thus, replisome-bound Chl1 transiently engages with cohesin, probably as the replication fork moves along chromosomes and encounters cohesin-binding sites.

A cohesin-Chl1 interaction at the replication fork could reflect a direct cohesin-Chl1 contact, or it could be mediated by another replisome component. To differentiate between these possibilities, we analyzed the interaction of cohesin with Ctf4, which could also be detected during S phase (Figure 7E). The cohesin-Ctf4 interaction was lost in the absence of Chl1, suggesting that the replisome contacts cohesin via Chl1. Taken together, these results are consistent with a scenario in which Chl1 engages with cohesin as the replisome passes cohesin-binding sites, which facilitates retention and acetylation of cohesin during fork passage.

## Discussion

In this study, we investigated the contributions of Ctf4 and Chl1 to cohesion establishment and found that Ctf4 recruits Chl1 to the replication fork. Removing Ctf4 from cells that lack Chl1 does not increase their cohesion defect, suggesting that Ctf4’s role in sister chromatid cohesion is explained by its role in recruiting Chl1.

Ctf4 forms a homotrimer. Two of the protomers link the replicative helicase via GINS to the DNA polymerase α-primase complex (Simon et al., 2014). This left open whether the third protomer binds a second copy of polymerase α-primase, in analogy to stoichiometries observed at a prokaryotic replication fork (Reyes-Lamothe et al., 2010), or recruits additional unknown factor(s) to the replisome. We found that the latter is the case; Ctf4 recruits Chl1 via a “CIP box” that is shared between the three Ctf4 ligands. Despite these similarities, their respective interactions display individual characteristics. GINS appears to interact with Ctf4 most stably, retaining demonstrable contact throughout the cell cycle. Interactions with polymerase α and Chl1 are detectable only during S phase. Furthermore, Chl1 loses its interaction with Ctf4 earlier than polymerase α. The basis for this distinct temporal regulation remains to be explored. Additional contacts with replisome components might fine-tune the interaction dynamics. The parallel discovery of further CIP box clients that interact with Ctf4 during S phase (Villa et al., 2016) opens intriguing questions as to how their access to Ctf4 is regulated and how handover between CIP box proteins is coordinated.

Ctf4 has also been implicated in other chromosomal functions, including DNA repair and recovery from replication stress (Mimura et al., 2010, Poli et al., 2012, Tsutsui et al., 2005). Reported interactions of vertebrate Ctf4 with replication checkpoint proteins and the Dna2 helicase might contribute to these functions (Errico et al., 2009, Tanaka et al., 2009b, Wawrousek et al., 2010). Fission yeast Ctf4 in turn promotes centromere integrity by associating with the F-box protein Pof3 (Mamnun et al., 2006). One Ctf4 interactor, Mms22, is known to interact with the N-terminal half of Ctf4 (Gambus et al., 2009, Mimura et al., 2010). This part of the protein is predicted to form a WD40 repeat interaction surface, in addition to the C-terminal WD40 repeats, whose helical extensions form the CIP box acceptor. Ctf4 thus appears to be a multifaceted interaction platform that offers more than one possibility to add functionalities to the replisome.

Ctf4’s interaction with its various CIP box clients is reminiscent of PCNA, the DNA sliding clamp that recruits numerous targets to the replication fork via their shared PIP box motif (Georgescu et al., 2015). In addition to its essential role in supporting lagging-strand DNA synthesis by polymerase δ, PCNA engages with proteins involved in Okazaki fragment maturation, base excision repair, and chromatin assembly, among others. While Ctf4 takes on a central position within the replisome, PCNA is thought to remain DNA bound for a certain period following completion of DNA synthesis. In this way, protein interaction platforms exist at two locations, one close to the point of DNA unwinding and one in the wake of the fork. When it comes to sister chromatid cohesion establishment, both of these platforms appear to be used. Chl1 is recruited by Ctf4 to fulfill its function, while Eco1 contains an essential PIP box motif (Moldovan et al., 2006), whose contribution to cohesion establishment remains to be fully understood.

Chl1 is a member of the XPD family of helicases with varied functions in genome stability. Its founding member XPD unwinds DNA during nucleotide excision repair. The family also includes FANCJ and RTEL, with roles in interstrand crosslink repair and telomere maintenance (White, 2009). The Chl1 helicase acts in the recovery from replication stress (Calì et al., 2016, Laha et al., 2011), a function that we find is separable from its role in sister chromatid cohesion. Instead, we describe a physical interaction of Chl1 with the cohesin complex at the replication fork. This finding resolves the conundrum of how Ctf4 and Chl1 facilitate cohesin acetylation, yet act in a genetic pathway in parallel to Eco1 (Borges et al., 2013). Chl1 appears to act at the substrate level, e.g., by orienting the cohesin complex in a way that facilitates Smc3 acetylation. The fact that Ctf4 and Chl1 contribute to sister chromatid cohesion even in the absence of Eco1 suggests that the cohesin-Chl1 interaction facilitates cohesion establishment also independently of acetylation, e.g., by preventing cohesin loss from DNA during fork passage, a possibility that merits further investigation.

Our description of two separable roles of the Chl1 helicase might well be applicable to human ChlR1, which has been implicated both in replication fork restart and sister chromatid cohesion (Calì et al., 2016, Farina et al., 2008). It will be interesting to distinguish whether loss of Chl1 helicase function, or loss of a structural role as cohesin interactor, is the cause of Warsaw breakage syndrome. The observation that the syndrome combines characteristics of DNA repair defects and cohesin deficiency could be explained if both functions contribute.

## Experimental Procedures

Yeast culture and the yeast molecular biology techniques, including chromatin immunoprecipitation and replication fork speed measurements, followed standard or otherwise published procedures. These are detailed in the Supplemental Experimental Procedures. For biochemistry and EM analyses, Ctf4 (residues 471–927) and the GINS complex were purified following overexpression in *E. coli*, while Chl1 was purified following overexpression in insect cells. Details of the purification protocols and of the EM and single-particle analysis can be found in the Supplemental Experimental Procedures. An explanation of the structural model of Chl1 and the design of mini-Chl1 is also included there.

## Author Contributions

C.P.S and F.U. conceived the study, C.P.S. performed most experiments, J.S. and A.L. performed the replication fork speed analyses, P.G. and A.C. reconstituted the recombinant GINS-Ctf4-Chl1 assembly and performed its EM structural analysis, B.O.W. and M.R.S. purified Chl1 and performed ATPase assays, P.A.B. made the Chl1 structural model to design mini-Chl1, and C.P.S. and F.U. wrote the manuscript with input from all authors.

## Supplemental Information

Document S1. Supplemental Experimental Procedures, Figures S1–S7, and Table S1

Document S2. Article plus Supplemental Information
