# Supplementary material for: Ctf4 Links DNA Replication with Sister Chromatid Cohesion Establishment by Recruiting the Chl1 Helicase to the Replisome
Source: Mol Cell. 2016 Aug 4;63(3):371–84. doi: 10.1016/j.molcel.2016.05.036 (PMC4980427; doi:10.1016/j.molcel.2016.05.036)
Supplement: Document S1. Supplemental Experimental Procedures, Figures S1–S7, and Table S1 [file mmc1.pdf]

Molecular Cell, Volume *63*

Supplemental Information

Ctf4 Links DNA Replication with Sister Chromatid Cohesion Establishment by Recruiting the Chl1 Helicase to the Replisome

Catarina P. Samora, Julie Saksouk, Panchali Goswami, Ben O. Wade, Martin R. Singleton, Paul A. Bates, Armelle Lengronne, Alessandro Costa, and Frank Uhlmann

Figure S1

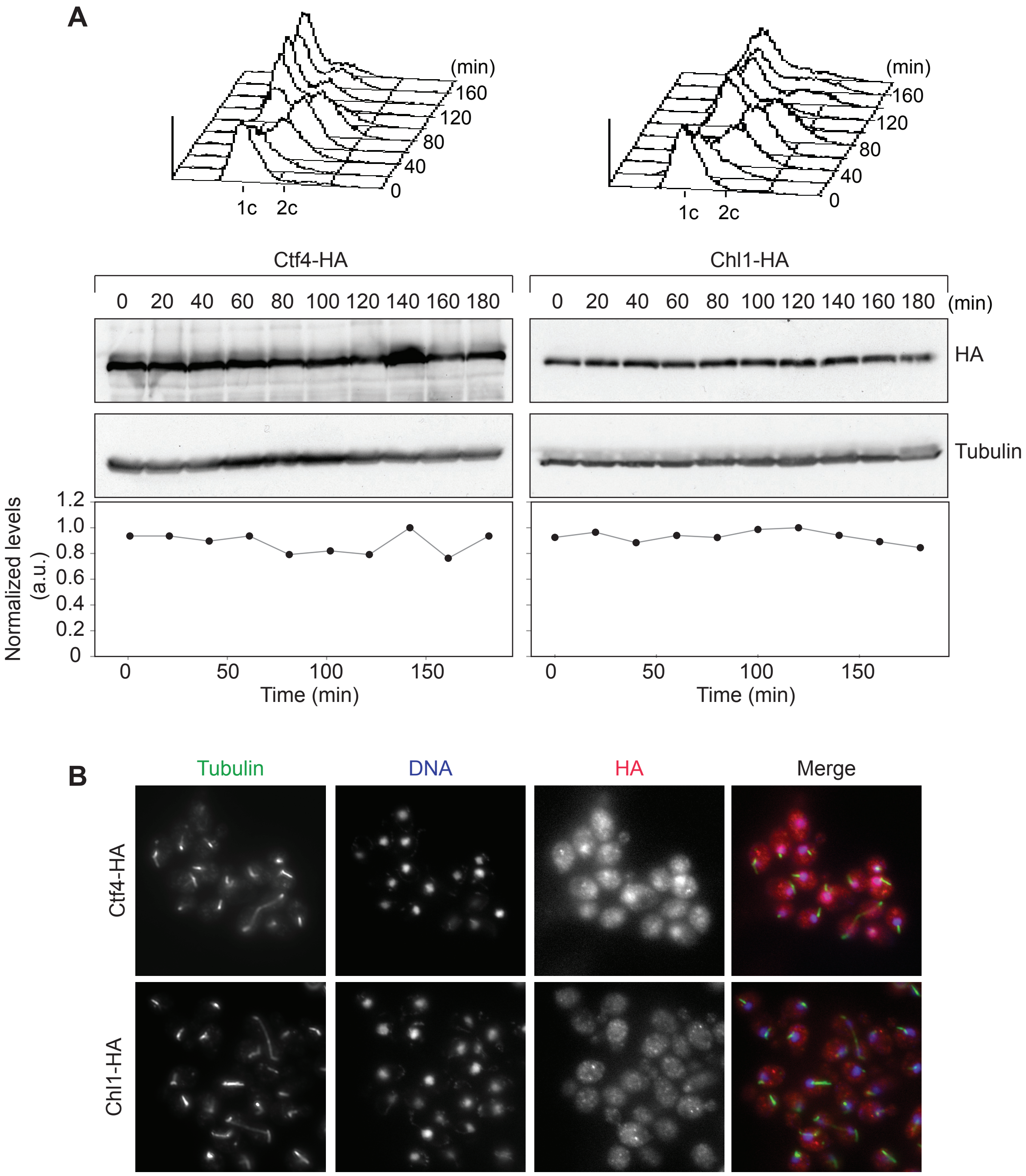
A
(min)
160
120
80
40
0
1c
2c
Ctf4-HA
Chl1-HA
0 20 40 60 80 100 120 140 160 180 (min)
HA
Tubulin
Normalized levels (a.u.)
1.2
1.0
0.8
0.6
0.4
0.2
0
0 50 100 150
Time (min)
B
Tubulin
DNA
HA
Merge
Ctf4-HA
Chl1-HA

Figure S2

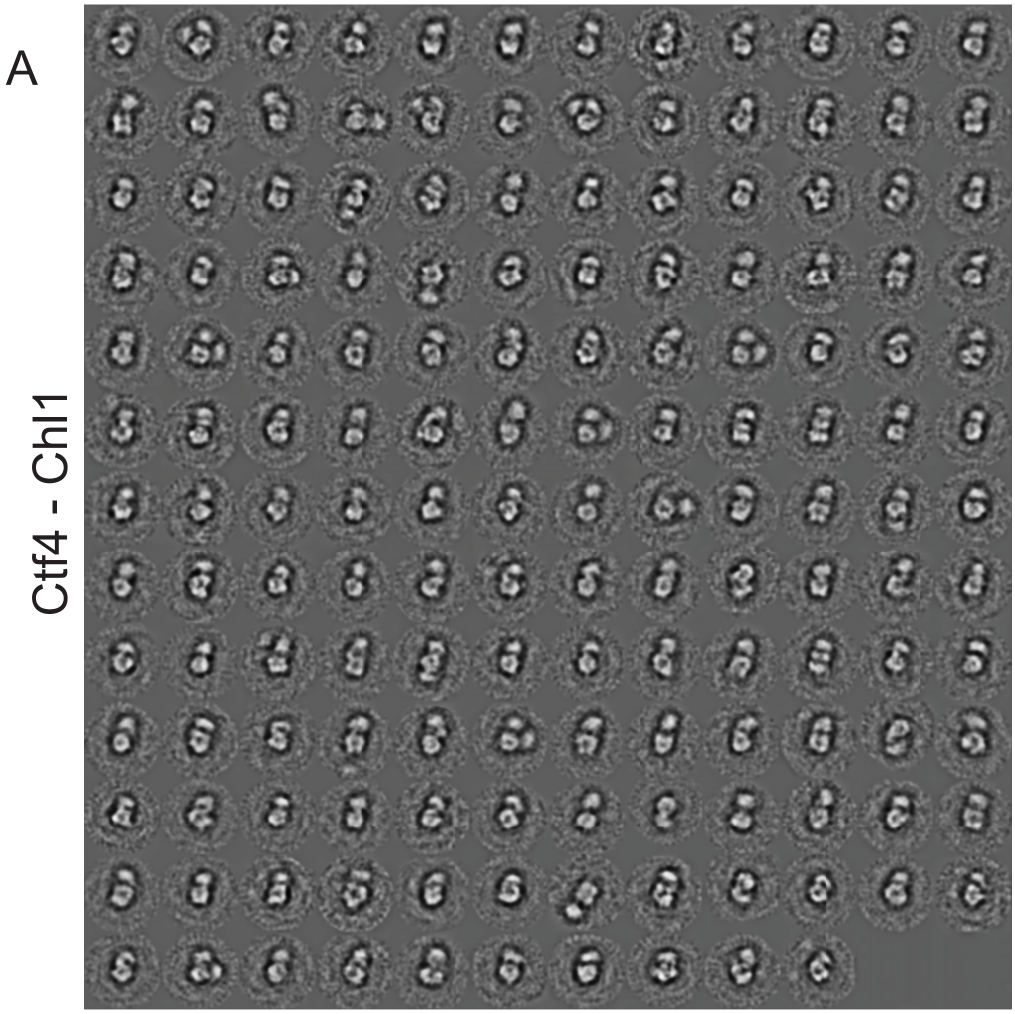

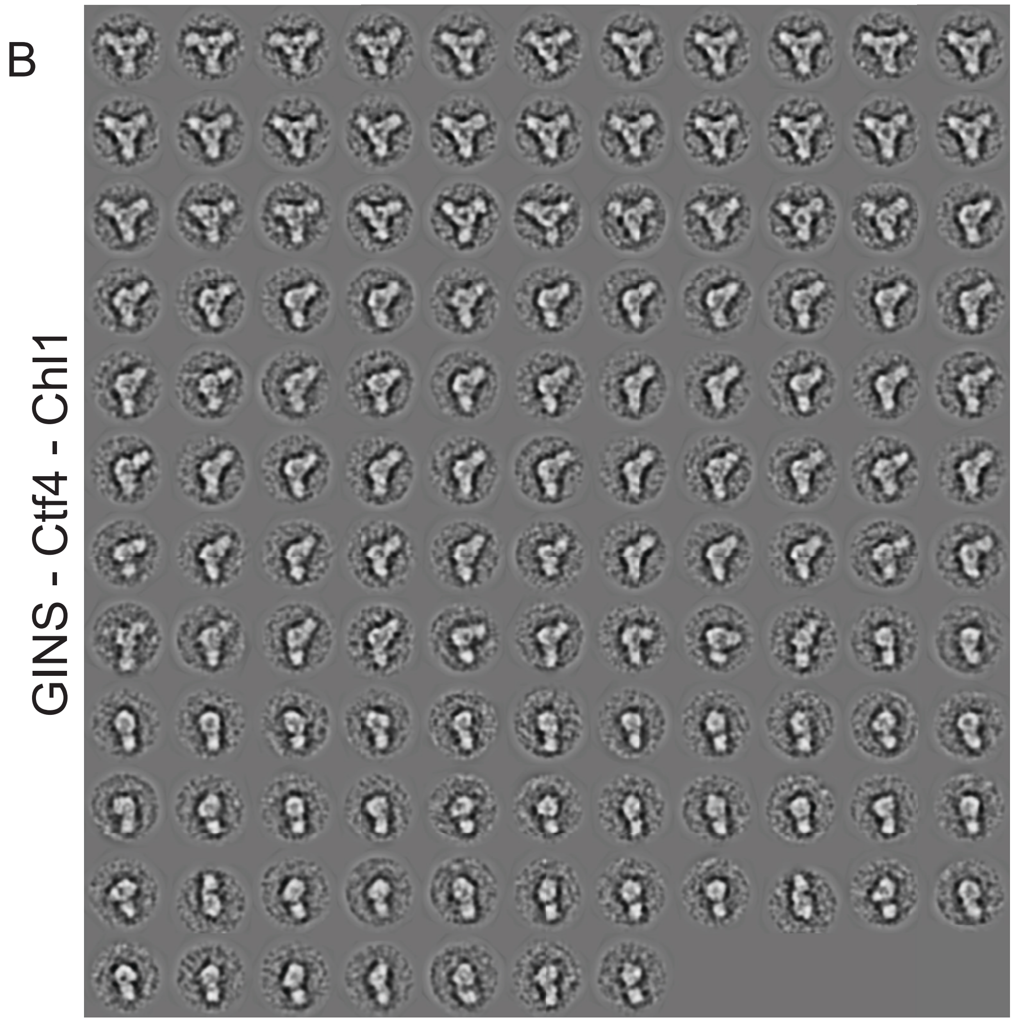

Figure S3

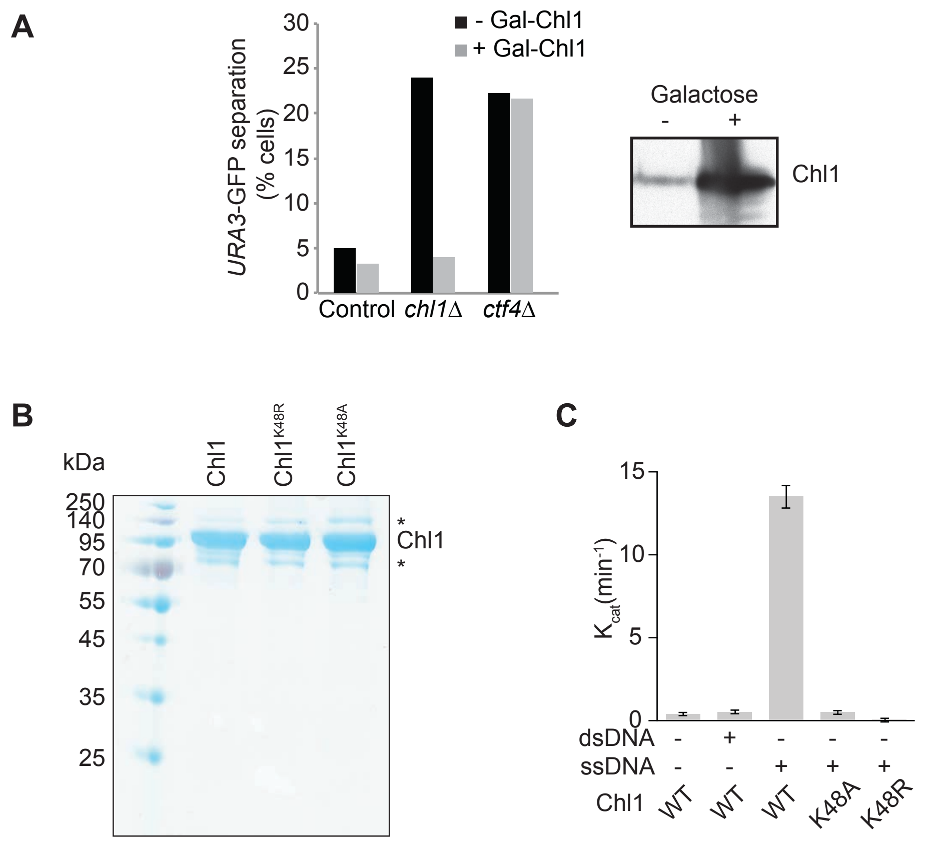
A
- Gal-Chl1
+ Gal-Chl1
URA3-GFP separation (% cells)
30
25
20
15
10
5
0
Control
chl1Δ
ctf4Δ
Galactose
-
+
Chl1
B
kDa
Chl1
Chl1K48R
Chl1K48A
250
140
95
70
55
45
35
25
*
Chl1
*
C
Kcat(min-1)
15
10
5
0
dsDNA - + - - -
ssDNA - - + + +
Chl1 WT WT WT K48A K48R

Figure S4

```
Chl1(1-60)          MDKKEYSETFYHPYKPYDIQVQLMETVYRVLSEGKKIAILESPTGTGKTLSLICATMTWL
mini-Chl1(1-52)     MDKKEYSETFYHPYKPYDIQVQLMETVYRVLSEGKKIAILESPTGTGKTLSL--------
XPD(23-40)          -----------------------------------YGVALESPTGSGKTIMAL-------

Chl1(61-120)        RMNKADIFTRMETNIKTNEDDSENLSDDEPDWVIDTYRKSVLQEKVDLLNDYEKHLNEIN
mini-Chl1(53-59)    --KSALQYS---------------------------------------------------
XPD(41-48)          --KSALQYS---------------------------------------------------

Chl1(121-180)       TTSCKQLKTMCDLDKEHGRYKSVDPLRKKRKGARHLDVSLEEQDFIPRPYESDSENNDTS
mini-Chl1           ------------------------------------------------------------
XPD                 ------------------------------------------------------------

Chl1(181-240)       KSTRGGRISDKDYKLSELNSQIITLLDKIDGKVSRDPNNGDRFDVTNQNPVKIYYASRTY
mini-Chl1(60-73)    ----------------------------------------------SERKLKIYYASRTY
XPD(48-61)          ----------------------------------------------SERKLKVLYLVRTN

Chl1(241-300)       SQLGQFTSQLRLPSFPSSFRDKVPDEKVKYLPLASKKQLCINPKVMKWKTLEAINDACAD
mini-Chl1(74-133)   SQLGQFTSQLRLPSFPSSFRDKVPDEKVKYLPLASKKQLCINPKVMKWKTLEAINDACAD
XPD(62-111)         SQEEQVIKELRSLSS---------TMKIRAIPMQGRVNMCILYRMVD-DLHEINAESLAK

Chl1(301-351)       LRHSKEGCIFYQNTNE---WRHCPDTLALR------DMIFSEIQDIEDLVPLGKSLGICP
mini-Chl1(134-184)  LRHSKEGCIFYQNTNE---WRHCPDTLALR------DMIFSEIQDIEDLVPLGKSLGICP
XPD(112-165)        ------FCNMKKREVMAGNEAACPYFNFKIRSDETKRFLFDELPTAEEFYDYGERNNVCP

Chl1(352-407)       YYASREALPIAEVVTLPYQYLLS----ESTRSSLQINLENSIVIIDEAHNLIETINSIYS
mini-Chl1(185-240)  YYASREALPIAEVVTLPYQYLLS----ESTRSSLQINLENSIVIIDEAHNLIETINSIYS
XPD(166-225)        YESMKAALPDADIVIAPYAYFLNRSVAEKFLSHWGVSRNQIVIILDEAHNLPDIGRSIGS

Chl1(408-466)       SQISLEDLKNCHKGIVTYFN-KFKSRLNPGNRVNLLKLNSLLMTLIQFIVKNFKKIGQEI
mini-Chl1(241-299)  SQISLEDLKNCHKGIVTYFN-KFKSRLNPGNRVNLLKLNSLLMTLIQFIVKNFKKIGQEI
XPD(226-280)        FRISVESLNRADREAQAYGDPELSQKIHVSDLIEMIRS-----ALQSMVSERCGKGDVRI

Chl1(467-526)       DPNDMFTGSNIDTLNIHKLLRYIKVSKIAYKIDTYNQALKEEESSKNENPIKETHKXSVS
mini-Chl1(300-359)  DPNDMFTGSNIDTLNIHKLLRYIKVSKIAYKIDTYNQALKEEESSKNENPIKETHKGSVS
XPD(281-333)        RFQEFMEYMRIMNKRSEREIRSLLN--YLYLFGEYVENEKEKVG-----KVPFSYCSSVA

Chl1(527-586)       SQPLLFKVSQFLYCLTNLTSEGQFFXEKNYSIKYMLLEPSKPFESILNQAKCVVLAGGTM
mini-Chl1(360-419)  SQPLLFKVSQFLYCLTNLTSEGQFFGEKNYSIKYMLLEPSKPFESILNQAKCVVLAGGTM
XPD(334-385)        SRIIAFSDQDEEKYAAILSPE------DGGYMQAACLDPSG-ILEVLKESKTIHMSG-TL

Chl1(587-646)       EPMSEFLSNLLPEVPSEDITTLSCNHVIPKENLQTYITNQPELEFTFEKRMSPSLVNNHL
mini-Chl1(420-479)  EPMSEFLSNLLPEVPSEDITTLSCNHVIPKENLQTYITNQPELEFTFEKRMSPSLVNNHL
XPD(386-436)        DPFDFYSDITGFEIPFKKIG-----EIFPPE--NRYIAYYDGVSSKYDTLDEKELDR--M

Chl1(647-706)       FQFXVDLSKAVPKKGGIVAFXPSYQYLAHVIQCWKQNDRFATLNNVRKIFYEAKDGDDIL
mini-Chl1(480-539)  FQFGVDLSKAVPKKGGIVAFGPSYQYLAHVIQCWKQNDRFATLNNVRKIFYEAKDGDDIL
XPD(437-484)        ATVIEDIILKVKKN--TIVYFPSYSLMDRVENRVS----FEHMKEYRGIDQKE------L

Chl1(707-766)       SGYSDSVAEGRGSLLLAIVGGKLSEGINFQDDLCRAVVMVGLPFXNIFSGELIVKRKHLA
mini-Chl1(540-599)  SGYSDSVAEGRGSLLLAIVGGKLSEGINFQDDLCRAVVMVGLPFGNIFSGELIVKRKHLA
XPD(485-538)        YSMLKKFRRDHG-TIFAVSGGRLSEGN-----ELEMIILAGLPFP-----RPDAINRSLF

Chl1(767-826)       AKIMKSGGTEEEASRATKEFMENICMKAVNQSVGRAIRHANDYANIYLLDVRYNRPNFRK
mini-Chl1(600-659)  AKIMKSGGTEEEASRATKEFMENICMKAVNQSVGRAIRHANDYANIYLLDVRYNRPNFRK
XPD(539-590)        DYYERKYGKGWEYSVVYPTAIK------IRQEIGRLIRSAEDTGACVILDKRAG--QFRK

Chl1(827-861)       KLSRWVQDSINSEHTTHQVISSTRKFFXMRSLNSR
mini-Chl1(660-694)  KLSRWVQDSINSEHTTHQVISSTRKFFGMRSLNSR
XPD(591-615)        FIP--------DMKKTSDPASDIYNFFISAQAR--
```

Figure S5

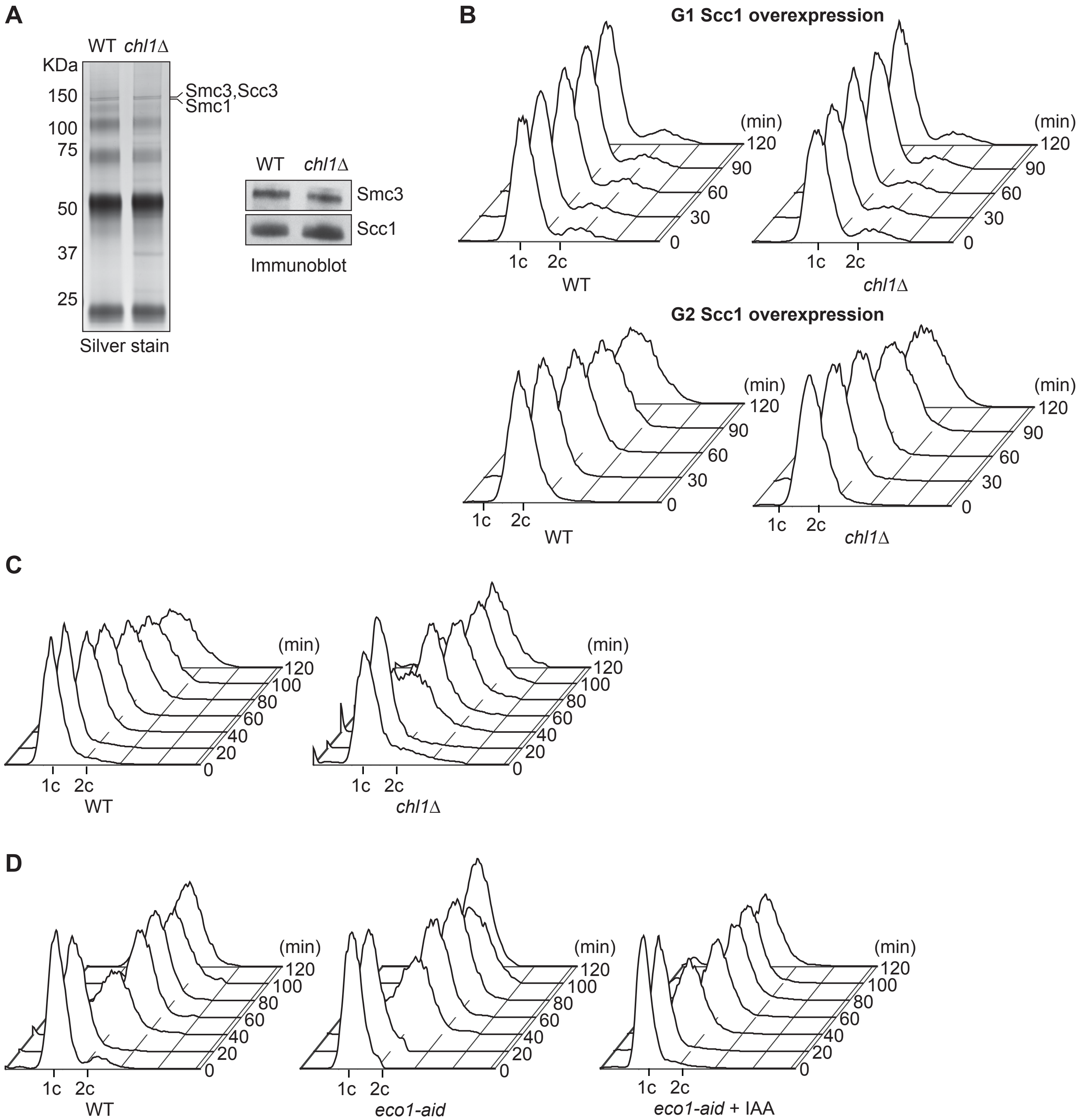
A
WT chl1Δ
KDa
150
100
75
50
37
25
Smc3,Scc3
Smc1
Silver stain
WT chl1Δ
Smc3
Scc1
Immunoblot
B
G1 Scc1 overexpression
(min)
120
90
60
30
0
1c 2c
WT
chl1Δ
G2 Scc1 overexpression
C
(min)
120
100
80
60
40
20
0
1c 2c
WT
chl1Δ
D
WT
eco1-aid
eco1-aid + IAA

Figure S6

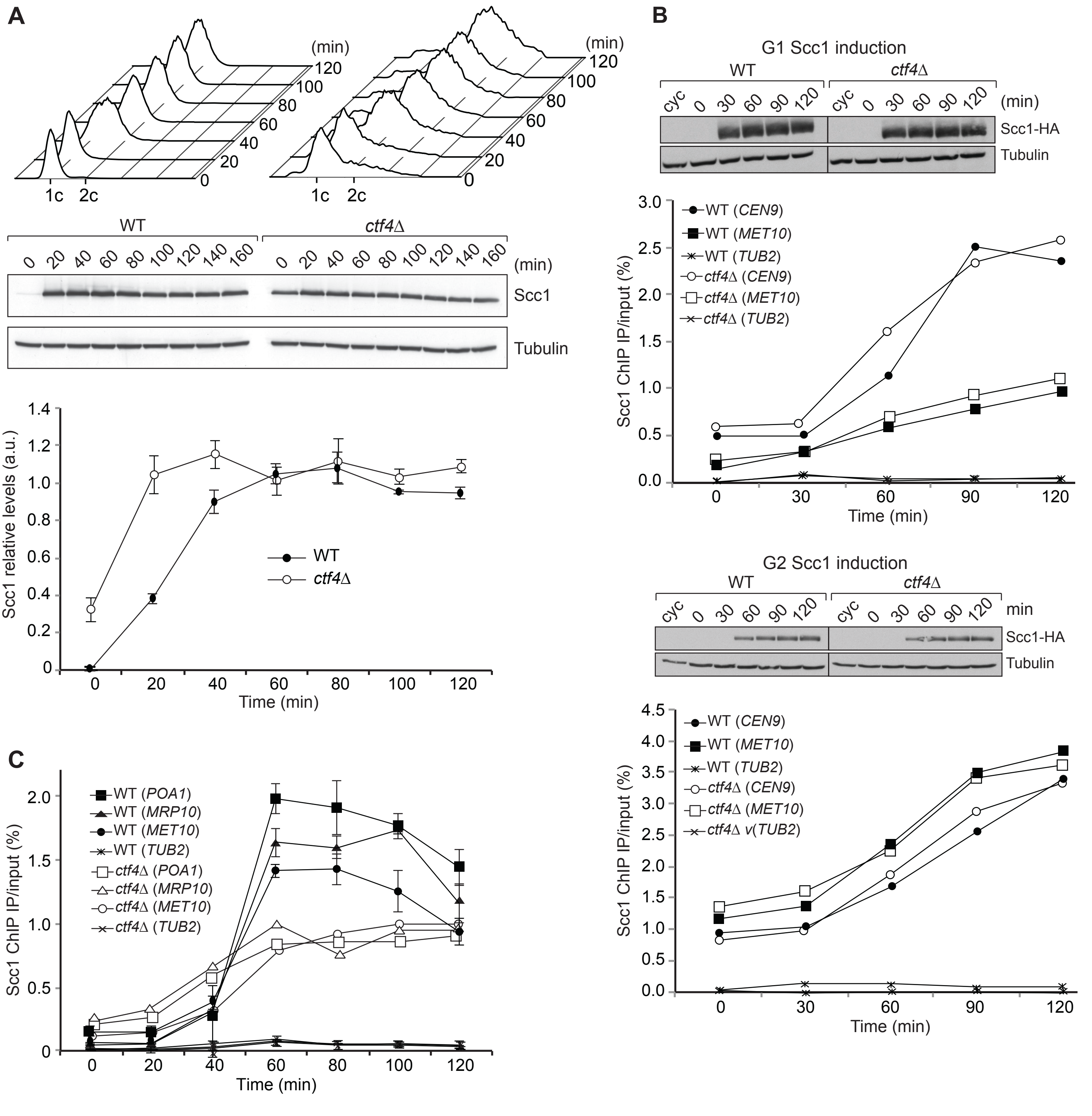

A
(min)
120
100
80
60
40
20
0
1c
2c
WT
ctf4Δ
0 20 40 60 80 100 120 140 160
(min)
Scc1
Tubulin
Scc1 relative levels (a.u.)
Time (min)
WT
ctf4Δ
B
G1 Scc1 induction
WT
ctf4Δ
cyc 0 30 60 90 120
(min)
Scc1-HA
Tubulin
WT (CEN9)
WT (MET10)
WT (TUB2)
ctf4Δ (CEN9)
ctf4Δ (MET10)
ctf4Δ (TUB2)
Scc1 ChIP IP/input (%)
Time (min)
G2 Scc1 induction
min
ctf4Δ v(TUB2)
C
WT (POA1)
WT (MRP10)
WT (MET10)
WT (TUB2)
ctf4Δ (POA1)
ctf4Δ (MRP10)
ctf4Δ (MET10)
ctf4Δ (TUB2)
Scc1 ChIP IP/input (%)
Time (min)

Figure S7

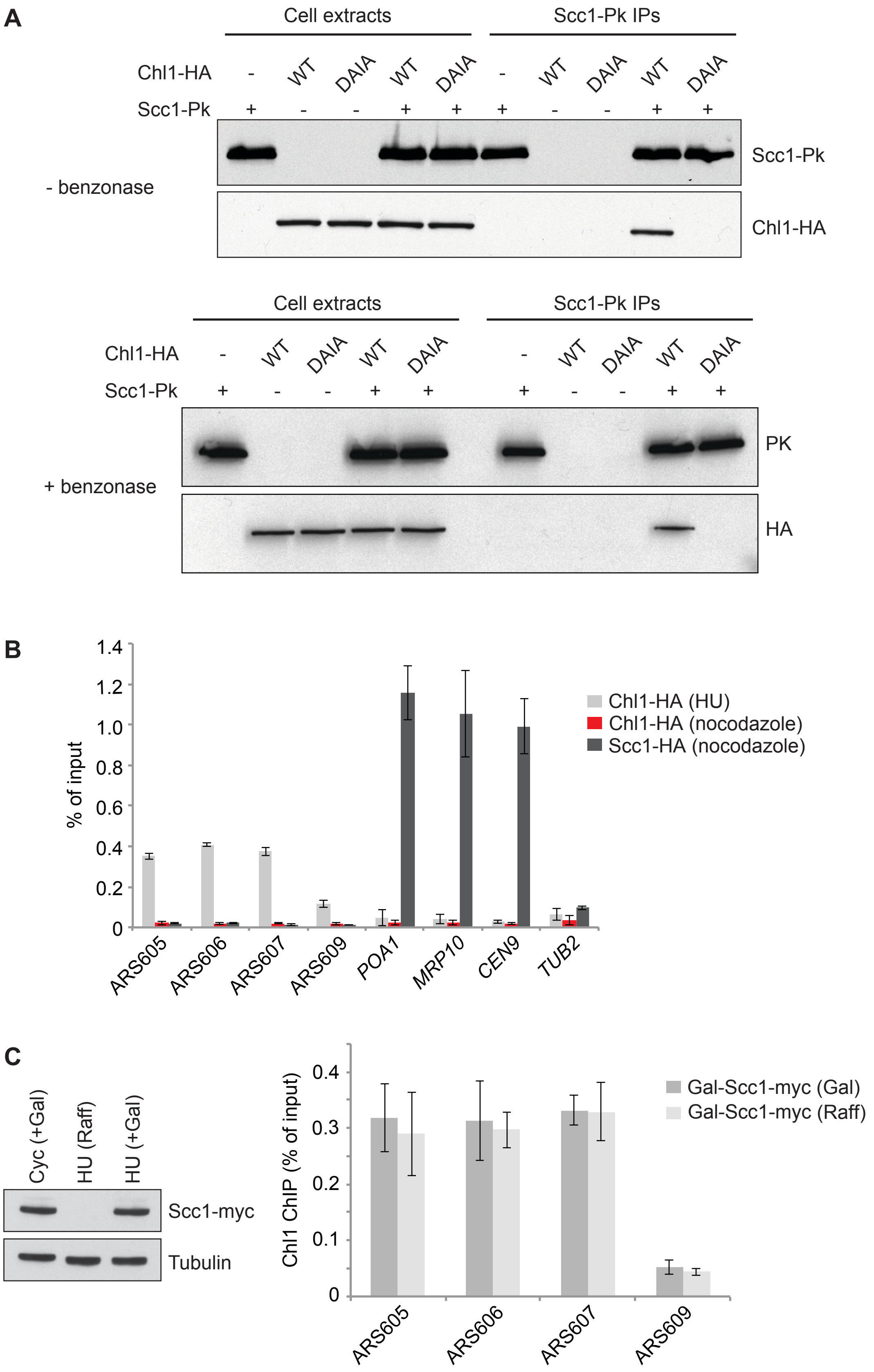
A
Cell extracts
Scc1-Pk IPs
Chl1-HA
Scc1-Pk
- WT DAIA WT DAIA - WT DAIA WT DAIA
+ - - + + + - - + +
Scc1-Pk
Chl1-HA
- benzonase
PK
HA
+ benzonase
B
% of input
Chl1-HA (HU)
Chl1-HA (nocodazole)
Scc1-HA (nocodazole)
ARS605
ARS606
ARS607
ARS609
POA1
MRP10
CEN9
TUB2
C
Cyc (+Gal)
HU (Raff)
HU (+Gal)
Scc1-myc
Tubulin
Chl1 ChIP (% of input)
Gal-Scc1-myc (Gal)
Gal-Scc1-myc (Raff)
ARS605
ARS606
ARS607
ARS609

SUPPLEMENTAL FIGURE LEGENDS

Figure S1. Constant Ctf4 and Chl1 Levels and Localization throughout the Cell Cycle, Related to Figure 1

(A) Ctf4 and Chl1 levels remain constant throughout the cell cycle. Proteins were detected by quantitative immunobloting against a C-terminal HA epitope tag in extracts from cells blocked in G1 by α-factor treatment and released to progress through a synchronous cell cycle. Cell cycle progression was monitored by FACS analysis of DNA content, tubulin served as a loading control.

(B) Unchanged localization of Ctf4 and Chl1 during the cell cycle. Asynchronously growing cells were fixed and stained for the HA epitope tag on Ctf4 and Chl1, respectively. DNA was stained using DAPI, tubulin staining was used to assign cell cycle stages. Fields with cells at different stages of the cell cycle are shown.

Figure S2: Single-Particle Electron Microscopy Analysis, Related to Figure 3

(A) Two dimensional reference free class averages of the reconstituted Chl1-Ctf4 complex are shown.

(B) as (A), class averages of the Chl1-Ctf4-GINS assembly are shown. Box size is 441 Å x 441 Å.

Figure S3. Chl1 Overexpression Cannot Rescue Sister Chromatid Cohesion in the Absence of Ctf4 and Biochemical Characterization of ATPase-Deficient Chl1, Related to Figure 4

(A) Chl1 depends on Ctf4 for its function in sister chromatid cohesion. Cells of the indicated genotype were synchronized in G1, released, and arrested in metaphase by nocodazole treatment. Chl1 was overexpressed from the *GAL1* promoter for 12 hours prior to the experiment (+Gal-Chl1) or was not expressed (-Gal-Chl1). Sister chromatid cohesion at the GFP-marked *URA3* locus was analyzed. Overexpression was verified by immunoblotting against the HA epitope tag on Chl1. Tubulin served as a loading control.

(B) Purification of recombinant wild type Chl1, Chl1^{K48R} and Chl1^{K48A}. Aliquots of the purified proteins were analyzed by SDS-PAGE followed by Coomassie Blue staining. Two contaminants are marked by asterisks.

(C) Chl1^{K48R} and Chl1^{K48A} do not retain detectable ssDNA-dependent ATPase activity. A continuous ATP-NADH coupled spectrophotometric assay was performed, as described in the Experimental Procedures, to assess ssDNA-dependent ATP hydrolysis by the three Chl1 proteins.

Figure S4. Construction of mini-Chl1, Related to Figure 5

Alignment of the mini-Chl1 protein construct with the Chl1 sequence and the sequence of the structural template used for modelling, *T. acidophium* XPD. Highlighted are the ATPase motifs (red), the Chl1-specific insertion (purple), the 4 cysteines that coordinate the iron sulfur cluster (yellow) and the CIP box (blue).

Figure S5. Cohesin Complex Integrity Is Unaffected in *chl1Δ* Cells and Confirmation of Cell Synchrony, Related to Figure 6

(A) Pk epitope-tagged Scc1 was immunoprecipitated from extracts of the indicated strains, synchronized in early S-phase by HU treatment. Immunoprecipitates were analyzed by silver staining (left panel) or immunoblotting against the HA epitope tag of Smc3 (right panel).

(B - D) FACS analysis of DNA content of the cultures used for the experiments shown in Figures 6B, C and D, respectively.

Figure S6. Ctf4, like Chl1, Is Required for Chromosomal Cohesin Accumulation during S-phase, Related to Figure 6

(A) Scc1 levels are not reduced in cells lacking Ctf4. The experiment was conducted as in Figure 6A, but wild type and *ctf4Δ* cells were compared.

(B) Ectopic cohesin loading onto chromosomes is independent of Ctf4. The experiment was conducted as in Figure 6B, but wild type and *ctf4Δ* cells were compared. All immunoblot samples were run on the

same gel, separated by additional lanes where indicated.

(C) Ctf4 augments cohesin association with chromosomes during S-phase. The experiment was conducted as in Figure 6C, but wild type and *ctf4Δ* cells were compared.

Figure S7. Chl1 Interacts with the Cohesin Complex at the Replication Fork, Related to Figure 7

(A) The cohesin-Chl1 interaction is CIP Box-dependent and benzonase-insensitive. Pk epitope-tagged Scc1 was immunoprecipitated and coprecipitation of Chl1 was analyzed by immunoblotting. Whole cell extracts and immunoprecipitates of strains of the indicated genotype were prepared from cultures arrested in early S-phase by HU treatment as described in Figure 7C. Benzonaze was omitted (top panel) or added (bottom panel) to the extracts prior to immunoprecipitation.

(B) Chl1 is enriched in replicating regions during S phase, but not cohesin binding sites. ChIP was performed against Chl1 or cohesin in cells synchronized in early S-phase (HU) or mitosis (nocodazole). Enrichment close to replication origins (ARS605, 606 and 607) and known cohesin binding sites (*POA1*, *MRP10* and *CEN9*) was analyzed by quantitative real time PCR. ARS609 (an inactive origin in HU) and *TUB2* (a gene where cohesin is not enriched) served as negative controls.

(C) Chl1 binds to replication forks independently of cohesin. Cells in which Scc1 expression is under control of the *GAL1* promoter were grown in medium containing raffinose and galactose. Half of the culture was filtered and shifted to medium containing raffinose only at the time when α-factor was added to synchronize cells in G1. Cells were then released into HU containing medium and aliquots processed for Western blotting to confirm the presence or absence of Scc1 and for ChIP against Chl1. Enrichment at three active and an inactive origin was assessed.

Table S1. Yeast strains used in this study (all Strains were of the W303 background)

Figure 1

(A) Y3615 *MAT***a** *CHL1-*Pk_3*::TRP1 CTF4-*HA_6*::HIS3*

(B, C) Y959 *MAT***a** *ura3::URA3/GPD-TK(7x)*
Y3188 *MAT***a** *CTF4-*Pk_9*::TRP1*
Y4642 *MAT***a** HA_6*-CHL1 (N-terminal tag)*

Figure 2

(B) Y3188 as above
Y4916 *MAT***a** *CHL1-*HA_3*::LEU2*
Y4694 *MAT***a** $chl1^{DAIA}$*-*HA_3*::LEU2*
Y4917 *MAT***a** $chl1^{K48R}$*-*HA_3*::LEU2*
Y4693 *MAT***a** *CTF4-*Pk_9*::TRP1 CHL1-*HA_3*::URA3*
Y4826 *MAT***a** *CTF4-*Pk_9*::HIS3* $chl1^{DAIA}$*-*HA_3*::LEU2*
Y4918 *MAT***a** *CTF4-*Pk_9*::HIS3* $chl1^{K48R}$*-*HA_3*::LEU2*

(C) Y3188 as above
Y4390 *MAT***a** *CTF4-*Pk_9*::TRP1 chl1Δ::LEU2*

(D) Y4916 as above
Y3617 *MATa CHL1-*HA_3*::HIS3 ctf4Δ::TRP*1
Y4694 as above
Y4917 as above

Figure 3

(A) Y4693 as above

Figure 4

(A) K7100 *MAT***a** *ura3::3xURA3::tetO112 his3::HIS3::tetR-GFP*
Y4161 *MAT***a** *ura3::3xURA3::tetO112 his3::HIS3::tetR-GFP ctf4Δ::TRP1*
Y3856 *MAT***a** *ura3::3xURA3::tetO112 his3::HIS3::tetR-GFP chl1Δ::TRP1*
Y4691 *MAT***a** *ura3::3xURA3::tetO112 his3::HIS3::tetR-GFP* $chl1^{DAIA}$*-*HA_3*::LEU2*
Y4919 *MAT***a** *ura3::3xURA3::tetO112 his3::HIS3::tetR-GFP* $chl1^{K48R}$*-*HA_3*::LEU2*
Y4920 *MAT***a** *ura3::3xURA3::tetO112, his3::HIS3::tetR-GFP* $chl1^{K48A}$*-*HA_3*::LEU*2

(B) A2085 *MAT***a** *ura3::URA3/GPD-TK(5x) AUR1c::ADH-hENT1*
A2252 *MAT***a** *ura3::URA3/GPD-TK(5x) AUR1c::ADH-hENT1 ctf4Δ::HIS3*
A2253 *MAT***a** *ura3::URA3/GPD-TK(5x) AUR1c::ADH-hENT1 chl1Δ::HIS3*

(C) A872 *ura3::URA3/GPD-TK(7x) RAD5*
A908 *ura3::URA3/GPD-TK(7x) RAD5 ctf4Δ::HIS3*
A911 *ura3::URA3/GPD-TK(7x) RAD5 chl1Δ::HIS3*

(D) Y141 W303 wild type

Y4323 *MAT***a** *ctf4Δ::TRP1*

Y4324 *MAT***a** *chl1Δ::TRP1*

Y4694 as above

Y4917 as above

Figure 5

(B) Y4916 as above

Y4921 *MAT***a** *miniCHL1-HA*$_3$*::LEU2*

(C) K7100 as above

Y3856 as above

Y4922 *MAT***a** *ura3::3xURA3::tetO112 his3::HIS3::tetR-GFP miniCHL1-HA*$_3$*::LEU2*

(D) Y141 as above

Y4323 as above

Y4324 as above

Y4921 as above

Figure 6

(A) Y2269 *MAT***a** *SCC1-Pk*$_9$*::TRP1*

Y4389 *MAT***a** *SCC1-Pk*$_9$*::TRP1 chl1Δ::LEU2*

(B) Y4380 *MAT***a** *ura3::3xURA3::tetO112 his3::HIS3::tetR-GFP GAL-SCC1-HA*$_3$*::LEU2*

Y4386 *MAT***a** *ura3::3xURA3::tetO112 his3::HIS3::tetR-GFP GAL-SCC1-HA*$_3$*::LEU2 chl1Δ::TRP1*

Y4923 *MAT***a** *GAL-SCC1(R180D,R268D)-HA*$_3$*::LEU2*

Y4924 *MAT***a** *GAL-SCC1(R180D,R268D)-HA*$_3$*::LEU2 chl1Δ::HIS3*

(C) Y2269 as above

Y4389 as above

(D) Y4766 *MAT***a** *pADH1-OsTir1-myc*$_9$*::ADE2 SCC1-6E2::TRP1 HIS3::pMET3-eco1-IAA17::KAN*^{R}

Y4925 *MAT***a** *SCC1-6E2::TRP1*

Figure 7

(A) Y2012 *MAT***a** *scc1-73 ura3::3xURA3::tetO112 his3::HIS3::tetR-GFP GAL-SCC1-HA*$_3$*::LEU2*

Y4384 *MAT***a** *ura3::3xURA3::tetO112 his3::HIS3::tetR-GFP GAL-SCC1-HA*$_3$*::LEU2 ctf4Δ::TRP1*

Y4386 as above

Y4383 *MAT***a** *ura3::3xURA3::tetO112 his3::HIS3::tetR-GFP GAL-SCC1-HA*$_3$*::LEU2 mrc1Δ::TRP1*

Y4385 *MAT***a** *ura3::3xURA3::tetO112 his3::HIS3::tetR-GFP GAL-SCC1-HA*$_3$*::LEU2 ctf18Δ::TRP1*

Y4382 *MAT***a** *ura3::3xURA3::tetO112 his3::HIS3::tetR-GFP GAL-SCC1- HA*$_3$*::LEU2 tof1Δ::TRP1*

Y4381 *MAT***a** *ura3::3xURA3::tetO112 his3::HIS3::tetR-GFP GAL-SCC1-*HA_3*::LEU2 csm3Δ::TRP1*

(B) Y141 as above

Y4324 as above

Y4386 as above

(C, D) Y2269 as above

Y4916 as above

Y4926 *MAT***a** *Scc1-*Pk_9*::TRP1 CHL1-*HA_3*::LEU2*

Y4927 *MAT***a** *Scc1-*Pk_9*::TRP1 CHL1-*HA_3*::LEU2 ctf4Δ::HIS3*

(E) Y5040 *MAT***a** *Scc1-*HA_6*::TRP1 CTF4-*Pk_9*::HIS3*

Y4927 *MAT***a** *Scc1-*HA_6*::TRP1 CTF4-*Pk_9*::HIS3 chl1Δ::LEU2*

Figure S1

(A) Y1451 *MAT***a** *CTF4-*HA_6*::HIS3*

Y3613 *MAT***a** *CHL1-*HA_6*::HIS3*

(B) K1451 as above

Y3613 as above

Figure S3

(A) Y4928 *MAT***a** *ura3::3xURA3::tetO112 his3::HIS3::tetR-GFP GAL-CHL1-*HA_3*::LEU2*

Y4929 *MAT***a** *ura3::3xURA3::tetO112 his3::HIS3::tetR-GFP GAL-CHL1-*HA_3*::LEU2 chl1Δ::TRP1*

Y4930 *MAT***a** *ura3::3xURA3::tetO112 his3::HIS3::tetR-GFP GAL-CHL1-*HA_3*::LEU2 ctf4Δ::TRP1*

Figure S5

(A) Y4931 *MAT***a** *SCC1-*Pk_9*::TRP1 SMC3-*HA_6*::HIS3*

Y4932 *MAT***a** *SCC1-*Pk_9*::TRP1 SMC3-*HA_6*::HIS3 chl1Δ::LEU2*

Figure S6

(A) Y4388 *MAT***a** *SCC1-*Pk_9*::TRP1 ctf4Δ::LEU2*

(B) Y4384 as above

Y4933 *MAT***a** *GAL-SCC1(R180D,R268D)-*HA_3*::LEU2 ctf4Δ::HIS3*

(C) Y4388 as above

Figure S7

(A) Y2269 as above

Y4916 as above

Y4694 as above

Y4926 as above

Y4934 *MAT***a** $Scc1\text{-}Pk_9$*::TRP1* $chl1^{DAIA}\text{-}HA_3$*::LEU2*

(B) Y4642 as above

Y4220 *MAT***a** $Scc1\text{-}HA_6$*::TRP1*

(C) Y5041 *MAT***a** *scc1Δ::URA3* $GAL\text{-}SCC1myc_{18}$ $CHL1\text{-}HA_6$*::HIS3*

Supplemental Experimental Procedures

Yeast Strains and Culture

All strains used in this study were derivatives of W303 and are listed in Table S1. Gene deletions and epitope tagging of endogenous genes were performed by gene targeting using polymerase chain reaction (PCR) products (Knop et al., 1999; Wach et al., 1994). Cells were grown in rich YP medium or in complete synthetic medium lacking methionine, supplemented with either 2% glucose or 2% raffinose as the carbon source (Amberg et al., 2005). To induce gene expression from the *GAL1* promoter, 2% galactose was added to cells grown in raffinose-containing medium. Overexpression of Scc1 in G1 was achieved by *GAL1* promoter-driven expression of a non-cleavable variant, Scc1(R180D,R268D) (Uhlmann et al., 1999). Cells were synchronized in G1 by adding α-factor (0.04 mg/ml) for 2 hours. To arrest cells in early S-phase, G1 synchronized cultures were released from α-factor block into medium containing 0.1 M hydroxyurea (HU) for 60 minutes. Arrest in G2/M was achieved by release into medium containing 5 mg/ml nocodazole for 120 minutes. When cells were released to pass through a complete, synchronous cell cycle, α-factor was readded to the culture, once cells started budding, for re-arrest in the following G1. To inactivate Eco1, its gene promoter was replaced with the methionine-repressible *MET3* promoter and its C-terminus fused to an auxin-inducible degron (Nishimura et al., 2009). Cells were grown in medium lacking methionine and shifted to medium containing methionine and 500 μM of the auxin indole-3-acetic acid (IAA) 1 hour before release from α-factor block into synchronous cell cycle progression. Strains expressing $Chl1^{DAIA}$, $Chl1^{K48R}$ or $Chl1^{K48A}$ were engineered by endogenous gene replacement using an integrative targeting plasmid. To generate the mini-Chl1 derivative, amino acids 53-231 in Chl1 were replaced by the amino acid sequence KSALQYSSERKL (compare Figure S4).

Yeast Molecular Biology Techniques

Coimmunoprecipitation assays were performed as described (Godfrey et al., 2015), except that cell extracts were prepared using buffer containing 100 mM Hepes-KOH (pH 7.9), 300 mM potassium acetate, 10 mM magnesium acetate, 10% glycerol, 0.1% NP-40, 2 mM EDTA, 2 mM β-glycerophosphate, 2 mM sodium fluoride, 1 mM dithiothreitol, a protease inhibitor cocktail (Roche and Sigma) and treated with benzonase (Merck Biosciences), as described (Gambus et al., 2009). Antibodies used for immunoprecipitation and Western blotting were anti-Pk (clone SV5-Pk1, Serotec), anti-HA (clone F-7, Santa Cruz), anti-α-tubulin (clone YOL1/34, Serotec), anti-myc (clone 9E10), an antibody specific to acetylated Smc3 (Borges et al., 2010) and anti-Psf2 and anti-Pol1 antisera that were a kind gift from K. Labib. Quantitative analysis of immunoblots was performed with an ImageQuant LAS 4010 imager (GE Healthcare).

Chromatin immunoprecipitation and microarray analysis were performed as previously described (Lengronne et al., 2004), as was the quantitative analysis of chromatin immunoprecipitates (Lopez-Serra et al., 2014). Analysis of sister chromatid cohesion followed a published procedure (Michaelis et al., 1997).

Chl1 expression and purification and its use in ATPase assays

Wild type and mutant Chl1, fused via a TEV protease recognition sequence to a Strep-tag for affinity purification, were expressed from recombinant baculoviruses in High Five insect cells (Life Technologies). Harvested cells were sonicated in buffer containing 50 mM Tris-HCl (pH 8.5), 300 mM NaCl, 10% glycerol, 0.5 mM TCEP and centrifuged at 35000 x g at 4°C for 1 hour. The supernatant was loaded onto a 5 ml Strep-Tactin superflow plus cartridge (Qiagen) and eluted in buffer containing 1 mg/ml of desthiobiotin. The Strep-tag was cleaved off overnight at 4°C using 0.04 mg/ml TEV protease. The protein was then loaded onto a Superdex 200 10/300 GL column (GE Healthcare), equilibrated with 50 mM Tris-HCl (pH 8.5), 300 mM NaCl and 0.5 mM TCEP. Eluted protein-containing fractions were analyzed for purity by SDS polyacrylamide gel electrophoresis. To measure ATPase activity, a continuous ATP-NADH coupled spectrophotometric assay was performed. The change in absorbance at 340 nm was recorded for 10 minutes at 20°C in a reaction consisting of 100 mM Tris-HCl (pH 8.5), 300 mM NaCl, 10 mM $MgCl_2$, 1 mM ATP, 40 U/ml pyruvate kinase, 50 U/ml lactate dehydrogenase, 0.5 mM phosphoenolpyruvate, 0.2 mM NADH, with or without 3.5 μM (molecules) of DNA substrate. A 20mer ssDNA and 21mer dsDNA with sequences oligo d(T) and 5'-

TCTCCACAGGAAACGGAGGGT-3’ respectively (Sigma), were used as substrates. Chl1 was added to start the reaction at a concentration of 3.5 μM.

Protein Expression, Purification and Complex Reconstitution for Electron Microscopy

Ctf4 purification: N-terminally His-tagged *S. cerevisiae* Ctf4 (residues 471-927) was expressed in (DE3)-CodonPlus cells (Stratagene) transformed with a pRSFDuet-1-based construct (a kind gift of Luca Pellegrini) (Simon et al., 2014). Six liters of cells were grown in LB medium to an optical density of 0.6 before induction with 1 mM IPTG, and overnight expression at 20°C. The cleared lysate was incubated with 3 ml Ni-NTA resin (Qiagen), washed with 5 column volumes (CV) of buffer A (50 mM NaH_2PO_4 pH 8.0, 300 mM NaCl, 20 mM imidazole) and eluted with 5 ml of buffer A supplemented with 230 mM imidazole. The eluted protein was further purified by gel filtration chromatography over a Superdex 200 16/60 HiLoad column (GE Healthcare) in 25 mM HEPES-NaOH pH 7.0, 200 mM NaCl and 10% glycerol. Pooled fractions were concentrated to 5 mg/ml and stored at -80°C (in 2 nmol aliquots).

GINS purification: Full-length *S. cerevisiae* GINS was cloned into pET28c (Novagen) and carries an N-terminal Strep III tag on the Psf3 subunit (a kind gift of Karim Labib) (Gambus et al., 2009). This expression construct was transformed into BL21 (DE3)-CodonPlus cells. Lysates from 2 liters LB culture was incubated with with 3 ml StrepTactin resin (IBA Life Sciences), washed with five column volumes of buffer B (25 mM HEPES-NaOH pH 7.5, 300 mM NaCl, 10% Glycerol, 0.5 mM TCEP) and eluted with 4 ml buffer B supplemented with 2.5 mM desthiobiotin (IBA Life Sciences). Proteins were stored at -80°C at a concentration of 1 mg/ml (in 2 nmol aliquots).

Chl1 purification: pFastBac1 plasmids containing the budding yeast *CHL1* gene was used to produce a bacmid, which was subsequently transfected into Sf9 cells to generate recombinant baculovirus expressing Chl1, which was amplified over two rounds in Sf9 cells grown in Graces medium supplemented with 10% FCS. The infection for protein purification was carried out by inoculating 6 liters of Hi5 cells at 10^6 cells/ml by adding 50 ml of virus stock per liter of Hi5 cells. Infected cells were incubated for 48 hours at 27°C, harvested by centrifugation and washed with PBS + 5 mM $MgCl_2$. The collected cells were re-suspended in 200 ml of resuspension buffer (25 mM HEPES-NaOH pH 7.6, 0.02% Tween-20, 10% glycerol, 1 mM EDTA, 1 mM EGTA) supplemented with 15 mM KCl, 2 mM $MgCl_2$, 0.4 mM PMSF, 2 mM 2-mercaptoethanol and the complete protease inhibitors cocktail from Roche Diagnostics. The cell suspension was snap frozen in 10 ml aliquots and stored at -80°C. The cell suspension once thawed was broken using a Dounce homogenizer. This was followed by centrifugation at 14,000 rpm in an Avanti J-26S XP centrifuge for 10 minutes to clear the extract. The cleared extract was incubated with 4 ml of StrepTactin resin (IBA Life Sciences) for 2 hours with continuous end-over-end mixing. The beads were then collected in a 20 ml Poly-Prep disposable chromatography column (BioRad) and washed with 30 ml buffer C (50 mM Tris-HCl pH8.5, 300 mM NaCl, 10% Glycerol and 3 mM DTT). Bound complexes were eluted in buffer C with 2.5 mM desthiobiotin and complete protease inhibitor cocktail. Pooled elution fractions were further purified on a Mono Q (GE Healthcare) ion exchange column, pre-equilibrated in Buffer D (10 mM Tris-HCl pH 8.5, 100 mM NaCl and 3 mM DTT). Bound complexes were eluted with 10 ml of a 100-250 mM NaCl linear gradient and collected in 500 μl fractions. Peak fractions were pooled and dialyzed for 16 hours against 25 mM HEPES-NaOH pH 7.6, 150 mM NaCl, 2 mM DTT. Proteins were stored at -80 °C at a concentration of 5 mg/ml (in 2 nmol aliquots).

To reconstitute the Ctf4-client protein complexes, Ctf4, Chl1 and GINS preparations were first dialyzed separately to 25 mM HEPES-NaOH pH 7.6, 500 mM sodium acetate, 0.5 mM DTT for 1 h at 4 °C in dialysis tubes with a 6,000-8,000 Da molecular weight cutoff (GeBAflex). 2 nmol of each component were then coincubated for 10 minutes on ice in a 100 μl volume. The reconstitution mix was initially dialyzed in 400 mM sodium acetate, 25 mM HEPES-NaOH pH 7.6, 0.5 mM DTT for 1 h at 4°C. The dialysis buffer was changed hourly to contain progressively 300 mM, 200 mM and finally 150 mM sodium acetate. The reconstituted complexes were crosslinked with 0.1% glutaraldehyde for 20 minutes followed by deactivation with 80 mM Glycine.

Electron Microscopy and Single Particle Analysis

Carbon was evaporated onto freshly cleaved mica with a Q150TE coater (Quorum Technologies) and incubated overnight before coating 400 mesh grids (Agar Scientific). Dried carbon grids were glow discharged for 30–60 seconds at 45 mA using a 100x glow discharger (Electron Microscopy Sciences). A 4 μl drop of the reconstituted sample was applied onto the grid and incubated for 1 minute. Grids

were then sequentially stirred over five distinct 75 μl drops of 2% uranyl formate solution, 10 seconds per drop. The excess stain was blotted away until the grids were dry.

Image data from the negative stained grids were collected using a Tecnai LaB6 G2 Spirit transmission electron microscope (FEI) operating at 120 keV. A GATAN Ultrascan 100 camera at a nominal magnification of 30,000x (3.45 Å/pixel at the specimen level) was used to record images. For each dataset between 100 and 150 micrographs were collected. Particle picking, contrast transfer function estimation and correction were all performed using EMAN2.1 software. Multivariate statistical analysis-based classification was performed in Imagic, as previously described (Simon et al., 2014).

Replication Fork Speed Measurements

Replication fork progression was quantitatively analyzed following BrdU incorporation and DNA fibre combing, as previously described (Bianco et al., 2012).

Structural model of *S. cerevisiae* Chl1 and design of mini-Chl1

The 3-dimensional model and alignment of Chl1 were based on the crystal structure of *T. acidophilum* XPD (Wolski et al., 2008) and were generated by the program 3D-JIGSAW (Bates et al., 2001). A three-state protein secondary structure prediction for the Chl1-specific insert predicts it to consist of long helical and coiled segments and is therefore likely to be more prone to large conformational changes compared to the remainder of the Chl1 sub-domain structures, which have less coil segments along with stabilizing strands and, therefore, β-sheet architecture. Perhaps as a consequence of this likely higher degree of flexibility, the insert domain currently has no structural homology to an experimentally determined protein structure and could not be modeled with sufficient accuracy. The link used to connect the first region of the helicase, the region containing the N-terminal ATPase motif, GxGKT, is based on native sequence from XPD, which mainly consists of a helical segment, as visual inspection of a number of 3D alternative models indicated this to be the most parsimonious way to connect sub-domains while maintaining their native packing geometry. Alignment of the motifs required for ATPase activity (see above, and DEAH) and the four cysteine residues that coordinate the 4Fe-4S cluster indicates an acceptable alignment between the Chl1 model and the XPD template (achieving approximately 21% sequence identity).

Supplemental References

Amberg, D.C., Burke, D.J., and Strathern, J.N. (2005). Methods in yeast genetics (Cold Spring Harbor, New York: Cold Spring Harbor Laboratory Press).

Bates, P.A., Kelley, L.A., MacCallum, R.M., and Sternberg, M.J. (2001). Enhancement of protein modeling by human intervention in applying the automatic programs 3D-JIGSAW and 3D-PSSM. Proteins *Suppl 5*, 39-46.

Bianco, J.N., Poli, J., Saksouk, J., Bacal, J., Silva, M.J., Yoshida, K., Lin, Y.-L., Tourriere, H., Lengronne, A., and Pasero, P. (2012). Analysis of DNA replication profiles in budding yeast and mammalian cells using DNA combing. Methods *57*, 149-157.

Borges, V., Lehane, C., Lopez-Serra, L., Flynn, H., Skehel, M., Rolef Ben-Shahar, T., and Uhlmann, F. (2010). Hos1 deacetylates Smc3 to close the cohesin acetylation cycle. Mol. Cell *39*, 677-688.

Godfrey, M., Kuilman, T., and Uhlmann, F. (2015). Nur1 dephosphorylation confers positive feedback to mitotic exit phosphatase activation in budding yeast. PLoS Genet. *11*, e1004907.

Knop, M., Siegers, K., Pereira, G., Zachariae, W., Winsor, B., Nasmyth, K., and Schiebel, E. (1999). Epitope tagging of yeast genes using a PCR-based strategy: more tags and improved practical routines. Yeast *15*, 963-972.

Lengronne, A., Katou, Y., Mori, S., Yokobayashi, S., Kelly, G.P., Itoh, T., Watanabe, Y., Shirahige, K., and Uhlmann, F. (2004). Cohesin relocation from sites of chromosomal loading to places of convergent transcription. Nature *430*, 573-578.

Lopez-Serra, L., Kelly, G., Patel, H., Stewart, A., and Uhlmann, F. (2014). The Scc2-Scc4 complex acts in sister chromatid cohesion and transcriptional regulation by maintaining nucleosome-free regions. Nat. Genet. *46*, 1147-1151.

Michaelis, C., Ciosk, R., and Nasmyth, K. (1997). Cohesins: Chromosomal proteins that prevent premature separation of sister chromatids. Cell *91*, 35-45.

Nishimura, K., Fukagawa, T., Takisawa, H., Kakimoto, T., and Kanemaki, M. (2009). An auxin-based degron system for the rapid depletion of proteins in nonplant cells. Nat. Methods *6*, 917-922.

Wach, A., Brachat, A., Pöhlmann, R., and Philippsen, P. (1994). New heterologous modules for classical or PCR-based gene disruptions in *Saccharomyces cerevisiae*. Yeast *10*, 1793-1808.
