# Supplementary material for: Ctf4 Links DNA Replication with Sister Chromatid Cohesion Establishment by Recruiting the Chl1 Helicase to the Replisome
Source: Mol Cell. 2016 Aug 4;63(3):371–84. doi: 10.1016/j.molcel.2016.05.036 (PMC4980427; doi:10.1016/j.molcel.2016.05.036)
Supplement: Document S2. Article plus Supplemental Information [file mmc2.pdf]

Molecular Cell

Article

Ctf4 Links DNA Replication with Sister Chromatid Cohesion Establishment by Recruiting the Chl1 Helicase to the Replisome

Graphical Abstract

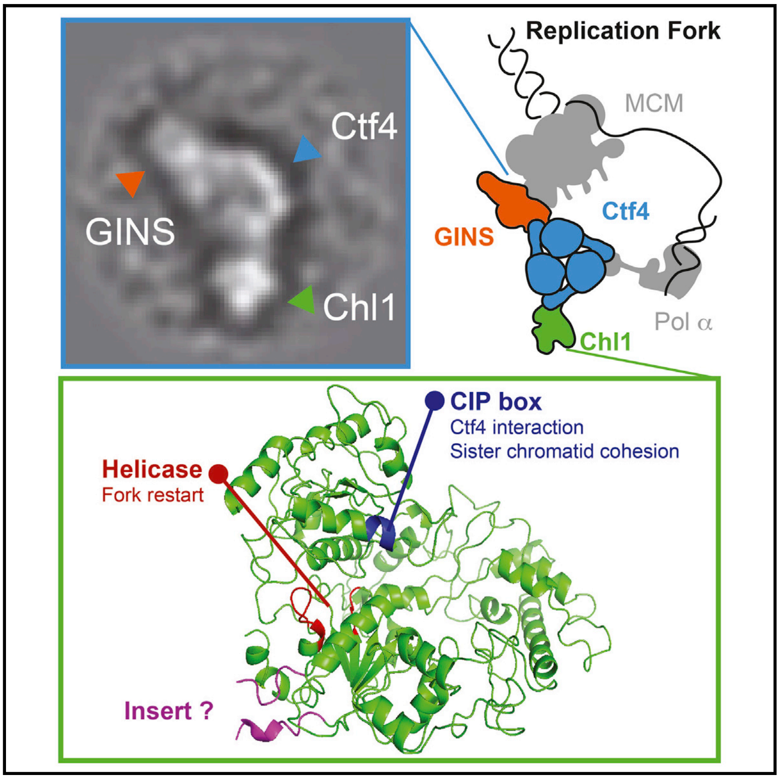

Highlights

- Ctf4 is a replisomal interaction hub that recruits CIP box client proteins
- Chl1 helicase is the Ctf4 client that links replication and cohesion establishment
- Ctf4 interaction, but not helicase activity, conveys Chl1 cohesion function
- Chl1 physically engages with cohesin during cohesion establishment

Authors

Catarina P. Samora, Julie Saksouk, Panchali Goswami, ..., Armelle Lengronne, Alessandro Costa, Frank Uhlmann

Correspondence

frank.uhlmann@crick.ac.uk

In Brief

Eukaryotic DNA replication is accompanied by the establishment of sister chromatid cohesion. Samora et al. show that the central replisome component Ctf4 makes this link by recruiting the Chl1 helicase to the replication fork. Chl1 in turn engages in a direct physical contact with the cohesin complex during cohesion establishment.

Samora et al., 2016, Molecular Cell *63*, 371–384
August 4, 2016 © 2016 The Author(s). Published by Elsevier Inc.
http://dx.doi.org/10.1016/j.molcel.2016.05.036

Molecular Cell
Article

CellPress

Ctf4 Links DNA Replication with Sister Chromatid Cohesion Establishment by Recruiting the Chl1 Helicase to the Replisome

Catarina P. Samora,[1] Julie Saksouk,[2] Panchali Goswami,[3] Ben O. Wade,[4] Martin R. Singleton,[4] Paul A. Bates,[5] Armelle Lengronne,[2] Alessandro Costa,[3] and Frank Uhlmann[1,*]
[1]Chromosome Segregation Laboratory, Francis Crick Institute, London WC2A 3LY, UK
[2]Institute of Human Genetics (IGH), CNRS, 34396 Montpellier, France
[3]Macromolecular Machines Laboratory, Francis Crick Institute, South Mimms EN6 3LD, UK
[4]Structural Biology of Chromosome Segregation Laboratory
[5]Biomolecular Modelling Laboratory
Francis Crick Institute, London WC2A 3LY, UK
*Correspondence: frank.uhlmann@crick.ac.uk
http://dx.doi.org/10.1016/j.molcel.2016.05.036

SUMMARY

DNA replication during S phase is accompanied by establishment of sister chromatid cohesion to ensure faithful chromosome segregation. The Eco1 acetyltransferase, helped by factors including Ctf4 and Chl1, concomitantly acetylates the chromosomal cohesin complex to stabilize its cohesive links. Here we show that Ctf4 recruits the Chl1 helicase to the replisome via a conserved interaction motif that Chl1 shares with GINS and polymerase α. We visualize recruitment by EM analysis of a reconstituted Chl1-Ctf4-GINS assembly. The Chl1 helicase facilitates replication fork progression under conditions of nucleotide depletion, partly independently of Ctf4 interaction. Conversely, Ctf4 interaction, but not helicase activity, is required for Chl1's role in sister chromatid cohesion. A physical interaction between Chl1 and the cohesin complex during S phase suggests that Chl1 contacts cohesin to facilitate its acetylation. Our results reveal how Ctf4 forms a replisomal interaction hub that coordinates replication fork progression and sister chromatid cohesion establishment.

INTRODUCTION

Cohesion between sister chromatids, from the time of DNA replication in S phase until anaphase onset, is crucial for the faithful distribution of genetic information between daughter cells. Sister chromatid cohesion is mediated by the chromosomal cohesin complex, a large ring-shaped protein assembly that topologically encircles DNA (Nasmyth and Haering, 2009; Uhlmann, 2016). Cohesin is loaded onto chromosomes well before S phase, in late G1 phase in budding yeast and even earlier in fission yeast and human cells. Recent progress has been made in our understanding of how DNA enters the cohesin ring, facilitated by a separate cohesin loader complex (Murayama and Uhlmann, 2014). However, the association of cohesin with chromatin in itself is not sufficient to promote sister chromatid cohesion. The establishment of cohesive linkages between sister chromatids is an active process that occurs concomitantly with DNA replication and poses at least two requirements. First, cohesin must entrap not only one, but two strands of DNA. How this is achieved is not yet known. The replication fork might pass through cohesin rings, or cohesin might sequentially embrace two replicated DNAs in the wake of the replication fork, two possibilities that are not mutually exclusive. Second, cohesin rings that hold together sister chromatids must be stabilized on chromosomes, which in budding yeast is achieved through acetylation by the essential, replication fork-associated acetyltransferase Eco1 (Rolef Ben-Shahar et al., 2008; Ünal et al., 2008). Acetylation targets two DNA sensory lysines on the cohesin ATPase that fuel the DNA entry and exit reactions. Their acetylation switches off the dynamic loading and unloading cycle of the cohesin complex to establish enduring sister chromatid cohesion (Chan et al., 2012; Gerlich et al., 2006; Lopez-Serra et al., 2013; Murayama and Uhlmann, 2015). The importance of cohesin stabilization for cohesion establishment is illustrated by the fact that Eco1 becomes dispensable for viability if cohesin is stabilized on chromosomes in an alternative way. This can be achieved by deletion of the non-essential cohesin subunit Wapl, which promotes the dynamic turnover of the complex (Rolef Ben-Shahar et al., 2008).

In addition to the Eco1 acetyltransferase, a number of further "cohesion establishment factors" have been identified using genetic approaches in budding yeast. These are proteins that are not themselves part of the cohesin complex, but that contribute to the establishment of sister chromatid cohesion. Among them are the PCNA clamp loader and unloader RFC^{Ctf18} (Mayer et al., 2001); the three subunits Tof1, Csm3, and Mrc1 of the replication checkpoint complex (Mayer et al., 2004; Xu et al., 2004); the replisome component Ctf4 (Hanna et al., 2001); as well as the Chl1 helicase (Skibbens, 2004). Individually, these factors are not essential. However, inactivation of numerous pairwise

This is an open access article under the CC BY license (http://creativecommons.org/licenses/by/4.0/).

combinations results in additive sister chromatid cohesion defects and lethality (Xu et al., 2007). Cohesin acetylation during S phase is reduced in the absence of any of these cohesion establishment factors, suggesting that they all act at least in part by facilitating the acetylation reaction (Borges et al., 2013). Genetic analysis is consistent with the possibility that RFC^{Ctf18}, Tof1, Csm3, and Mrc1 act in a pathway with Eco1, as their deletion hardly increases the growth defect seen in a strain background lacking Eco1. In contrast, Ctf4 or Chl1 deletion causes a marked synthetic growth defect in the absence of Eco1 (Borges et al., 2013). This suggests that Ctf4 and Chl1 support cohesin acetylation by acting in parallel to Eco1. However, the molecular mechanism by which Ctf4 and Chl1 achieve this is not known.

Ctf4 was originally identified as a DNA polymerase α-interacting factor, important for chromosome stability. It was subsequently shown to be important for sister chromatid cohesion (Hanna et al., 2001; Kouprina et al., 1992; Miles and Formosa, 1992). We now know that Ctf4 is a structural component of the replisome, linking the MCM helicase via GINS to the DNA polymerase α-primase complex (Gambus et al., 2006, 2009; Lengronne et al., 2006; Tanaka et al., 2009a). Recent structural work has shown that Ctf4 is a homotrimer to which GINS and DNA polymerase α bind via a shared interaction motif (Simon et al., 2014). Ctf4 and its role in sister chromatid cohesion are conserved in vertebrates (where Ctf4 is also known as And1; Errico et al., 2009).

Chl1 is encoded by what is probably the first chromosome loss mutant gene to be identified (Haber, 1974). Its cloning suggested that Chl1 is a DNA helicase (Gerring et al., 1990). Biochemical analysis confirmed that the human counterpart of Chl1 (known as ChlR1) is indeed a DNA helicase and that it progresses along single-stranded DNA in the 5′-3′ direction (Farina et al., 2008; Hirota and Lahti, 2000). Consistent with Chl1 function as a DNA helicase, its intact ATPase is required to prevent chromosome loss in both yeast and mice (L Holloway, 2000; Inoue et al., 2007). Chl1 promotes sister chromatid cohesion in yeast and humans (Farina et al., 2008; Mayer et al., 2004; Parish et al., 2006; Skibbens, 2004); however, a formal test of whether the Chl1 ATPase is required for sister chromatid cohesion, or in another way contributes to chromosome stability, is outstanding. Mutations in human ChlR1 are the cause of Warsaw breakage syndrome, a developmental disorder that combines features of defective DNA repair and cohesin function (van der Lelij et al., 2010).

Here, we show that Ctf4 and Chl1 physically interact. Chl1 is recruited to the budding yeast DNA replication fork via a conserved Ctf4-interaction peptide motif that it shares with polymerase α and GINS (which we suggest to be known as "CIP box"). Chl1 and Ctf4 form a multimer that is architecturally reminiscent of the polymerase α-Ctf4-GINS assembly, as observed by single-particle electron microscopy (EM) analysis of a reconstituted protein complex. Ctf4 interaction, but, surprisingly, not helicase activity, is required for Chl1 function in sister chromatid cohesion. This suggests a structural role for Chl1 in cohesion establishment that might involve a direct interaction with cohesin at replication forks. These findings show how Ctf4 forms an interaction hub within the replisome that links replication fork progression to sister chromatid cohesion establishment.

RESULTS

Ctf4 and Chl1 Interact during DNA Replication

Previous studies have placed Ctf4 and Chl1 into one genetically defined cohesion establishment pathway. Deletion of the genes encoding either factor has the same impact on sister chromatid cohesion compared to deleting both (Borges et al., 2013; Xu et al., 2007). Physical complex formation is a familiar mechanism for two proteins to act in one pathway. Therefore, we asked whether Ctf4 and Chl1 interact. To test this, we immunoprecipitated Ctf4 from cells progressing synchronously through the cell cycle following α factor block and release. Chl1 co-precipitated with Ctf4 specifically during S phase, but not before or afterward (Figure 1A). This demonstrates that Ctf4 and Chl1 interact during the time of DNA replication.

To investigate whether Ctf4 interacts with Chl1 at the replication fork, we compared the chromosomal localization pattern of Ctf4 and Chl1 by chromatin immunoprecipitation (ChIP). Synchronized cells were arrested in early S phase by hydroxyurea (HU) treatment, and the position of active replication forks was determined by BrdU incorporation into newly synthesized DNA, followed by ChIP against BrdU (Figure 1B). ChIP against Ctf4 confirmed its localization to replication forks, as previously demonstrated (Gambus et al., 2006; Lengronne et al., 2006). When we performed ChIP against Chl1, we found that it was also enriched in replicating regions. This opens the possibility that Chl1 binds Ctf4 at DNA replication forks. Chl1 was detectable at active replication origins during early S phase in the absence of HU, suggesting that Chl1 associates with the replisome also during undisturbed S phase progression (Figure 1C).

The interaction between Ctf4 and Chl1 is restricted to S phase, so we asked whether cell-cycle regulation of either protein could explain this timing. However, levels and migration pattern during gel electrophoresis of both proteins remained constant throughout the cell cycle (Figures 1A and S1A, available online). We furthermore analyzed the subcellular localization of Ctf4 and Chl1 by indirect immunofluorescence. Ctf4 showed nuclear accumulation, while Chl1 displayed a diffuse staining pattern throughout the nucleus and cytoplasm, during all stages of the cell cycle (Figure S1B). Thus, neither protein is subjected to overt cell-cycle regulation. Instead, the interaction between Ctf4 and Chl1 might depend on replisome assembly, when concerted interactions between numerous proteins that are not individually detectable at other times (Gambus et al., 2009) come together to build the replication fork machinery.

Chl1 Recruitment through a Conserved CIP Box Motif

The GINS component Sld5 and the polymerase α large subunit Pol1 share a conserved Ctf4 binding motif that docks onto an exposed helical extension found on each Ctf4 protomer (Simon et al., 2014). By sequence gazing, we detected a match to this motif, "DDIL," in Chl1 (Figure 2A). To test whether this motif mediates Chl1 interaction with Ctf4, we mutated two residues within this motif to alanines (Chl1^{DAIA}). The crystal structure of the corresponding Sld5 peptide bound to Ctf4 shows these residues engaged in prominent contacts (Simon et al., 2014). We generated a budding yeast strain in which *CHL1* was replaced with *chl1^{DAIA}* at the endogenous gene locus, then visualized Chl1

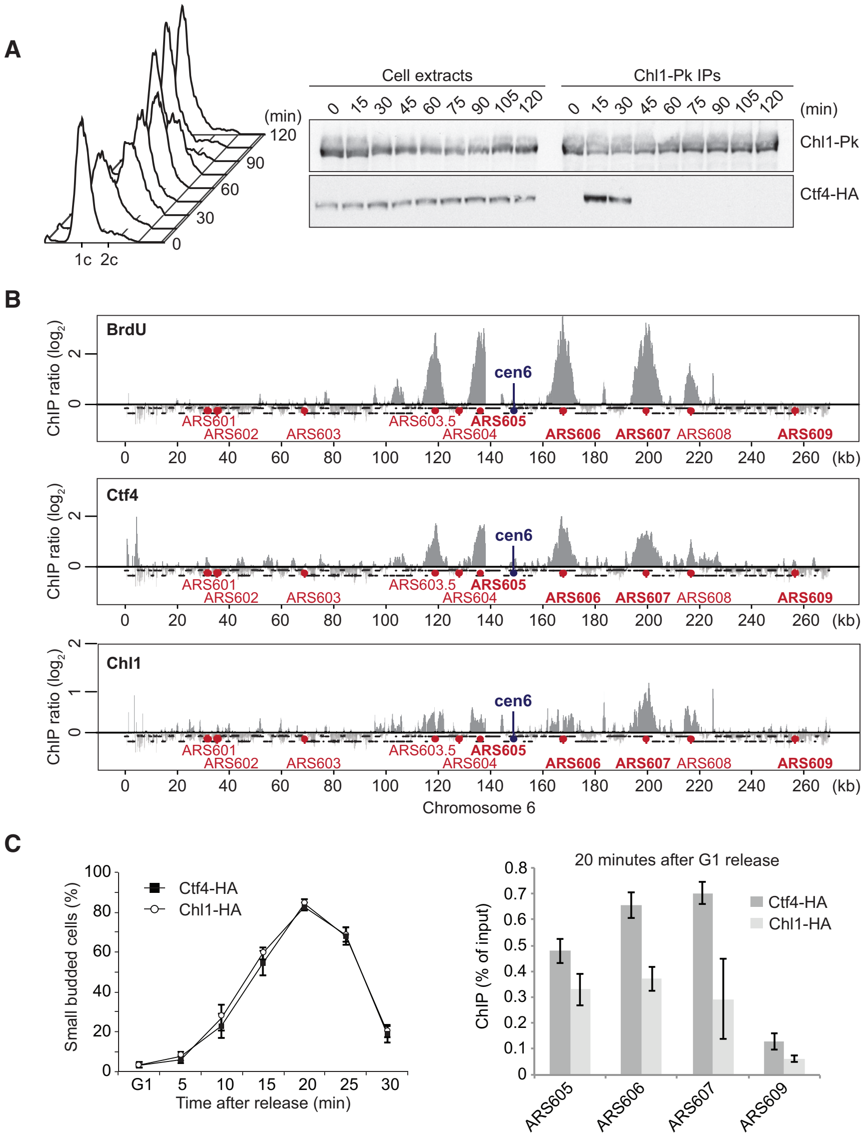

Figure 1. Ctf4 and Chl1 Interact and Co-localize during S Phase

(A) Ctf4 and Chl1 interact during S phase. Cell extracts were prepared from aliquots of a culture passing through a synchronous cell cycle at the indicated times. Pk epitope-tagged Chl1 was immunoprecipitated, and co-precipitation of Ctf4 was analyzed by SDS-PAGE followed by immunoblotting. Cell-cycle progression was monitored by FACS analysis of DNA content.

(B) Chl1 co-localizes with Ctf4 at HU-arrested replication forks. Cells were synchronized in G1 and released into BrdU- and HU-containing medium for arrest in early S phase. ChIP analysis against BrdU and epitope-tagged Ctf4 and Chl1 was performed. Chromatin immunoprecipitates were hybridized to Affymetrix GeneChip *S. cerevisiae* tiling 1.0 R arrays. Signal intensities, relative to a whole-genome DNA sample, are shown along chromosome 6. Replication origins are indicated; those chosen for subsequent quantitative analysis are highlighted in bold. The microarray data are available from the GEO database under the accession number GEO: GSE80007.

(C) Chl1 localizes to replicating regions in early S phase under unchallenged conditions. Chl1 ChIP was performed 20 min after synchronous release from α factor block when cells reached early S phase, as seen by a large fraction of cells with small buds (less than half the diameter of the mother cell). Enrichment close to three early (ARS605, 606, and 607) and a late firing (ARS609) replication origin were compared. Ctf4 ChIP was performed for comparison. The means and SE of three independent experiments are shown.

See also Figure S1 for an analysis of Ctf4 and Chl1 protein levels and subcellular localization during the cell cycle.

interaction with Ctf4 using cells arrested in early S phase (Figure 2B). Ctf4 interaction was lost in the case of Chl1^{DAIA}, even though Chl1^{DAIA} was stably expressed at levels equal to wild-type Chl1. As an additional control, a Chl1 variant carrying a mutation in the helicase active site (Chl1^{K48R}) was included in the analysis, which retained association with Ctf4. This suggests that a conserved CIP box mediates the interaction of Chl1 with Ctf4.

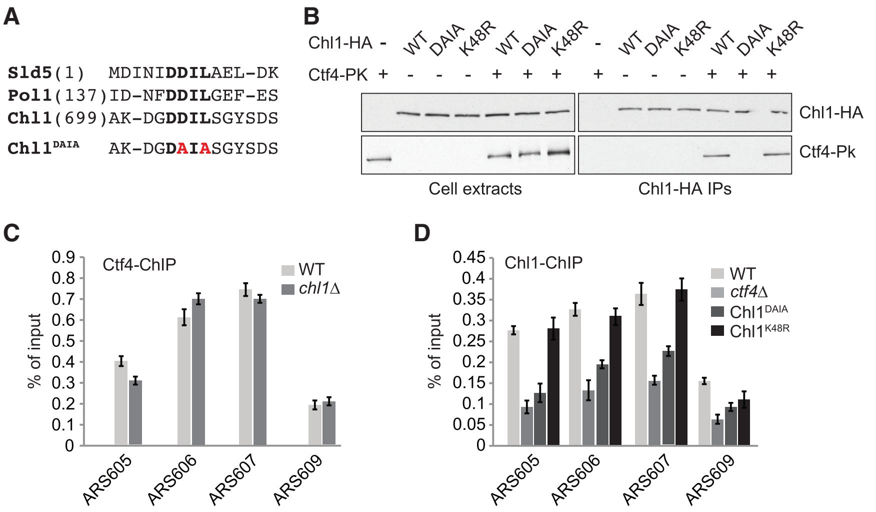

Figure 2. Ctf4 Recruits Chl1 to the Replisome via a Conserved CIP Box

(A) Sequence alignment of the Ctf4-interacting peptide (CIP) boxes in *S. cerevisiae* Sld5 and Pol1 and a matching sequence in Chl1. Invariant residues are highlighted in bold. CIP box mutations to generate Chl1^{DAIA} are indicated in red.

(B) CIP box-dependent interaction of Chl1 with Ctf4. Cell extracts were prepared from cultures of the indicated strains, synchronized, and arrested in early S phase by HU treatment. Chl1-HA was immunoprecipitated, and co-precipitation of Ctf4 detected by immunoblotting.

(C) Ctf4 localizes to the replication fork independently of Chl1. Ctf4 ChIP was performed in synchronized cells arrested in early S phase by HU treatment in the presence or absence of Chl1. Enrichment at three early firing origins, active in HU (ARS605, 606, and 607), and a late-firing, inactive origin (ARS 609) was compared by quantitative real-time PCR. The means and SE of three independent experiments are shown.

(D) Chl1 is recruited to replication forks by CIP box-dependent binding to Ctf4. As in (C), but Chl1 ChIP was performed in the indicated strains.

We next studied the importance of the Ctf4-Chl1 interaction for the recruitment of both proteins to the replication fork. We used ChIP followed by quantitative real-time PCR (ChIP quantitative real-time PCR) from cells arrested in early S phase by HU treatment to assess protein binding near three early firing, active replication origins. A region close to a late-firing origin, inactive in HU-treated cells, served as a control. Ctf4 was readily detected at the three active origins, and the level of association was unaltered in cells lacking Chl1 (Figure 2C). Thus, Ctf4 association with the replisome is independent of Chl1. Conversely, Chl1 binding to the same origins was greatly reduced in the absence of Ctf4, suggesting that Ctf4 recruits Chl1 (Figure 2D). Chl1^{DAIA} binding to origins, in the presence of Ctf4, was reduced compared to wild-type Chl1, demonstrating a role of the CIP box in recruiting Chl1 to the replisome. A lower level of Chl1^{DAIA}, which remained detectable, suggests that Chl1 makes additional contacts with Ctf4 or other components of the replisome. Chl1 helicase activity was not required for recruitment to the replisome, as Chl1^{K48R} was detected at levels equal to the wild-type protein.

Visualization of the Ctf4-Chl1 Interaction

Ctf4 forms a homotrimer within the replisome, offering three docking sites to client proteins (Simon et al., 2014). Two of these sites are used to bridge GINS and the polymerase α-primase complex, opening the possibility that the third protomer simultaneously recruits Chl1. To obtain insight into the temporal regulation of Ctf4 interaction with three binding partners, we again immunoprecipitated Ctf4 at intervals from cells that progressed synchronously through the cell cycle and probed for co-precipitation of the three interactors. This analysis revealed a constitutive interaction of Ctf4 with the GINS subunit Psf2, detectable throughout the cell cycle, consistent with a previous report (Gambus et al., 2009; Figure 3A). Chl1 binding became detectable at the onset of S phase, also when the polymerase α subunit Pol1 associated with Ctf4. Chl1 association was lost 30 min later, when most DNA replication was complete. In contrast, Pol1 remained detectable with Ctf4 for another 15 min, before its association was also lost. These findings suggest that Ctf4 interacts stably with GINS, while its interactions with Chl1 and the polymerase α-primase complex occur with overlapping yet distinct temporal regulation.

To provide visual evidence that a replisome-incorporated Ctf4 trimer can associate with Chl1, we recombinantly expressed and purified Chl1, the hetero-tetrameric GINS complex, and the Ctf4 C-terminal trimerization core that contains the CIP box acceptor. Following an established protocol (Simon et al., 2014), we reconstituted various permutations of Ctf4-client protein assemblies for characterization by two-dimensional single-particle EM (Figure 3B). As previously reported, the Ctf4 core forms a trimeric disk. We confirmed that, mixed in equimolar amounts, one, two, or three GINS assemblies bind to one Ctf4 complex and form a rod-like feature that radially departs from the homo-trimerization core. Performing the same reconstitution experiment with Ctf4 and Chl1 yields a partially occupied Ctf4 trimer, with one or two (but not three) hook-shaped Chl1 molecules binding to the Ctf4 disk (Figures 3C and S2A). This is compatible with the notion that Chl1 is a transient Ctf4 interactor in vivo, compared to constitutively bound GINS. A reconstituted Ctf4 hetero-complex containing both client proteins reveals a Ctf4 assembly concurrently associated with both rod-shaped GINS and hook-shaped Chl1 molecules (Figures 3C and S2B). This shows that Ctf4 can physically bridge a replisome component with the Chl1 helicase.

The Ctf4-Chl1 Interaction Promotes Sister Chromatid Cohesion

We next addressed whether Chl1 interacts with Ctf4 to establish sister chromatid cohesion. For this, we took advantage of the Chl1^{DAIA} mutant, which is expressed at levels equal to wild-type Chl1, but whose Ctf4 interaction and association with

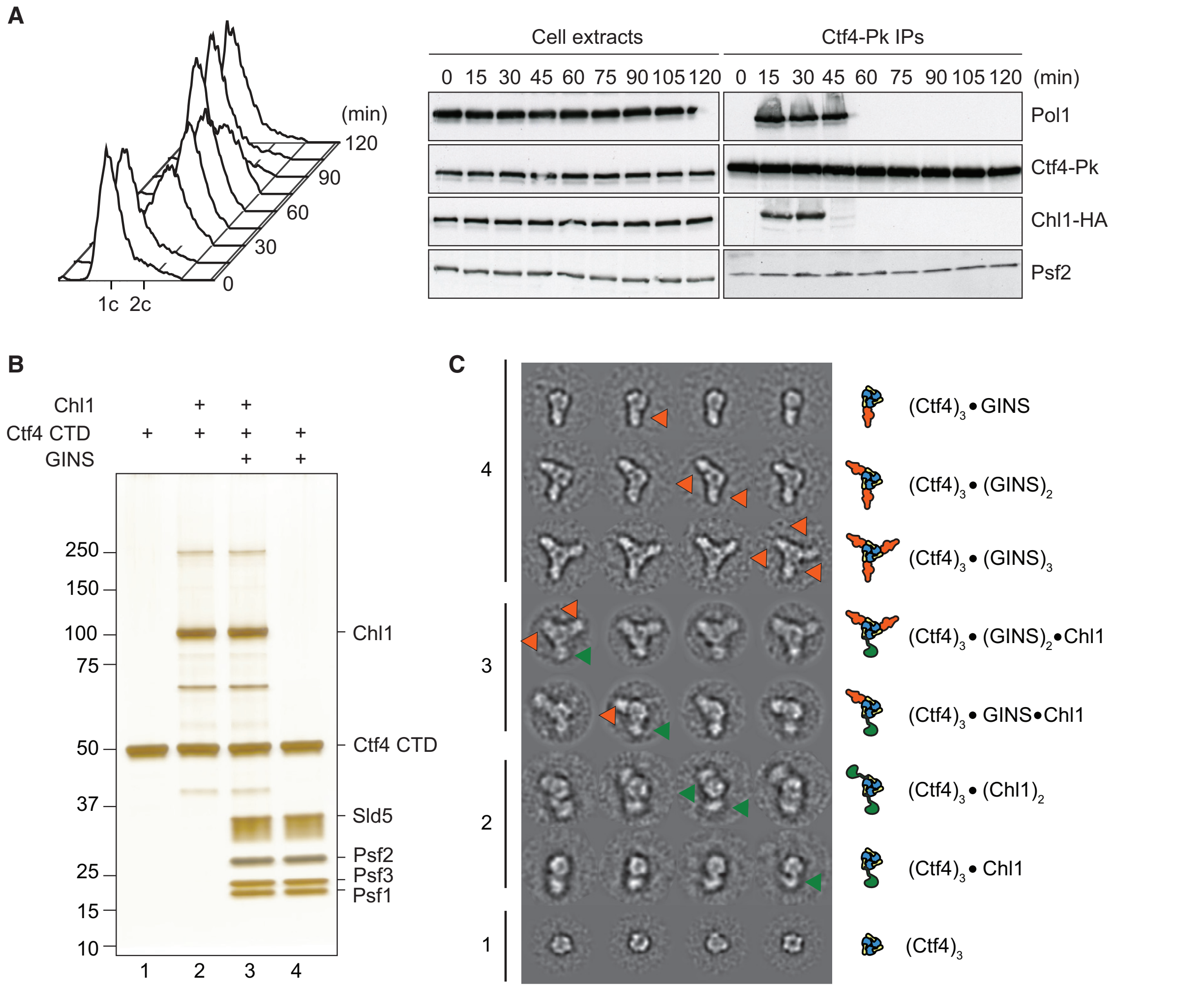

Figure 3. Ctf4 Is a Recruitment Platform at the Replication Fork

(A) Cell-cycle pattern of Ctf4 interactions. Cell extracts were prepared at the indicated times from a culture progressing synchronously through the cell cycle. Ctf4 was immunoprecipitated, and co-precipitation of the GINS subunit Psf2, the polymerase α subunit Pol1, and Chl1 was analyzed by immunoblotting. Cell-cycle progression was monitored by FACS analysis of DNA content.

(B) Silver-stained gel of the reconstituted Ctf4 client protein assemblies. Ctf4 was prepared in isolation or bound by Chl1, GINS, or both.

(C) Two-dimensional averages of Chl1-client protein assemblies imaged by negative-stain EM. Chl1 is highlighted by green arrowheads and in the same color in the diagrams. GINS is orange and Ctf4 is blue/yellow. Box size is 441 Å × 441 Å.

See also Figure S2 for more reference-free class averages of the protein assemblies.

replication forks are compromised. To assess sister chromatid cohesion, we monitored the GFP-marked *URA3* locus. Cells were synchronized by α factor block and release and were then arrested in mitosis by nocodazole treatment. *ctf4Δ* and *chl1Δ* strains were included in this experiment, which displayed expected cohesion defects (Hanna et al., 2001; Skibbens, 2004) (Figure 4A). The *chl1^{DAIA}* mutant cells also showed a marked cohesion defect, albeit not to the full extent seen in *ctf4Δ* or *chl1Δ* cells. This demonstrates the importance of the Ctf4-Chl1 interaction for sister chromatid cohesion. Residual Chl1^{DAIA} association with the replisome might account for the less severe phenotype. The importance of Ctf4 interaction for Chl1 function was underscored in an experiment in which we overexpressed Chl1 with the aim of providing Chl1 function independently of Ctf4. Expression under control of the galactose-inducible *GAL1* promoter led to greatly increased Chl1 levels. This fully restored sister chromatid cohesion in a *chl1Δ* background, but not in the absence of Ctf4 (Figure S3A). Thus, the ability to interact with Ctf4 is crucial for Chl1's function in sister chromatid cohesion.

Chl1 is thought to function as a helicase in chromosome stability (L Holloway, 2000; Inoue et al., 2007). We therefore asked whether helicase function is required for sister chromatid cohesion. Previous studies have replaced a conserved lysine in the ATP binding site with arginine, which in the case of the human ChlR1 enzyme abolishes helicase activity in vitro (Farina et al., 2008; Hirota and Lahti, 2000). To our surprise, the Chl1^{K48R} mutation in budding yeast Chl1 did not compromise sister

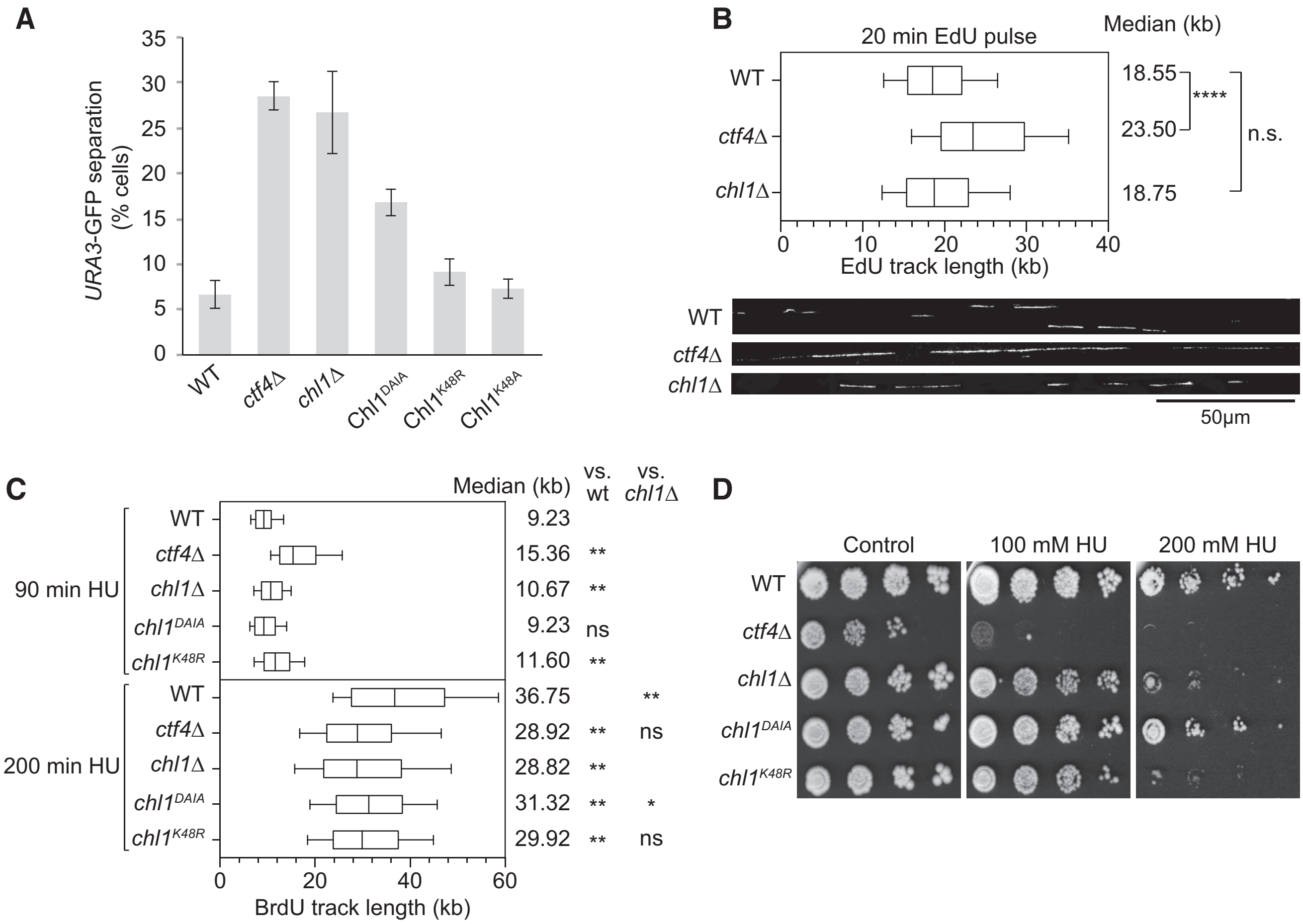

Figure 4. Separable Chl1 Functions in Sister Chromatid Cohesion and Replication Fork Integrity

(A) Ctf4 interaction, but not Chl1 helicase activity, contributes to sister chromatid cohesion. Cells of the indicated genotypes were synchronized and arrested in a nocodazole-imposed mitotic arrest. Sister chromatid cohesion was assessed at the GFP-marked *URA3* locus. The means and SE ofthree independent experiments are shown.

See also Figure S3, showing that Chl1 overexpression cannot provide Chl1 function without Ctf4 and that ATPase activity is abrogated in recombinant Chl1^{K48R} and Chl1^{K48A} proteins.

(B) Chl1 makes no detectable contribution to replication fork progression under unchallenged conditions. Fork speed, measured after pulse incorporation of 5-ethynyl-2'-deoxyuridine (EdU) and DNA combing, was analyzed in the indicated strains. Examples of the EdU tracks in each of the strains are shown. The graph depicts the distribution of EdU track lengths. Box and whiskers indicate 25–75 and 10–90 percentiles, respectively. Medians are shown by a line and are listed. Asterisks indicate the significance of the statistical test (**$p < 0.0001$, *$p = 0.0021$; n.s., not significant; Mann-Whitney unpaired non-parametric t test).

(C) Chl1 and Ctf4 are required for replication fork progression under conditions of dNTP depletion. As in (B), but cells of the indicated genotypes were synchronized by α factor block and release into medium containing BrdU and 200 mM HU. BrdU track lengths were measured at 90 and 200 min after release.

(D) Chl1 helicase activity is required for HU-resistant cell growth. Ten-fold serial dilutions of strains of the indicated genotypes were spotted on YPD agar containing the indicated HU concentrations.

chromatid cohesion (Figure 4A). Chl1^{K48R} might have retained residual helicase function in vivo, so we introduced a more severe K48A mutation. Cells expressing Chl1^{K48A} as the sole source of Chl1 again did not display a noticeable sister chromatid cohesion defect. To confirm that the mutant Chl1 proteins were deficient in helicase activity, we purified budding yeast Chl1, Chl1^{K48R}, and Chl1^{K48A} following overexpression in insect cells (Figures S3B and S3C). Biochemical analysis confirmed that both the arginine and alanine substitutions obliterated the ability of Chl1 to hydrolyze ATP in the presence of single-stranded DNA. These results confirm that Chl1^{K48R} and Chl1^{K48A} are helicase deficient and suggest that helicase activity is not required for Chl1 to fulfill its role in sister chromatid cohesion.

Separable Chl1 Functions in Sister Chromatid Cohesion and Replication Fork Progression

Surprised by the finding that Chl1 helicase activity is dispensable for sister chromatid cohesion, we investigated whether the Chl1 helicase contributes to replication fork progression. The main replicative MCM helicase moves in 3′-5′ direction along the leading strand. As a 5′-3′ helicase, Chl1 could act in parallel, promoting DNA unwinding on the lagging strand. To investigate whether

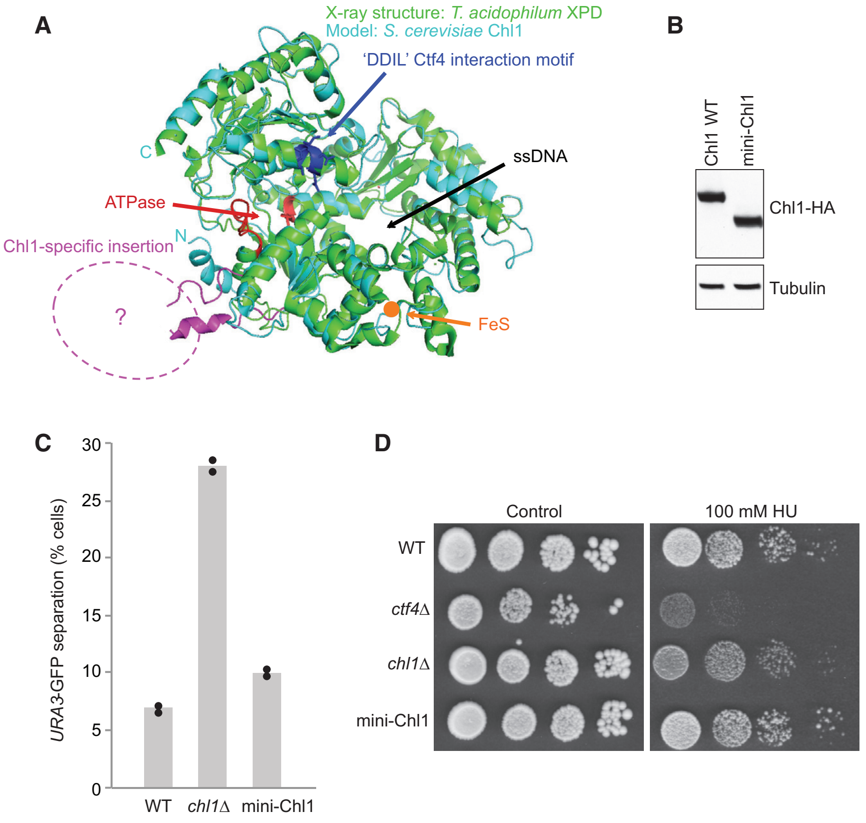

Figure 5. A Chl1-Specific Insert in XPD Family Helicases

(A) A structural model of *S. cerevisiae* Chl1 based on the *T. acidophilum* XPD helicase crystal structure (Wolski et al., 2008). Regions containing the ATPase motifs are highlighted in red, the position of the 4Fe4S cluster is indicated in orange, while the CIP box sequence is shown in dark blue. The position of the Chl1-specific insertion is indicated in pink.

See also Figure S4 for a sequence alignment of Chl1 and XPD, showing the Chl1-specific insert and how it was removed and the gap sealed to create mini-Chl1.

(B) Expression of mini-Chl1, lacking the Chl1-specific insertion. Whole-cell extracts, prepared from asynchronous cultures, were separated by SDS-PAGE. Chl1 and mini-Chl1 were detected by immunoblotting against their C-terminal HA epitope tags. Tubulin served as a loading control.

(C) mini-Chl1 promotes sister chromatid cohesion. Sister chromatid cohesion in the indicated strains was analyzed as in Figure 4A. The results and means from two independent experiments are shown.

(D) mini-Chl1 promotes HU-resistant cell growth. Ten-fold serial dilutions of the indicated strains were spotted on YPD agar without or containing 100 mM HU.

Chl1 contributes to DNA replication, we monitored replication fork progression in an unchallenged, exponentially growing cell population during a 20 min pulse with the thymidine analog 5-ethynyl-2′-deoxyuridine (EdU). EdU tracks were then visualized along individual DNA fibers that were stretched by DNA combing, and the track lengths were measured. There was no significant difference in fork progression rates between *chl1Δ* and wild-type cells (Figure 4B). Therefore, Chl1 does not make a detectable contribution to DNA replication under unchallenged conditions. Consistent with previous observations, replication forks were significantly accelerated in *ctf4Δ* cells, probably due to the increased deoxynucleotide (dNTP) pools in these cells (Poli et al., 2012).

We next analyzed fork progression following release from α factor block into medium containing HU, which slows down replication fork progression due to dNTP depletion, and BrdU. At early times after release (Figure 4C; 90 min), while existing nucleotide pools are used up, track lengths showed a similar pattern to what was observed in unchallenged cells. This situation changed after longer times in the arrest (200 min). Wild-type replication forks continued to progress slowly. Notably, forks progressed even slower in *ctf4Δ*, *chl1Δ*, and *chl1*^{K48R} cells, and also *chl1*^{DAIA} cells, albeit to a lesser degree in the latter. This suggests that both Ctf4 and Chl1 are required to maintain fork progression under conditions of replication stress. The Chl1 helicase might do so by aiding replication fork restart following repeated rounds of stalling due to the low dNTP levels. Observations that support these conclusions have recently been made in human cells lacking ChlR1 (Calì et al., 2016).

Consistent with a role of Ctf4 and Chl1 in response to replication stress, strains lacking these proteins showed growth retardation on HU-containing medium. This was very pronounced in the case of *ctf4Δ* cells, while growth of *chl1Δ* cells was compromised less severely (Figure 4D). This suggests that Ctf4 plays a role in response to HU in addition to recruiting Chl1. This might include its interaction with the polymerase α-primase or other factors, e.g., Mms22 (Gambus et al., 2009; Mimura et al., 2010). Chl1 function in response to HU depended less on its interaction with Ctf4, as Chl1^{DAIA} conferred HU resistance equal to wild-type Chl1. Helicase activity in turn was required, as a *chl1*^{K48R} strain was equally HU sensitive as a *chl1Δ* strain. Taken together, this ascribes two separable functions to Chl1. Its helicase facilitates DNA replication under conditions of dNTP depletion. Cohesion establishment, in contrast, requires that Chl1 binds to Ctf4, but does not involve its helicase activity.

A Chl1-Specific Insertion in the XPD Helicase Family

If not as a helicase, how does Chl1 facilitate cohesion establishment? Chl1 is a member of the XPD family of DNA helicases with roles in genome stability, characterized by a distinctive iron sulfur cluster (White, 2009). Chl1 is singled out within the family by an ~20 kDa domain insertion between the Walker A and B motifs of the ATPase active site (Figure S4). We therefore wondered whether this Chl1-specific insert mediates Chl1's role in sister chromatid cohesion. To address this, we constructed a variant Chl1 lacking this insertion. An internal deletion of 179 amino acids was designed, guided by a structural alignment of budding yeast Chl1 with the crystal structure of *T. acidophilum* XPD (Wolski et al., 2008). A 12-amino-acid peptide linker to seal the deletion was created based on the XPD sequence (Figures 5A

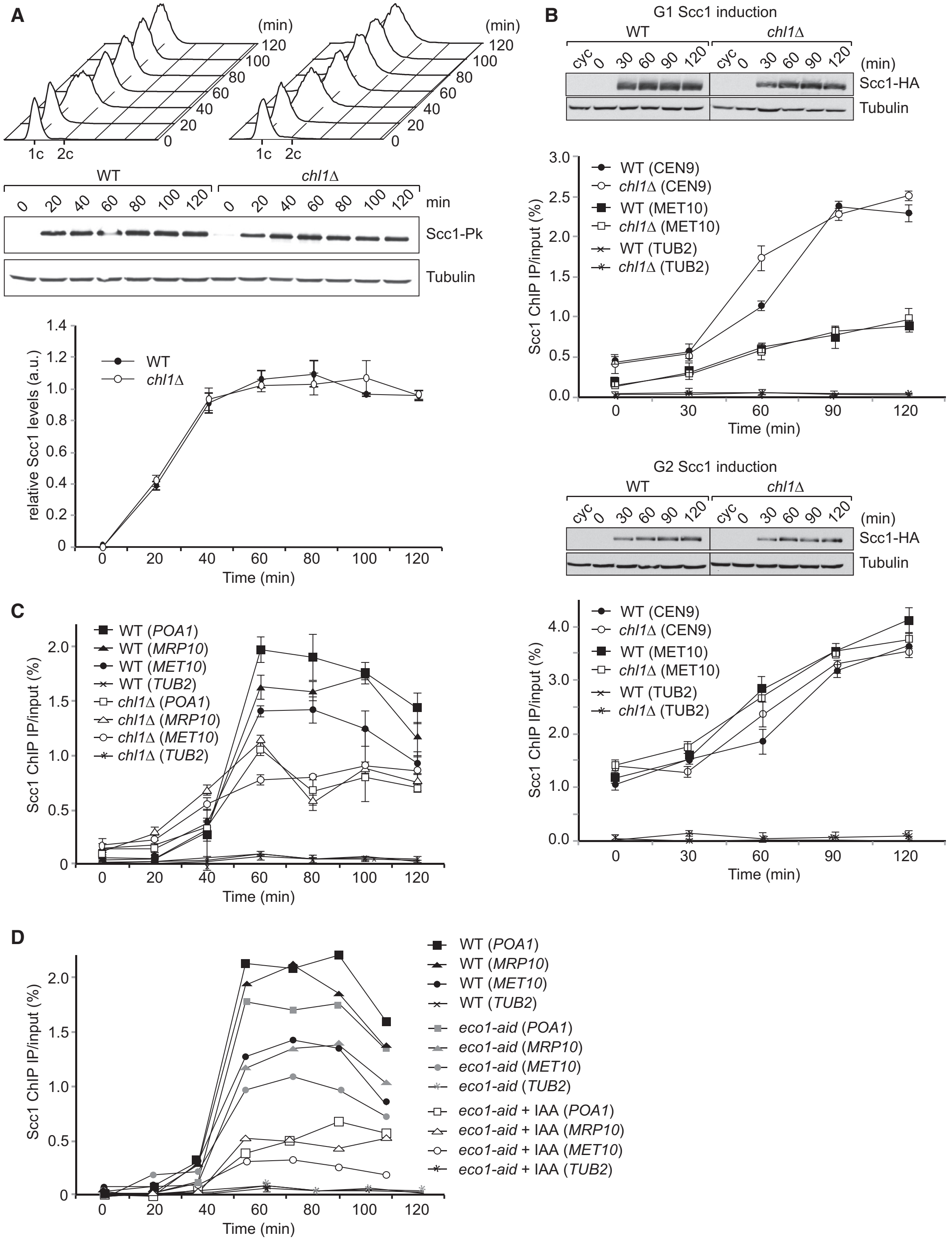

Figure 6. Acetylation Augments Chromosomal Cohesin Levels

(A) Cohesin levels in the absence of Chl1. Scc1 levels were analyzed by quantitative immunoblotting in cultures passing synchronously from α factor block into nocodazole-imposed mitotic arrest. α-tubulin served as loading control for normalization. Cell-cycle progression was monitored by FACS analysis of DNA content. Means and SD from three independent experiments are shown.

(legend continued on next page)

and S4). The resultant "*mini-CHL1*" gene was used to replace endogenous budding yeast *CHL1*. Mini-Chl1 was expressed as a stable protein at levels comparable to full-length Chl1 (Figure 5B).

We now tested the ability of mini-Chl1 to support the establishment of sister chromatid cohesion. Cells expressing mini-Chl1 showed a small increase of cells with premature sister chromatid separation compared to wild-type cells, but the defect was far less than that in *chl1Δ* cells (Figure 5C). This suggests that the Chl1-specific insert makes only a small contribution to sister chromatid cohesion. The main contribution to sister chromatid cohesion must be made by another part of Chl1. We also found that mini-Chl1 confers HU resistance to a degree indistinguishable from wild-type Chl1 (Figure 5D). Thus, a role for the Chl1-specific insert remains to be assigned in future studies.

Chl1 and Chromosomal Cohesin Levels

Previous studies reported that chromosomal cohesin levels are reduced to about half in cells lacking Chl1 (Borges et al., 2013; Laha et al., 2011; Rudra and Skibbens, 2013). This reduction by itself is unlikely to explain the cohesion defect in *chl1Δ* cells, as even greater reduction of cohesin levels is inconsequential in the presence of Chl1 (Heidinger-Pauli et al., 2010). However, to know the reason for reduced cohesin levels might help to understand how Chl1 works.

We first established whether total cellular cohesin levels were altered in the absence of Chl1, which could explain reduced chromosomal levels. Quantitative immunoblotting from cultures progressing synchronously through the cell cycle revealed similar Scc1 subunit levels between wild-type and *chl1Δ* cells (Figure 6A). Scc1 was used as a representative cohesin subunit, as its stability depends on Smc1 and Smc3 (Tóth et al., 1999). Furthermore, immunoprecipitation of Scc1 from wild-type and *chl1Δ* cells confirmed equal abundance of the cohesin complex (Figure S5A). Therefore, reduced cohesin levels on chromosomes in *chl1Δ* cells are unlikely due to reduced availability of the cohesin complex.

We next asked whether cohesin loading onto chromosomes is compromised in the absence of Chl1. We arrested cells in either G1 phase or mitosis by α factor or nocodazole treatment, respectively. Once arrested, ectopic expression of Scc1 was induced under control of the galactose-inducible *GAL1* promoter. In G1, separase-resistant Scc1R180,268E was expressed to prevent cleavage by separase that is active at this time (Uhlmann et al., 1999). We then compared accumulation of Scc1 in whole-cell extracts by immunoblotting and on chromosomes by ChIP (Figure 6B). Scc1 accumulated with equal kinetics in wild-type and *chl1Δ* cells, first in cell extracts and then on chromosomes. Thus, Chl1 made no measurable contribution to ectopic cohesin loading. The same was observed in cells lacking Ctf4 (Figures S6A and S6B).

It has been suggested that Chl1 promotes cohesin loading specifically during S phase (Rudra and Skibbens, 2013), an effect that might have been missed in the above experiment. We therefore analyzed chromosomal cohesin levels as wild-type and *chl1Δ* cells passed synchronously through the cell cycle. Chromosomal cohesin initially accumulated at comparable rates in both *chl1Δ* and wild-type cells. Around the time of S phase, the cohesin ChIP signal in wild-type cells displayed a sharp increase. This increase depended on Chl1 and was not observed in its absence (Figure 6C). Similarly, it depended on Ctf4 (Figure S6C). Because cohesin acetylation occurs around this time, and because cohesin acetylation is compromised in the absence of Chl1 (Borges et al., 2013), we investigated whether acetylation was responsible for increased chromosomal cohesin. We performed a similar ChIP time course experiment following auxin-induced degradation of the cohesin acetyltransferase Eco1 (Figure 6D). This analysis revealed that acetylation is required to augment chromosomal cohesin levels during S phase. This can be rationalized if we consider that acetylation stabilizes cohesin on chromosomes (Chan et al., 2012; Lopez-Serra et al., 2013), which is expected to shift the equilibrium between free and chromosomal cohesin toward the chromosome-bound state. Biochemical fractionation similarly indicated that Eco1 augments chromosomal cohesin levels (Tóth et al., 1999). This opens the possibility that Chl1 impacts chromosomal cohesin levels by facilitating cohesin acetylation.

Chl1 Promotes Efficient Cohesin Usage during Cohesion Establishment

To address how Chl1 promotes cohesin acetylation, we asked whether sister chromatid cohesion in *chl1Δ* cells could be

See also Figure S5 for a control that integrity of the cohesin complex is unaffected in cells lacking Chl1, and Figure S6A that Scc1 levels are unaffected in the absence of Ctf4.

(B) Ectopic cohesin loading onto chromosomes is unaffected by Chl1. Scc1 was expressed under control of the *GAL1* promoter in G1 or in mitotically arrested cells. Ectopic Scc1 levels were monitored by immunoblotting, and its loading onto chromosomes was quantified by ChIP quantitative real-time PCR at a centromeric (*CEN9*) and a chromosome arm (*MET10*) cohesin-binding site; a negative site (*TUB2*) was included as control. All immunoblot samples were run on the same gel, separated by additional lanes where indicated.

See also Figure S5B for confirmation of cell synchrony by FACS analysis of DNA content and Figure S6B, showing that cohesin loading is unaffected in the absence of Ctf4.

(C) Chl1 augments chromosomal cohesin levels during S phase. Cohesin association with three chromosome arm regions (*POA1*, *MRP10*, and *MET10*) and a negative control site (*TUB2*) was quantified in wild-type and *chl1Δ* cells passing synchronously from α factor block into nocodazole-imposed mitotic arrest. Means and SD from three independent experiments are shown.

See also Figure S5C for confirmation of cell synchrony by FACS analysis of DNA content and Figure S6C, showing that Ctf4 is required for increased cohesin binding to chromosome during S phase.

(D) Cohesin acetylation promotes increased cohesin association with chromosomes during S phase. As in (C), but chromosomal cohesin levels were compared between wild-type and *MET3-eco1-aid* cells that were synchronized by α factor addition under permissive (−methionine) or restrictive (+ methionine, + auxin indole acetic acid [IAA]) conditions.

See also Figure S5D for confirmation of cell synchrony by FACS analysis of DNA content.

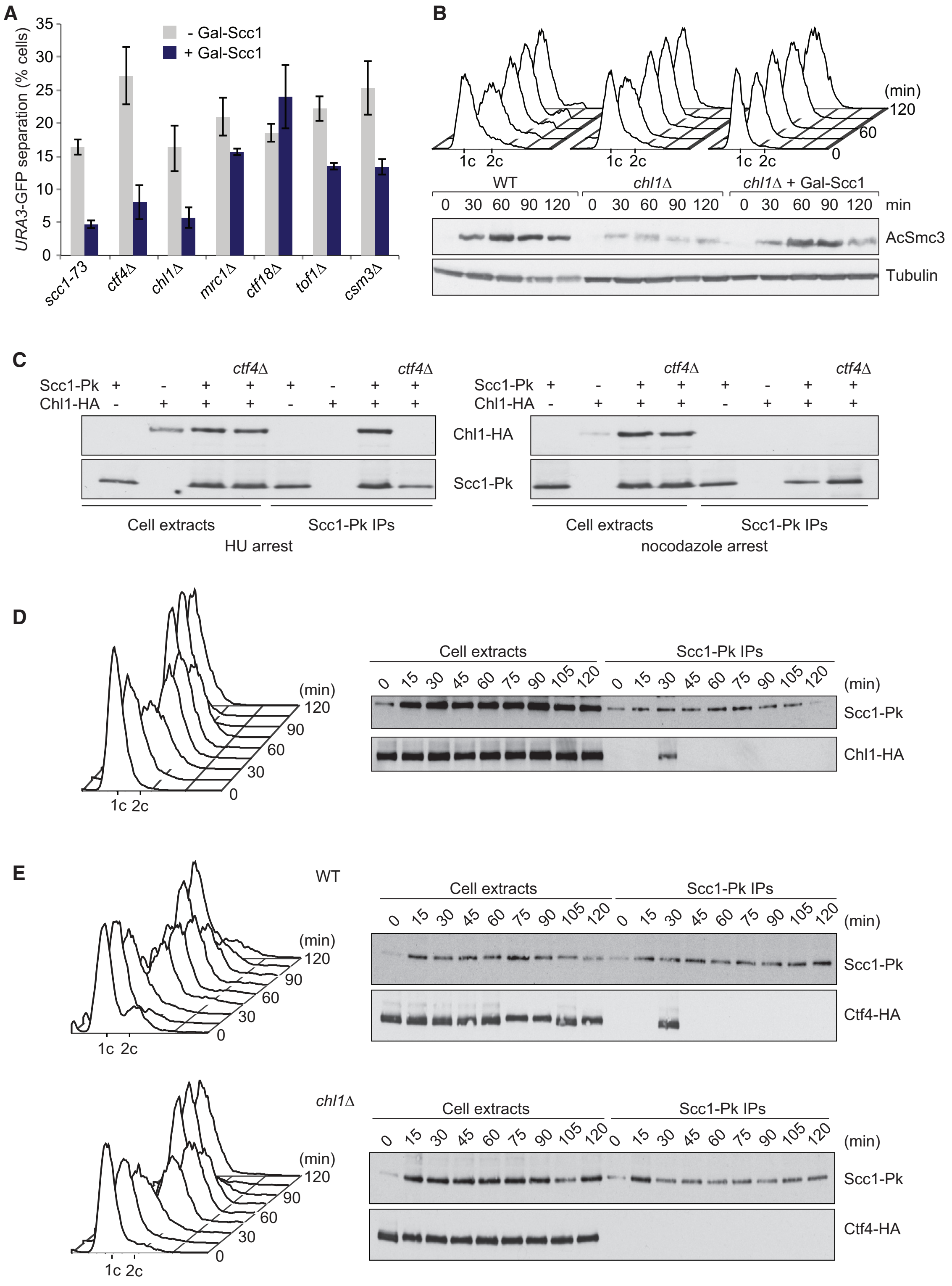

Figure 7. Chl1 Engages Cohesin during S Phase

(A) Scc1 overexpression rescues sister chromatid cohesion in cells lacking Chl1 or Ctf4. Sister chromatid cohesion was assessed in the indicated strains as in Figure 4A. The strains were grown in the absence or presence of galactose to induce ectopic Scc1 expression under control of the *GAL1* promoter. Means and SE of three independent experiments are shown.

(legend continued on next page)

improved by increased cohesin levels. Scc1 overexpression almost completely rescued sister chromatid cohesion in *chl1Δ*, as well as in *ctf4Δ* cells (Figure 7A). Similar cohesin overexpression did not rescue (or only partially rescued) the cohesion defects seen in other cohesion establishment mutants, *ctf18Δ*, *mrc1Δ*, *tof1Δ*, and *csm3Δ*. Thus, a bigger cohesin pool increases the chances of successful cohesion establishment in the absence of Ctf4 or Chl1, but not, or less so, in the absence of other cohesion establishment factors.

As another readout for cohesion establishment, we analyzed cohesin acetylation, which is greatly reduced in *chl1Δ* cells. Scc1 overexpression rescued acetylation levels to close to those seen in wild-type cells (Figure 7B). This suggests that cohesin is inefficiently used as an acetylation substrate in the absence of Chl1. In other words, Chl1 promotes the efficient use of cohesin for acetylation and thus cohesion establishment.

Chl1 Engages with Cohesin during S Phase

It has been reported that human ChlR1 and cohesin co-precipitate from crude cell extracts (Parish et al., 2006; Yoshizawa-Sugata and Masai, 2009). We therefore investigated this interaction. Co-immunoprecipitation experiments revealed that the two proteins interacted in a cell-cycle-dependent manner (Figure 7C). The interaction was detected in cells synchronized in S phase by HU treatment, but not if cells were arrested in mitosis using nocodazole. Furthermore, Chl1 must be part of the replication fork to interact with cohesin, as no interaction was observed in the absence of Ctf4 (Figure 7C), or in case of $Chl1^{DAIA}$ (Figure S7A). The cohesin-Chl1 interaction was resistant to benzonase treatment and is therefore likely protein rather than DNA mediated. The interaction was also detected in cells passing synchronously through the cell cycle during a short period in S phase (Figure 7D). Additional evidence that the cohesin-Chl1 interaction is restricted to the replisome came from ChIP analyses. Chl1 localizes to sites of DNA replication, but did not become enriched at cohesin-binding sites during or after S phase (Figure S7B). Furthermore, Chl1 associates with the replisome independently of cohesin (Figure S7C). Thus, replisome-bound Chl1 transiently engages with cohesin, probably as the replication fork moves along chromosomes and encounters cohesin-binding sites.

A cohesin-Chl1 interaction at the replication fork could reflect a direct cohesin-Chl1 contact, or it could be mediated by another replisome component. To differentiate between these possibilities, we analyzed the interaction of cohesin with Ctf4, which could also be detected during S phase (Figure 7E). The cohesin-Ctf4 interaction was lost in the absence of Chl1, suggesting that the replisome contacts cohesin via Chl1. Taken together, these results are consistent with a scenario in which Chl1 engages with cohesin as the replisome passes cohesin-binding sites, which facilitates retention and acetylation of cohesin during fork passage.

DISCUSSION

In this study, we investigated the contributions of Ctf4 and Chl1 to cohesion establishment and found that Ctf4 recruits Chl1 to the replication fork. Removing Ctf4 from cells that lack Chl1 does not increase their cohesion defect, suggesting that Ctf4's role in sister chromatid cohesion is explained by its role in recruiting Chl1.

Ctf4 forms a homotrimer. Two of the protomers link the replicative helicase via GINS to the DNA polymerase α-primase complex (Simon et al., 2014). This left open whether the third protomer binds a second copy of polymerase α-primase, in analogy to stoichiometries observed at a prokaryotic replication fork (Reyes-Lamothe et al., 2010), or recruits additional unknown factor(s) to the replisome. We found that the latter is the case; Ctf4 recruits Chl1 via a "CIP box" that is shared between the three Ctf4 ligands. Despite these similarities, their respective interactions display individual characteristics. GINS appears to interact with Ctf4 most stably, retaining demonstrable contact throughout the cell cycle. Interactions with polymerase α and Chl1 are detectable only during S phase. Furthermore, Chl1 loses its interaction with Ctf4 earlier than polymerase α. The basis for this distinct temporal regulation remains to be explored. Additional contacts with replisome components might fine-tune the interaction dynamics. The parallel discovery of further CIP box clients that interact with Ctf4 during S phase (Villa et al., 2016) opens intriguing questions as to how their access to Ctf4 is regulated and how handover between CIP box proteins is coordinated.

Ctf4 has also been implicated in other chromosomal functions, including DNA repair and recovery from replication stress (Mimura et al., 2010; Poli et al., 2012; Tsutsui et al., 2005). Reported interactions of vertebrate Ctf4 with replication checkpoint proteins and the Dna2 helicase might contribute to these functions (Errico et al., 2009; Tanaka et al., 2009b; Wawrousek et al., 2010). Fission yeast Ctf4 in turn promotes centromere integrity by associating with the F-box protein Pof3 (Mamnun et al., 2006). One Ctf4 interactor, Mms22, is known to interact with the N-terminal half of Ctf4 (Gambus et al., 2009; Mimura et al.,

(B) The Smc3 acetylation defect of *chl1Δ* cells is corrected by Scc1 overexpression. Cells of the indicated genotypes were synchronized in G1 and released into nocodazole-imposed mitotic arrest. The Smc3 acetylation status was analyzed by immunoblotting using an acetyl-Smc3-specific antibody. Tubulin served as loading control. FACS analysis of DNA content was used to monitor cell-cycle progression.

(C) Chl1 interacts with cohesin at the replication fork. Pk epitope-tagged Scc1 was immunoprecipitated, and co-precipitation of Chl1 was analyzed by immunoblotting. Whole-cell extracts and immunoprecipitates of strains of the indicated genotype were prepared from cultures synchronized in early S phase (HU arrest) or mitosis (nocodazole arrest).

See also Figure S7A for a control that the Chl1-cohesin interaction is CIP box dependent and unaltered by benzonase treatment.

(D) Chl1 interacts with cohesin during S phase. As in (C), but Scc1 was immunoprecipitated from extracts made from aliquots of a culture synchronized by α factor block and release.

(E) Cohesin interaction with the replisome is mediated by Chl1. As in (D), but interaction of Scc1 with Ctf4 was analyzed in strains with or without Chl1.

See also Figure S7B for a ChIP experiment showing that Chl1 is enriched in replicating regions, but not cohesin-binding sites, and Figure S7C, which shows that Chl1 localizes to the replisome independently of cohesion.

2010). This part of the protein is predicted to form a WD40 repeat interaction surface, in addition to the C-terminal WD40 repeats, whose helical extensions form the CIP box acceptor. Ctf4 thus appears to be a multifaceted interaction platform that offers more than one possibility to add functionalities to the replisome.

Ctf4's interaction with its various CIP box clients is reminiscent of PCNA, the DNA sliding clamp that recruits numerous targets to the replication fork via their shared PIP box motif (Georgescu et al., 2015). In addition to its essential role in supporting lagging-strand DNA synthesis by polymerase δ, PCNA engages with proteins involved in Okazaki fragment maturation, base excision repair, and chromatin assembly, among others. While Ctf4 takes on a central position within the replisome, PCNA is thought to remain DNA bound for a certain period following completion of DNA synthesis. In this way, protein interaction platforms exist at two locations, one close to the point of DNA unwinding and one in the wake of the fork. When it comes to sister chromatid cohesion establishment, both of these platforms appear to be used. Chl1 is recruited by Ctf4 to fulfill its function, while Eco1 contains an essential PIP box motif (Moldovan et al., 2006), whose contribution to cohesion establishment remains to be fully understood.

Chl1 is a member of the XPD family of helicases with varied functions in genome stability. Its founding member XPD unwinds DNA during nucleotide excision repair. The family also includes FANCJ and RTEL, with roles in interstrand crosslink repair and telomere maintenance (White, 2009). The Chl1 helicase acts in the recovery from replication stress (Calì et al., 2016; Laha et al., 2011), a function that we find is separable from its role in sister chromatid cohesion. Instead, we describe a physical interaction of Chl1 with the cohesin complex at the replication fork. This finding resolves the conundrum of how Ctf4 and Chl1 facilitate cohesin acetylation, yet act in a genetic pathway in parallel to Eco1 (Borges et al., 2013). Chl1 appears to act at the substrate level, e.g., by orienting the cohesin complex in a way that facilitates Smc3 acetylation. The fact that Ctf4 and Chl1 contribute to sister chromatid cohesion even in the absence of Eco1 suggests that the cohesin-Chl1 interaction facilitates cohesion establishment also independently of acetylation, e.g., by preventing cohesin loss from DNA during fork passage, a possibility that merits further investigation.

Our description of two separable roles of the Chl1 helicase might well be applicable to human ChlR1, which has been implicated both in replication fork restart and sister chromatid cohesion (Calì et al., 2016; Farina et al., 2008). It will be interesting to distinguish whether loss of Chl1 helicase function, or loss of a structural role as cohesin interactor, is the cause of Warsaw breakage syndrome. The observation that the syndrome combines characteristics of DNA repair defects and cohesin deficiency could be explained if both functions contribute.

EXPERIMENTAL PROCEDURES

Yeast culture and the yeast molecular biology techniques, including chromatin immunoprecipitation and replication fork speed measurements, followed standard or otherwise published procedures. These are detailed in the Supplemental Experimental Procedures. For biochemistry and EM analyses, Ctf4 (residues 471–927) and the GINS complex were purified following overexpression in *E. coli*, while Chl1 was purified following overexpression in insect cells. Details of the purification protocols and of the EM and single-particle analysis can be found in the Supplemental Experimental Procedures. An explanation of the structural model of Chl1 and the design of mini-Chl1 is also included there.

SUPPLEMENTAL INFORMATION

Supplemental Information includes Supplemental Experimental Procedures, seven figures, and one table and can be found with this article online at http://dx.doi.org/10.1016/j.molcel.2016.05.036.

AUTHOR CONTRIBUTIONS

C.P.S and F.U. conceived the study, C.P.S. performed most experiments, J.S. and A.L. performed the replication fork speed analyses, P.G. and A.C. reconstituted the recombinant GINS-Ctf4-Chl1 assembly and performed its EM structural analysis, B.O.W. and M.R.S. purified Chl1 and performed ATPase assays, P.A.B. made the Chl1 structural model to design mini-Chl1, and C.P.S. and F.U. wrote the manuscript with input from all authors.

ACKNOWLEDGMENTS

We would like to thank Y. Kakui for help with the microarray analysis, R. Carzaniga and L. Collinson (Crick EM STP) for support on the electron microscope, the Montpellier DNA combing facility (BioCampus), C. Bouchoux for the *MET3pr-eco1-aid* strain, K. Labib for antibodies and communicating unpublished results, and T. Toda and members of our laboratory for discussion and critical reading of the manuscript. This work was supported by the European Research Council and the Francis Crick Institute.

Received: January 18, 2016
Revised: April 24, 2016
Accepted: May 26, 2016
Published: July 7, 2016

REFERENCES

Borges, V., Smith, D.J., Whitehouse, I., and Uhlmann, F. (2013). An Eco1-independent sister chromatid cohesion establishment pathway in *S. cerevisiae*. Chromosoma *122*, 121–134.

Calì, F., Bharti, S.K., Di Perna, R., Brosh, R.M., Jr., and Pisani, F.M. (2016). Tim/Timeless, a member of the replication fork protection complex, operates with the Warsaw breakage syndrome DNA helicase DDX11 in the same fork recovery pathway. Nucleic Acids Res. *44*, 705–717.

Chan, K.-L., Roig, M.B., Hu, B., Beckouët, F., Metson, J., and Nasmyth, K. (2012). Cohesin's DNA exit gate is distinct from its entrance gate and is regulated by acetylation. Cell *150*, 961–974.

Errico, A., Cosentino, C., Rivera, T., Losada, A., Schwob, E., Hunt, T., and Costanzo, V. (2009). Tipin/Tim1/And1 protein complex promotes Pol alpha chromatin binding and sister chromatid cohesion. EMBO J. *28*, 3681–3692.

Farina, A., Shin, J.-H., Kim, D.-H., Bermudez, V.P., Kelman, Z., Seo, Y.-S., and Hurwitz, J. (2008). Studies with the human cohesion establishment factor, ChlR1. Association of ChlR1 with Ctf18-RFC and Fen1. J. Biol. Chem. *283*, 20925–20936.

Gambus, A., Jones, R.C., Sanchez-Diaz, A., Kanemaki, M., van Deursen, F., Edmondson, R.D., and Labib, K. (2006). GINS maintains association of Cdc45 with MCM in replisome progression complexes at eukaryotic DNA replication forks. Nat. Cell Biol. *8*, 358–366.

Gambus, A., van Deursen, F., Polychronopoulos, D., Foltman, M., Jones, R.C., Edmondson, R.D., Calzada, A., and Labib, K. (2009). A key role for Ctf4 in coupling the MCM2-7 helicase to DNA polymerase alpha within the eukaryotic replisome. EMBO J. *28*, 2992–3004.

Georgescu, R., Langston, L., and O'Donnell, M. (2015). A proposal: evolution of PCNA's role as a marker of newly replicated DNA. DNA Repair (Amst.) *29*, 4–15.

Gerlich, D., Koch, B., Dupeux, F., Peters, J.-M., and Ellenberg, J. (2006). Live-cell imaging reveals a stable cohesin-chromatin interaction after but not before DNA replication. Curr. Biol. *16*, 1571–1578.

Gerring, S.L., Spencer, F., and Hieter, P. (1990). The *CHL 1 (CTF 1)* gene product of *Saccharomyces cerevisiae* is important for chromosome transmission and normal cell cycle progression in G_2/M. EMBO J. *9*, 4347–4358.

Haber, J.E. (1974). Bisexual mating behavior in a diploid of *Saccharomyces cerevisiae*: evidence for genetically controlled non-random chromosome loss during vegetative growth. Genetics *78*, 843–858.

Hanna, J.S., Kroll, E.S., Lundblad, V., and Spencer, F.A. (2001). *Saccharomyces cerevisiae* CTF18 and CTF4 are required for sister chromatid cohesion. Mol. Cell. Biol. *21*, 3144–3158.

Heidinger-Pauli, J.M., Mert, O., Davenport, C., Guacci, V., and Koshland, D. (2010). Systematic reduction of cohesin differentially affects chromosome segregation, condensation, and DNA repair. Curr. Biol. *20*, 957–963.

Hirota, Y., and Lahti, J.M. (2000). Characterization of the enzymatic activity of hChlR1, a novel human DNA helicase. Nucleic Acids Res. *28*, 917–924.

Inoue, A., Li, T., Roby, S.K., Valentine, M.B., Inoue, M., Boyd, K., Kidd, V.J., and Lahti, J.M. (2007). Loss of ChlR1 helicase in mouse causes lethality due to the accumulation of aneuploid cells generated by cohesion defects and placental malformation. Cell Cycle *6*, 1646–1654.

Kouprina, N., Kroll, E., Bannikov, V., Bliskovsky, V., Gizatullin, R., Kirillov, A., Shestopalov, B., Zakharyev, V., Hieter, P., Spencer, F., et al. (1992). *CTF4* (*CHL15*) mutants exhibit defective DNA metabolism in the yeast *Saccharomyces cerevisiae*. Mol. Cell. Biol. *12*, 5736–5747.

L Holloway, S. (2000). *CHL1* is a nuclear protein with an essential ATP binding site that exhibits a size-dependent effect on chromosome segregation. Nucleic Acids Res. *28*, 3056–3064.

Laha, S., Das, S.P., Hajra, S., Sanyal, K., and Sinha, P. (2011). Functional characterization of the *Saccharomyces cerevisiae* protein Chl1 reveals the role of sister chromatid cohesion in the maintenance of spindle length during S-phase arrest. BMC Genet. *12*, 83.

Lengronne, A., McIntyre, J., Katou, Y., Kanoh, Y., Hopfner, K.-P., Shirahige, K., and Uhlmann, F. (2006). Establishment of sister chromatid cohesion at the *S. cerevisiae* replication fork. Mol. Cell *23*, 787–799.

Lopez-Serra, L., Lengronne, A., Borges, V., Kelly, G., and Uhlmann, F. (2013). Budding yeast Wapl controls sister chromatid cohesion maintenance and chromosome condensation. Curr. Biol. *23*, 64–69.

Mamnun, Y.M., Katayama, S., and Toda, T. (2006). Fission yeast Mcl1 interacts with SCF(Pof3) and is required for centromere formation. Biochem. Biophys. Res. Commun. *350*, 125–130.

Mayer, M.L., Gygi, S.P., Aebersold, R., and Hieter, P. (2001). Identification of RFC(Ctf18p, Ctf8p, Dcc1p): an alternative RFC complex required for sister chromatid cohesion in *S. cerevisiae*. Mol. Cell *7*, 959–970.

Mayer, M.L., Pot, I., Chang, M., Xu, H., Aneliunas, V., Kwok, T., Newitt, R., Aebersold, R., Boone, C., Brown, G.W., and Hieter, P. (2004). Identification of protein complexes required for efficient sister chromatid cohesion. Mol. Biol. Cell *15*, 1736–1745.

Miles, J., and Formosa, T. (1992). Evidence that POB1, a *Saccharomyces cerevisiae* protein that binds to DNA polymerase α, acts in DNA metabolism in vivo. Mol. Cell. Biol. *12*, 5724–5735.

Mimura, S., Yamaguchi, T., Ishii, S., Noro, E., Katsura, T., Obuse, C., and Kamura, T. (2010). Cul8/Rtt101 forms a variety of protein complexes that regulate DNA damage response and transcriptional silencing. J. Biol. Chem. *285*, 9858–9867.

Moldovan, G.L., Pfander, B., and Jentsch, S. (2006). PCNA controls establishment of sister chromatid cohesion during S phase. Mol. Cell *23*, 723–732.

Murayama, Y., and Uhlmann, F. (2014). Biochemical reconstitution of topological DNA binding by the cohesin ring. Nature *505*, 367–371.

Murayama, Y., and Uhlmann, F. (2015). DNA entry into and exit out of the cohesin ring by an interlocking gate mechanism. Cell *163*, 1628–1640.

Nasmyth, K., and Haering, C.H. (2009). Cohesin: its roles and mechanisms. Annu. Rev. Genet. *43*, 525–558.

Parish, J.L., Rosa, J., Wang, X., Lahti, J.M., Doxsey, S.J., and Androphy, E.J. (2006). The DNA helicase ChlR1 is required for sister chromatid cohesion in mammalian cells. J. Cell Sci. *119*, 4857–4865.

Poli, J., Tsaponina, O., Crabbé, L., Keszthelyi, A., Pantesco, V., Chabes, A., Lengronne, A., and Pasero, P. (2012). dNTP pools determine fork progression and origin usage under replication stress. EMBO J. *31*, 883–894.

Reyes-Lamothe, R., Sherratt, D.J., and Leake, M.C. (2010). Stoichiometry and architecture of active DNA replication machinery in *Escherichia coli*. Science *328*, 498–501.

Rolef Ben-Shahar, T., Heeger, S., Lehane, C., East, P., Flynn, H., Skehel, M., and Uhlmann, F. (2008). Eco1-dependent cohesin acetylation during establishment of sister chromatid cohesion. Science *321*, 563–566.

Rudra, S., and Skibbens, R.V. (2013). Chl1 DNA helicase regulates Scc2 deposition specifically during DNA-replication in *Saccharomyces cerevisiae*. PLoS ONE *8*, e75435.

Simon, A.C., Zhou, J.C., Perera, R.L., van Deursen, F., Evrin, C., Ivanova, M.E., Kilkenny, M.L., Renault, L., Kjaer, S., Matak-Vinković, D., et al. (2014). A Ctf4 trimer couples the CMG helicase to DNA polymerase α in the eukaryotic replisome. Nature *510*, 293–297.

Skibbens, R.V. (2004). Chl1p, a DNA helicase-like protein in budding yeast, functions in sister-chromatid cohesion. Genetics *166*, 33–42.

Tanaka, H., Katou, Y., Yagura, M., Saitoh, K., Itoh, T., Araki, H., Bando, M., and Shirahige, K. (2009a). Ctf4 coordinates the progression of helicase and DNA polymerase α. Genes Cells *14*, 807–820.

Tanaka, H., Kubota, Y., Tsujimura, T., Kumano, M., Masai, H., and Takisawa, H. (2009b). Replisome progression complex links DNA replication to sister chromatid cohesion in *Xenopus* egg extracts. Genes Cells *14*, 949–963.

Tóth, A., Ciosk, R., Uhlmann, F., Galova, M., Schleiffer, A., and Nasmyth, K. (1999). Yeast cohesin complex requires a conserved protein, Eco1p(Ctf7), to establish cohesion between sister chromatids during DNA replication. Genes Dev. *13*, 320–333.

Tsutsui, Y., Morishita, T., Natsume, T., Yamashita, K., Iwasaki, H., Yamao, F., and Shinagawa, H. (2005). Genetic and physical interactions between *Schizosaccharomyces pombe* Mcl1 and Rad2, Dna2 and DNA polymerase alpha: evidence for a multifunctional role of Mcl1 in DNA replication and repair. Curr. Genet. *48*, 34–43.

Uhlmann, F. (2016). SMC complexes: from DNA to chromosomes. Nat. Rev. Mol. Cell Biol. *17*, 399–412.

Uhlmann, F., Lottspeich, F., and Nasmyth, K. (1999). Sister-chromatid separation at anaphase onset is promoted by cleavage of the cohesin subunit Scc1. Nature *400*, 37–42.

Ünal, E., Heidinger-Pauli, J.M., Kim, W., Guacci, V., Onn, I., Gygi, S.P., and Koshland, D.E. (2008). A molecular determinant for the establishment of sister chromatid cohesion. Science *321*, 566–569.

van der Lelij, P., Chrzanowska, K.H., Godthelp, B.C., Rooimans, M.A., Oostra, A.B., Stumm, M., Zdzienicka, M.Z., Joenje, H., and de Winter, J.P. (2010). Warsaw breakage syndrome, a cohesinopathy associated with mutations in the XPD helicase family member DDX11/ChlR1. Am. J. Hum. Genet. *86*, 262–266.

Villa, F., Simon, A.C., Ortiz Bazan, M.A., Kilkenny, M.L., Wirthensohn, D., Wightman, M., Matak-Vinkovíc, D., Pellegrini, L., and Labib, K. (2016). Ctf4 is a hub in the eukaryotic replisome that links multiple CIP-box proteins to the CMG helicase. Mol. Cell *63*, http://dx.doi.org/10.1016/j.molcel.2016.06.009.

Wawrousek, K.E., Fortini, B.K., Polaczek, P., Chen, L., Liu, Q., Dunphy, W.G., and Campbell, J.L. (2010). Xenopus DNA2 is a helicase/nuclease that is found

in complexes with replication proteins And-1/Ctf4 and Mcm10 and DSB response proteins Nbs1 and ATM. Cell Cycle *9*, 1156–1166.

White, M.F. (2009). Structure, function and evolution of the XPD family of iron-sulfur-containing 5′→3′ DNA helicases. Biochem. Soc. Trans. *37*, 547–551.

Wolski, S.C., Kuper, J., Hänzelmann, P., Truglio, J.J., Croteau, D.L., Van Houten, B., and Kisker, C. (2008). Crystal structure of the FeS cluster-containing nucleotide excision repair helicase XPD. PLoS Biol. *6*, e149.

Xu, H., Boone, C., and Klein, H.L. (2004). Mrc1 is required for sister chromatid cohesion to aid in recombination repair of spontaneous damage. Mol. Cell. Biol. *24*, 7082–7090.

Xu, H., Boone, C., and Brown, G.W. (2007). Genetic dissection of parallel sister-chromatid cohesion pathways. Genetics *176*, 1417–1429.

Yoshizawa-Sugata, N., and Masai, H. (2009). Roles of human AND-1 in chromosome transactions in S phase. J. Biol. Chem. *284*, 20718–20728.

Molecular Cell, Volume *63*

Supplemental Information

Ctf4 Links DNA Replication with Sister Chromatid Cohesion Establishment by Recruiting the Chl1 Helicase to the Replisome

Catarina P. Samora, Julie Saksouk, Panchali Goswami, Ben O. Wade, Martin R. Singleton, Paul A. Bates, Armelle Lengronne, Alessandro Costa, and Frank Uhlmann

Figure S1

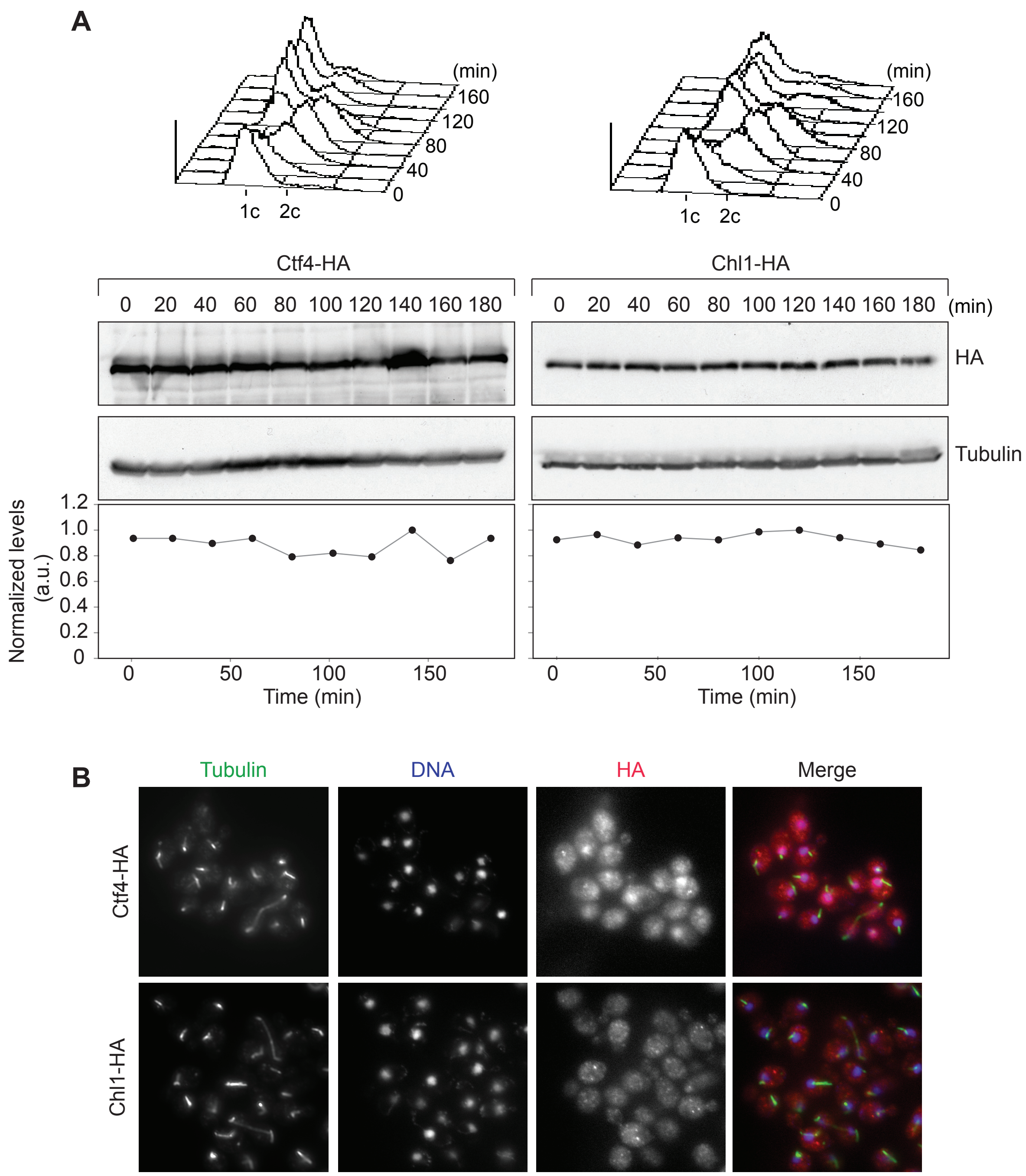

A
(min)
160
120
80
40
0
1c
2c
(min)
160
120
80
40
0
1c
2c
Ctf4-HA
Chl1-HA
0 20 40 60 80 100 120 140 160 180
0 20 40 60 80 100 120 140 160 180 (min)
HA
Tubulin
Normalized levels (a.u.)
1.2
1.0
0.8
0.6
0.4
0.2
0
0 50 100 150
Time (min)
0 50 100 150
Time (min)
B
Tubulin
DNA
HA
Merge
Ctf4-HA
Chl1-HA

Figure S2

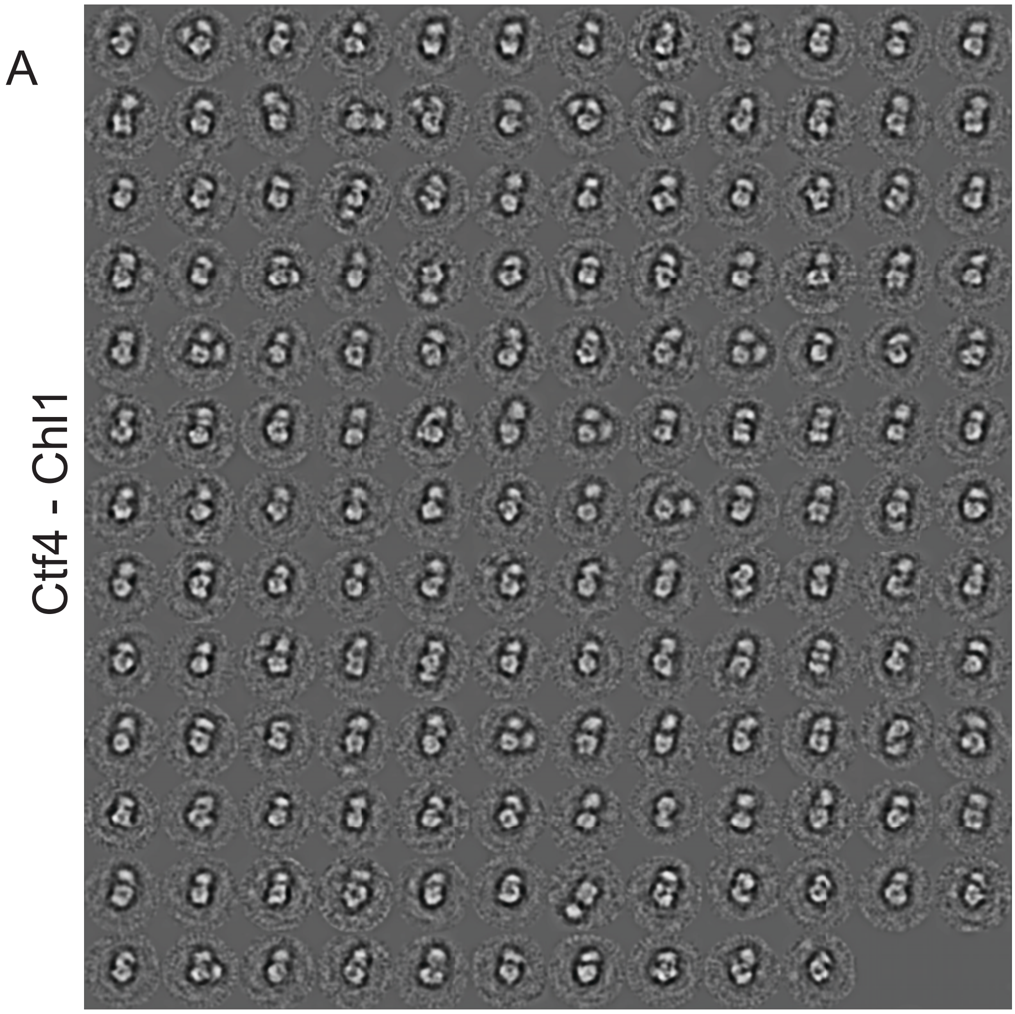

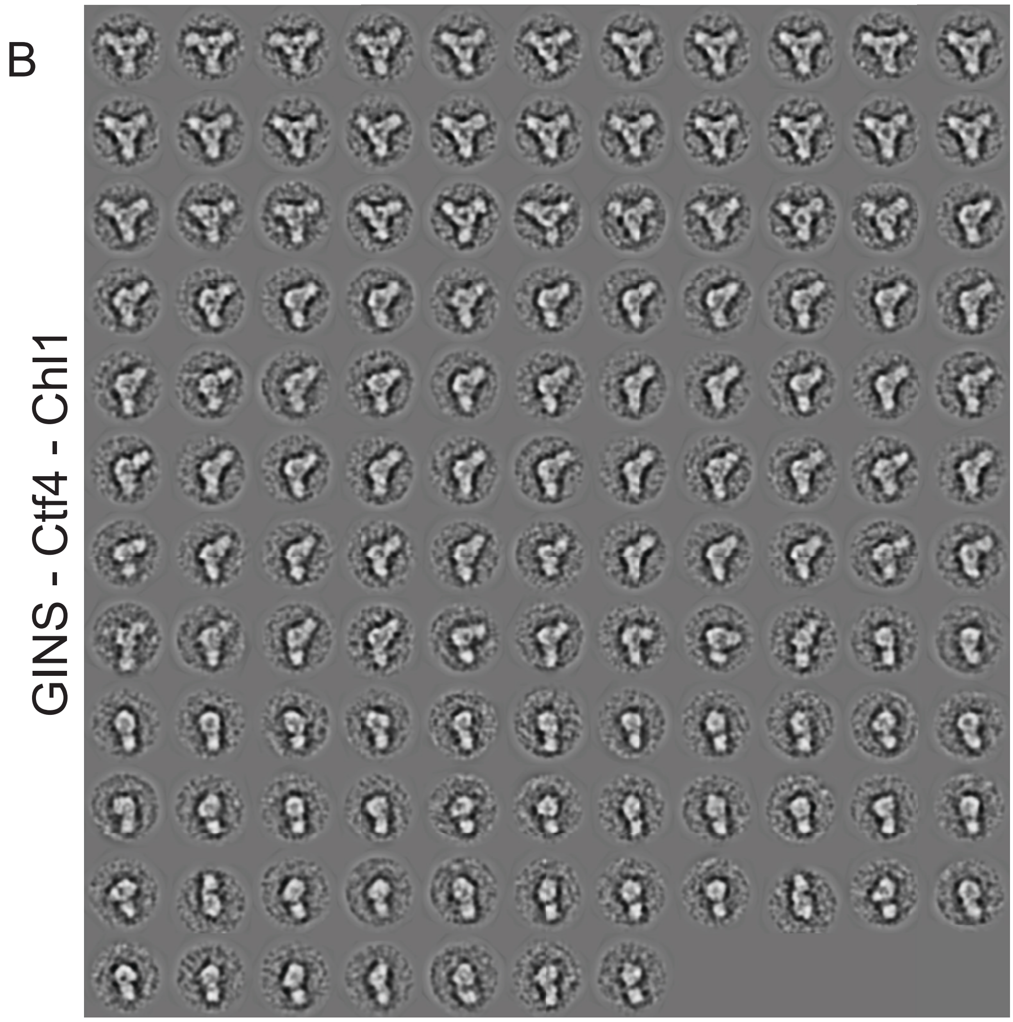

Figure S3

A

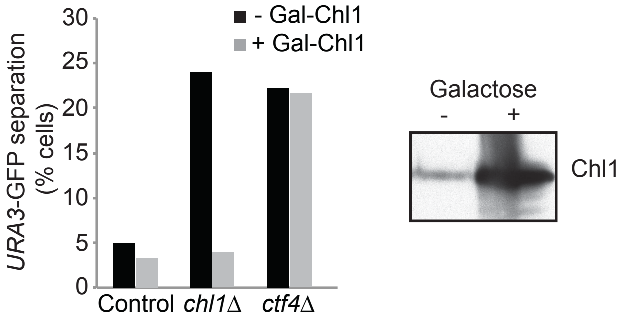

B

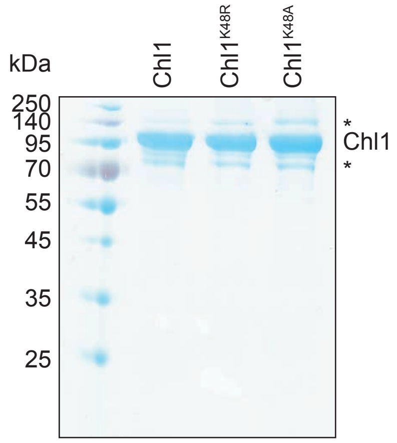

C

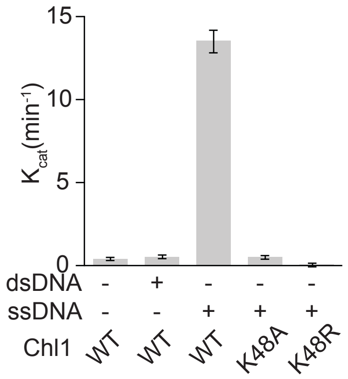

Figure S4

```
Chl1(1-60)          MDKKEYSETFYHPYKPYDIQVQLMETVYRVLSEGKKIAILESPTGTGKTLSLICATMTWL
mini-Chl1(1-52)     MDKKEYSETFYHPYKPYDIQVQLMETVYRVLSEGKKIAILESPTGTGKTLSL--------
XPD(23-40)          -----------------------------------YGVALESPTGSGKTIMAL-------

Chl1(61-120)        RMNKADIFTRMETNIKTNEDDSENLSDDEPDWVIDTYRKSVLQEKVDLLNDYEKHLNEIN
mini-Chl1(53-59)    --KSALQYS---------------------------------------------------
XPD(41-48)          --KSALQYS---------------------------------------------------

Chl1(121-180)       TTSCKQLKTMCDLDKEHGRYKSVDPLRKKRKGARHLDVSLEEQDFIPRPYESDSENNDTS
mini-Chl1           ------------------------------------------------------------
XPD                 ------------------------------------------------------------

Chl1(181-240)       KSTRGGRISDKDYKLSELNSQIITLLDKIDGKVSRDPNNGDRFDVTNQNPVKIYYASRTY
mini-Chl1(60-73)    ----------------------------------------------SERKLKIYYASRTY
XPD(48-61)          ----------------------------------------------SERKLKVLYLVRTN

Chl1(241-300)       SQLGQFTSQLRLPSFPSSFRDKVPDEKVKYLPLASKKQLCINPKVMKWKTLEAINDACAD
mini-Chl1(74-133)   SQLGQFTSQLRLPSFPSSFRDKVPDEKVKYLPLASKKQLCINPKVMKWKTLEAINDACAD
XPD(62-111)         SQEEQVIKELRSLSS---------TMKIRAIPMQGRVNMCILYRMVD-DLHEINAESLAK

Chl1(301-351)       LRHSKEGCIFYQNTNE---WRHCPDTLALR------DMIFSEIQDIEDLVPLGKSLGICP
mini-Chl1(134-184)  LRHSKEGCIFYQNTNE---WRHCPDTLALR------DMIFSEIQDIEDLVPLGKSLGICP
XPD(112-165)        ------FCNMMKKREVMAGNEAACPYFNFKIRSDETKRFLFDELPTAEEFYDYGERNNVCP

Chl1(352-407)       YYASREALPIAEVVTLPYQYLLS----ESTRSSLQINLENSIVIIDEAHNLIETINSIYS
mini-Chl1(185-240)  YYASREALPIAEVVTLPYQYLLS----ESTRSSLQINLENSIVIIDEAHNLIETINSIYS
XPD(166-225)        YESMKAALPDADIVIAPYAYFLNRSVAEKFLSHWGVSRNQIVIILDEAHNLPDIGRSIGS

Chl1(408-466)       SQISLEDLKNCHKGIVTYFN-KFKSRLNPGNRVNLLKLNSLLMTLIQFIVKNFKKIGQEI
mini-Chl1(241-299)  SQISLEDLKNCHKGIVTYFN-KFKSRLNPGNRVNLLKLNSLLMTLIQFIVKNFKKIGQEI
XPD(226-280)        FRISVESLNRADREAQAYGDPELSQKIHVSDLIEMIRS-----ALQSMVSERCGKGDVRI

Chl1(467-526)       DPNDMFTGSNIDTLNIHKLLRYIKVSKIAYKIDTYNQALKEEESSKNENPIKETHKXSVS
mini-Chl1(300-359)  DPNDMFTGSNIDTLNIHKLLRYIKVSKIAYKIDTYNQALKEEESSKNENPIKETHKGSVS
XPD(281-333)        RFQEFMEYMRIMNKRSEREIRSLLN--YLYLFGEYVENEKEKVG-----KVPFSYCSSVA

Chl1(527-586)       SQPLLFKVSQFLYCLTNLTSEGQFFXEKNYSIKYMLLEPSKPFESILNQAKCVVLAGGTM
mini-Chl1(360-419)  SQPLLFKVSQFLYCLTNLTSEGQFFGEKNYSIKYMLLEPSKPFESILNQAKCVVLAGGTM
XPD(334-385)        SRIIAFSDQDEEKYAAILSPE------DGGYMQAACLDPSG-ILEVLKESKTIHMSG-TL

Chl1(587-646)       EPMSEFLSNLLPEVPSEDITTLSCNHVIPKENLQTYITNQPELEFTFEKRMSPSLVNNHL
mini-Chl1(420-479)  EPMSEFLSNLLPEVPSEDITTLSCNHVIPKENLQTYITNQPELEFTFEKRMSPSLVNNHL
XPD(386-436)        DPFDFYSDITGFEIPFKKIG-----EIFPPE--NRYIAYYDGVSSKYDTLDEKELDR--M

Chl1(647-706)       FQFXVDLSKAVPKKGGIVAFXPSYQYLAHVIQCWKQNDRFATLNNVRKIFYEAKDGDDIL
mini-Chl1(480-539)  FQFGVDLSKAVPKKGGIVAFGPSYQYLAHVIQCWKQNDRFATLNNVRKIFYEAKDGDDIL
XPD(437-484)        ATVIEDIILKVKKN--TIVYFPSYSLMDRVENRVS----FEHMKEYRGIDQKE------L

Chl1(707-766)       SGYSDSVAEGRGSLLLAIVGGKLSEGINFQDDLCRAVVMVGLPFXNIFSGELIVKRKHLA
mini-Chl1(540-599)  SGYSDSVAEGRGSLLLAIVGGKLSEGINFQDDLCRAVVMVGLPFGNIFSGELIVKRKHLA
XPD(485-538)        YSMLKKFRRDHG-TIFAVSGGRLSEGN-----ELEMIILAGLPFP-----RPDAINRSLF

Chl1(767-826)       AKIMKSGGTEEEASRATKEFMENICMKAVNQSVGRAIRHANDYANIYLLDVRYNRPNFRK
mini-Chl1(600-659)  AKIMKSGGTEEEASRATKEFMENICMKAVNQSVGRAIRHANDYANIYLLDVRYNRPNFRK
XPD(539-590)        DYYERKYGKGWEYSVVYPTAIK------IRQEIGRLIRSAEDTGACVILDKRAG--QFRK

Chl1(827-861)       KLSRWVQDSINSEHTTHQVISSTRKFFXMRSLNSR
mini-Chl1(660-694)  KLSRWVQDSINSEHTTHQVISSTRKFFGMRSLNSR
XPD(591-615)        FIP--------DMKKTSDPASDIYNFFISAQAR--
```

Figure S5

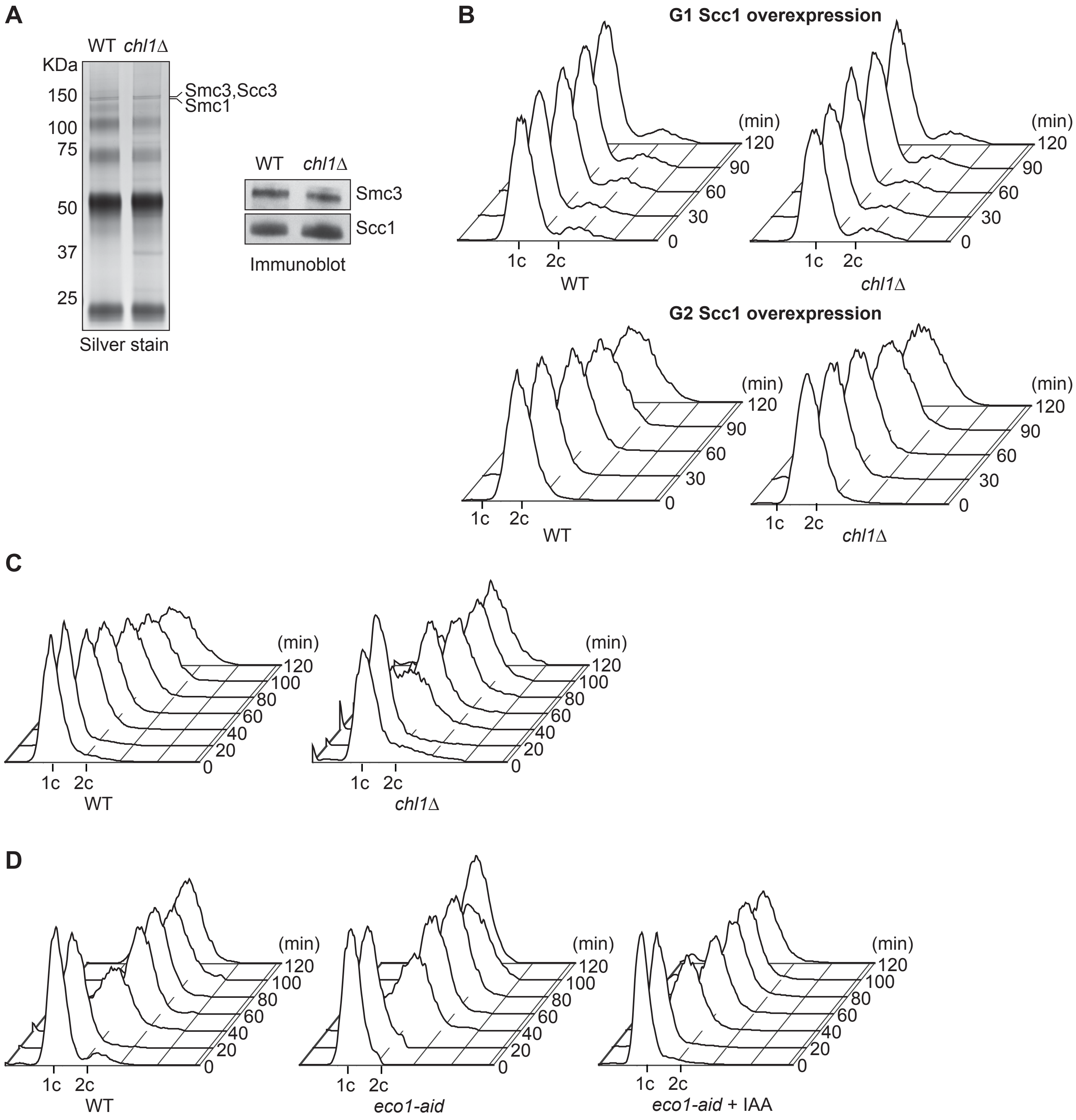
A
WT chl1Δ
KDa
150
100
75
50
37
25
Smc3,Scc3
Smc1
Silver stain
WT chl1Δ
Smc3
Scc1
Immunoblot
B
G1 Scc1 overexpression
(min)
120
90
60
30
0
1c 2c
WT
chl1Δ
G2 Scc1 overexpression
C
(min)
120
100
80
60
40
20
0
1c 2c
WT
chl1Δ
D
WT
eco1-aid
eco1-aid + IAA

Figure S6

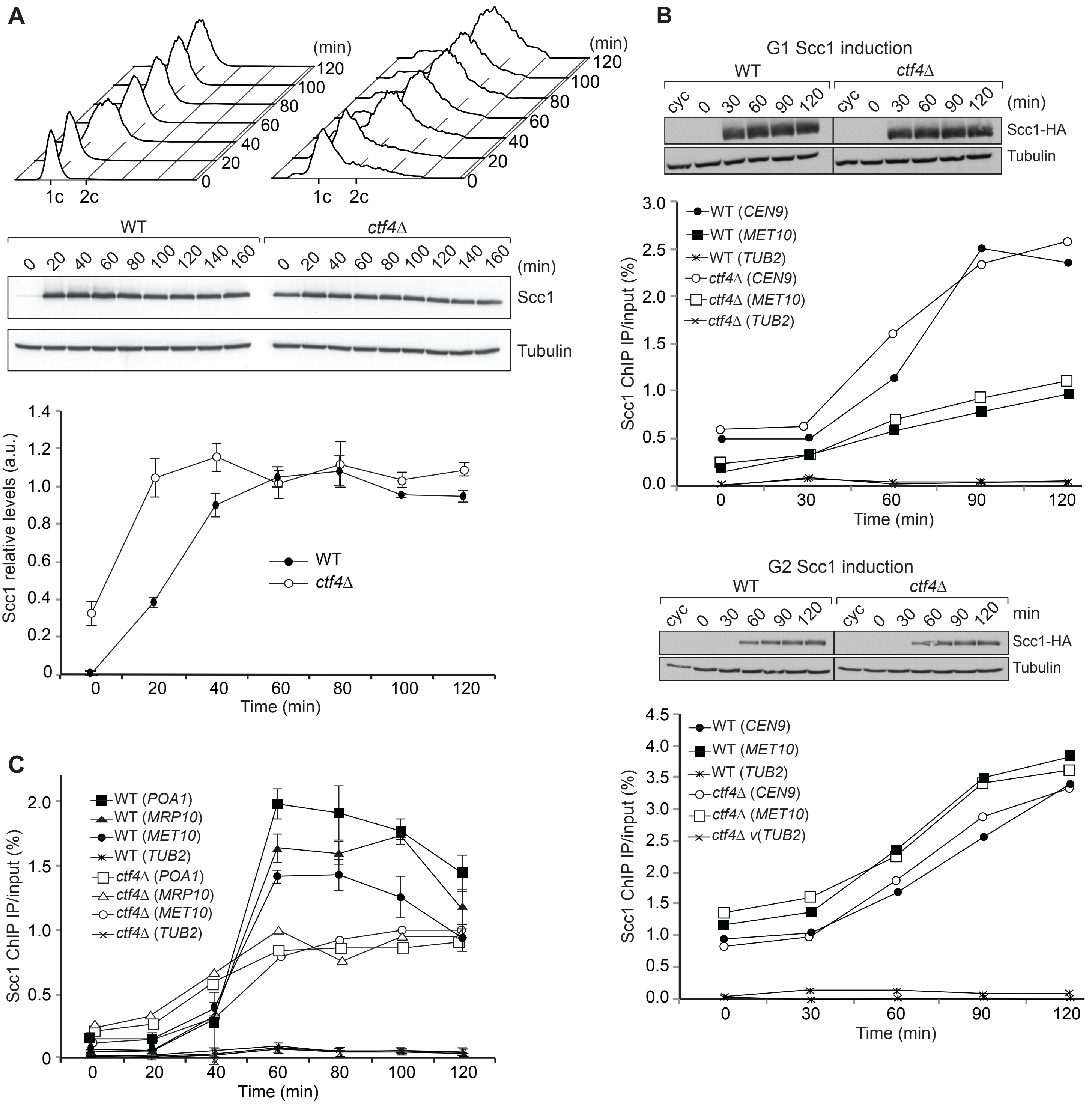
A
(min)
120
100
80
60
40
20
0
1c
2c
WT
ctf4Δ
0 20 40 60 80 100 120 140 160 (min)
Scc1
Tubulin
Scc1 relative levels (a.u.)
Time (min)
WT
ctf4Δ
B
G1 Scc1 induction
WT
ctf4Δ
cyc 0 30 60 90 120 (min)
Scc1-HA
Tubulin
WT (CEN9)
WT (MET10)
WT (TUB2)
ctf4Δ (CEN9)
ctf4Δ (MET10)
ctf4Δ (TUB2)
Scc1 ChIP IP/input (%)
Time (min)
G2 Scc1 induction
WT
ctf4Δ
cyc 0 30 60 90 120 min
Scc1-HA
Tubulin
WT (CEN9)
WT (MET10)
WT (TUB2)
ctf4Δ (CEN9)
ctf4Δ (MET10)
ctf4Δ v(TUB2)
Scc1 ChIP IP/input (%)
Time (min)
C
WT (POA1)
WT (MRP10)
WT (MET10)
WT (TUB2)
ctf4Δ (POA1)
ctf4Δ (MRP10)
ctf4Δ (MET10)
ctf4Δ (TUB2)
Scc1 ChIP IP/input (%)
Time (min)

Figure S7

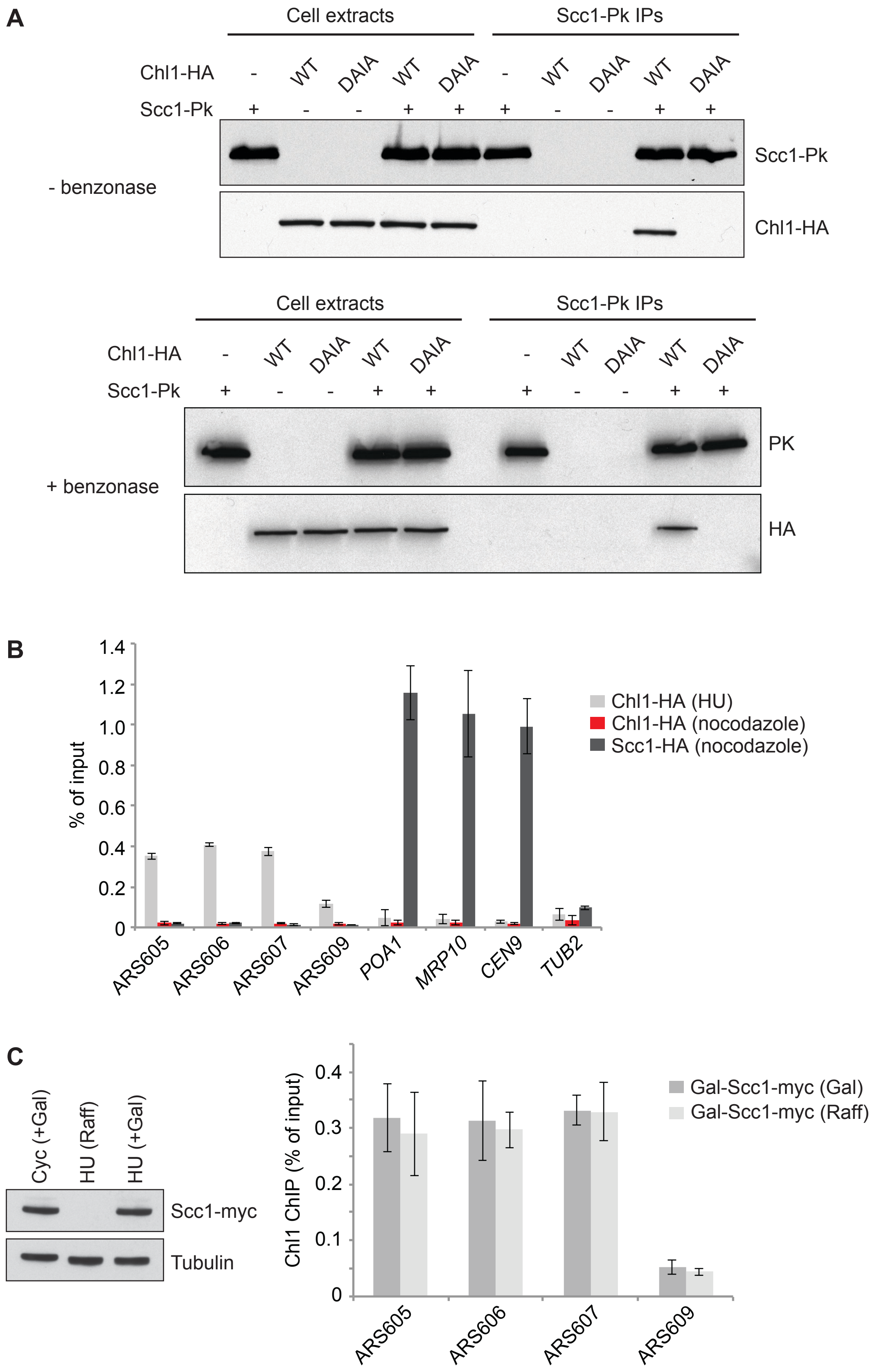
A
Cell extracts
Scc1-Pk IPs
Chl1-HA - WT DAIA WT DAIA - WT DAIA WT DAIA
Scc1-Pk + - - + + + - - + +
- benzonase
Scc1-Pk
Chl1-HA
+ benzonase
PK
HA
B
% of input
Chl1-HA (HU)
Chl1-HA (nocodazole)
Scc1-HA (nocodazole)
ARS605
ARS606
ARS607
ARS609
POA1
MRP10
CEN9
TUB2
C
Cyc (+Gal)
HU (Raff)
HU (+Gal)
Scc1-myc
Tubulin
Chl1 ChIP (% of input)
Gal-Scc1-myc (Gal)
Gal-Scc1-myc (Raff)

SUPPLEMENTAL FIGURE LEGENDS

Figure S1. Constant Ctf4 and Chl1 Levels and Localization throughout the Cell Cycle, Related to Figure 1

(A) Ctf4 and Chl1 levels remain constant throughout the cell cycle. Proteins were detected by quantitative immunobloting against a C-terminal HA epitope tag in extracts from cells blocked in G1 by α-factor treatment and released to progress through a synchronous cell cycle. Cell cycle progression was monitored by FACS analysis of DNA content, tubulin served as a loading control.

(B) Unchanged localization of Ctf4 and Chl1 during the cell cycle. Asynchronously growing cells were fixed and stained for the HA epitope tag on Ctf4 and Chl1, respectively. DNA was stained using DAPI, tubulin staining was used to assign cell cycle stages. Fields with cells at different stages of the cell cycle are shown.

Figure S2: Single-Particle Electron Microscopy Analysis, Related to Figure 3

(A) Two dimensional reference free class averages of the reconstituted Chl1-Ctf4 complex are shown.

(B) as (A), class averages of the Chl1-Ctf4-GINS assembly are shown. Box size is 441 Å x 441 Å.

Figure S3. Chl1 Overexpression Cannot Rescue Sister Chromatid Cohesion in the Absence of Ctf4 and Biochemical Characterization of ATPase-Deficient Chl1, Related to Figure 4

(A) Chl1 depends on Ctf4 for its function in sister chromatid cohesion. Cells of the indicated genotype were synchronized in G1, released, and arrested in metaphase by nocodazole treatment. Chl1 was overexpressed from the *GAL1* promoter for 12 hours prior to the experiment (+Gal-Chl1) or was not expressed (-Gal-Chl1). Sister chromatid cohesion at the GFP-marked *URA3* locus was analyzed. Overexpression was verified by immunoblotting against the HA epitope tag on Chl1. Tubulin served as a loading control.

(B) Purification of recombinant wild type Chl1, Chl1^{K48R} and Chl1^{K48A}. Aliquots of the purified proteins were analyzed by SDS-PAGE followed by Coomassie Blue staining. Two contaminants are marked by asterisks.

(C) Chl1^{K48R} and Chl1^{K48A} do not retain detectable ssDNA-dependent ATPase activity. A continuous ATP-NADH coupled spectrophotometric assay was performed, as described in the Experimental Procedures, to assess ssDNA-dependent ATP hydrolysis by the three Chl1 proteins.

Figure S4. Construction of mini-Chl1, Related to Figure 5

Alignment of the mini-Chl1 protein construct with the Chl1 sequence and the sequence of the structural template used for modelling, *T. acidophium* XPD. Highlighted are the ATPase motifs (red), the Chl1-specific insertion (purple), the 4 cysteines that coordinate the iron sulfur cluster (yellow) and the CIP box (blue).

Figure S5. Cohesin Complex Integrity Is Unaffected in *chl1Δ* Cells and Confirmation of Cell Synchrony, Related to Figure 6

(A) Pk epitope-tagged Scc1 was immunoprecipitated from extracts of the indicated strains, synchronized in early S-phase by HU treatment. Immunoprecipitates were analyzed by silver staining (left panel) or immunoblotting against the HA epitope tag of Smc3 (right panel).

(B - D) FACS analysis of DNA content of the cultures used for the experiments shown in Figures 6B, C and D, respectively.

Figure S6. Ctf4, like Chl1, Is Required for Chromosomal Cohesin Accumulation during S-phase, Related to Figure 6

(A) Scc1 levels are not reduced in cells lacking Ctf4. The experiment was conducted as in Figure 6A, but wild type and *ctf4Δ* cells were compared.

(B) Ectopic cohesin loading onto chromosomes is independent of Ctf4. The experiment was conducted as in Figure 6B, but wild type and *ctf4Δ* cells were compared. All immunoblot samples were run on the

same gel, separated by additional lanes where indicated.

(C) Ctf4 augments cohesin association with chromosomes during S-phase. The experiment was conducted as in Figure 6C, but wild type and *ctf4Δ* cells were compared.

Figure S7. Chl1 Interacts with the Cohesin Complex at the Replication Fork, Related to Figure 7

(A) The cohesin-Chl1 interaction is CIP Box-dependent and benzonase-insensitive. Pk epitope-tagged Scc1 was immunoprecipitated and coprecipitation of Chl1 was analyzed by immunoblotting. Whole cell extracts and immunoprecipitates of strains of the indicated genotype were prepared from cultures arrested in early S-phase by HU treatment as described in Figure 7C. Benzonaze was omitted (top panel) or added (bottom panel) to the extracts prior to immunoprecipitation.

(B) Chl1 is enriched in replicating regions during S phase, but not cohesin binding sites. ChIP was performed against Chl1 or cohesin in cells synchronized in early S-phase (HU) or mitosis (nocodazole). Enrichment close to replication origins (ARS605, 606 and 607) and known cohesin binding sites (*POA1*, *MRP10* and *CEN9*) was analyzed by quantitative real time PCR. ARS609 (an inactive origin in HU) and *TUB2* (a gene where cohesin is not enriched) served as negative controls.

(C) Chl1 binds to replication forks independently of cohesin. Cells in which Scc1 expression is under control of the *GAL1* promoter were grown in medium containing raffinose and galactose. Half of the culture was filtered and shifted to medium containing raffinose only at the time when α-factor was added to synchronize cells in G1. Cells were then released into HU containing medium and aliquots processed for Western blotting to confirm the presence or absence of Scc1 and for ChIP against Chl1. Enrichment at three active and an inactive origin was assessed.

Table S1. Yeast strains used in this study (all Strains were of the W303 background)

Figure 1

(A) Y3615 *MAT***a** *CHL1-Pk*$_{3}$*::TRP1 CTF4-HA*$_{6}$*::HIS3*

(B, C) Y959 *MAT***a** *ura3::URA3/GPD-TK(7x)*

Y3188 *MAT***a** *CTF4-Pk*$_{9}$*::TRP1*

Y4642 *MAT***a** *HA*$_{6}$*-CHL1 (N-terminal tag)*

Figure 2

(B) Y3188 as above

Y4916 *MAT***a** *CHL1-HA*$_{3}$*::LEU2*

Y4694 *MAT***a** *chl1*^{DAIA}*-HA*$_{3}$*::LEU2*

Y4917 *MAT***a** *chl1*^{K48R}*-HA*$_{3}$*::LEU2*

Y4693 *MAT***a** *CTF4-Pk*$_{9}$*::TRP1 CHL1-HA*$_{3}$*::URA3*

Y4826 *MAT***a** *CTF4-Pk*$_{9}$*::HIS3 chl1*^{DAIA}*-HA*$_{3}$*::LEU2*

Y4918 *MAT***a** *CTF4-Pk*$_{9}$*::HIS3 chl1*^{K48R}*-HA*$_{3}$*::LEU2*

(C) Y3188 as above

Y4390 *MAT***a** *CTF4-Pk*$_{9}$*::TRP1 chl1Δ::LEU2*

(D) Y4916 as above

Y3617 *MATa CHL1-HA*$_{3}$*::HIS3 ctf4Δ::TRP*1

Y4694 as above

Y4917 as above

Figure 3

(A) Y4693 as above

Figure 4

(A) K7100 *MAT***a** *ura3::3xURA3::tetO112 his3::HIS3::tetR-GFP*

Y4161 *MAT***a** *ura3::3xURA3::tetO112 his3::HIS3::tetR-GFP ctf4Δ::TRP1*

Y3856 *MAT***a** *ura3::3xURA3::tetO112 his3::HIS3::tetR-GFP chl1Δ::TRP1*

Y4691 *MAT***a** *ura3::3xURA3::tetO112 his3::HIS3::tetR-GFP chl1*^{DAIA}*-HA*$_{3}$*::LEU2*

Y4919 *MAT***a** *ura3::3xURA3::tetO112 his3::HIS3::tetR-GFP chl1*^{K48R}*-HA*$_{3}$*::LEU2*

Y4920 *MAT***a** *ura3::3xURA3::tetO112, his3::HIS3::tetR-GFP chl1*^{K48A}*-HA*$_{3}$*::LEU*2

(B) A2085 *MAT***a** *ura3::URA3/GPD-TK(5x) AUR1c::ADH-hENT1*

A2252 *MAT***a** *ura3::URA3/GPD-TK(5x) AUR1c::ADH-hENT1 ctf4Δ::HIS3*

A2253 *MAT***a** *ura3::URA3/GPD-TK(5x) AUR1c::ADH-hENT1 chl1Δ::HIS3*

(C) A872 *ura3::URA3/GPD-TK(7x) RAD5*

A908 *ura3::URA3/GPD-TK(7x) RAD5 ctf4Δ::HIS3*

A911 *ura3::URA3/GPD-TK(7x) RAD5 chl1Δ::HIS3*

(D) Y141 W303 wild type

Y4323 *MAT***a** *ctf4Δ::TRP1*

Y4324 *MAT***a** *chl1Δ::TRP1*

Y4694 as above

Y4917 as above

Figure 5

(B) Y4916 as above

Y4921 *MAT***a** *miniCHL1-*HA_3*::LEU2*

(C) K7100 as above

Y3856 as above

Y4922 *MAT***a** *ura3::3xURA3::tetO112 his3::HIS3::tetR-GFP miniCHL1-*HA_3*::LEU2*

(D) Y141 as above

Y4323 as above

Y4324 as above

Y4921 as above

Figure 6

(A) Y2269 *MAT***a** *SCC1-*Pk_9*::TRP1*

Y4389 *MAT***a** *SCC1-*Pk_9*::TRP1 chl1Δ::LEU2*

(B) Y4380 *MAT***a** *ura3::3xURA3::tetO112 his3::HIS3::tetR-GFP GAL-SCC1-*HA_3*::LEU2*

Y4386 *MAT***a** *ura3::3xURA3::tetO112 his3::HIS3::tetR-GFP GAL-SCC1-*HA_3*::LEU2 chl1Δ::TRP1*

Y4923 *MAT***a** *GAL-SCC1(R180D,R268D)-*HA_3*::LEU2*

Y4924 *MAT***a** *GAL-SCC1(R180D,R268D)-*HA_3*::LEU2 chl1Δ::HIS3*

(C) Y2269 as above

Y4389 as above

(D) Y4766 *MAT***a** *pADH1-OsTir1-*myc_9*::ADE2 SCC1-6E2::TRP1 HIS3::pMET3-eco1-IAA17::*KAN^R

Y4925 *MAT***a** *SCC1-6E2::TRP1*

Figure 7

(A) Y2012 *MAT***a** *scc1-73 ura3::3xURA3::tetO112 his3::HIS3::tetR-GFP GAL-SCC1-*HA_3*::LEU2*

Y4384 *MAT***a** *ura3::3xURA3::tetO112 his3::HIS3::tetR-GFP GAL-SCC1-*HA_3*::LEU2 ctf4Δ::TRP1*

Y4386 as above

Y4383 *MAT***a** *ura3::3xURA3::tetO112 his3::HIS3::tetR-GFP GAL-SCC1-*HA_3*::LEU2 mrc1Δ::TRP1*

Y4385 *MAT***a** *ura3::3xURA3::tetO112 his3::HIS3::tetR-GFP GAL-SCC1-*HA_3*::LEU2 ctf18Δ::TRP1*

Y4382 *MAT***a** *ura3::3xURA3::tetO112 his3::HIS3::tetR-GFP GAL-SCC1-* HA_3*::LEU2 tof1Δ::TRP1*

Y4381 *MAT***a** *ura3::3xURA3::tetO112 his3::HIS3::tetR-GFP GAL-SCC1-*HA_3*::LEU2 csm3Δ::TRP1*

(B) Y141 as above
Y4324 as above
Y4386 as above

(C, D) Y2269 as above
Y4916 as above
Y4926 *MAT***a** *Scc1-*Pk_9*::TRP1 CHL1-*HA_3*::LEU2*
Y4927 *MAT***a** *Scc1-*Pk_9*::TRP1 CHL1-*HA_3*::LEU2 ctf4Δ::HIS3*

(E) Y5040 *MAT***a** *Scc1-*HA_6*::TRP1 CTF4-*Pk_9*::HIS3*
Y4927 *MAT***a** *Scc1-*HA_6*::TRP1 CTF4-*Pk_9*::HIS3 chl1Δ::LEU2*

Figure S1

(A) Y1451 *MAT***a** *CTF4-*HA_6*::HIS3*
Y3613 *MAT***a** *CHL1-*HA_6*::HIS3*

(B) K1451 as above
Y3613 as above

Figure S3

(A) Y4928 *MAT***a** *ura3::3xURA3::tetO112 his3::HIS3::tetR-GFP GAL-CHL1-*HA_3*::LEU2*
Y4929 *MAT***a** *ura3::3xURA3::tetO112 his3::HIS3::tetR-GFP GAL-CHL1-*HA_3*::LEU2 chl1Δ::TRP1*
Y4930 *MAT***a** *ura3::3xURA3::tetO112 his3::HIS3::tetR-GFP GAL-CHL1-*HA_3*::LEU2 ctf4Δ::TRP1*

Figure S5

(A) Y4931 *MAT***a** *SCC1-*Pk_9*::TRP1 SMC3-*HA_6*::HIS3*
Y4932 *MAT***a** *SCC1-*Pk_9*::TRP1 SMC3-*HA_6*::HIS3 chl1Δ::LEU2*

Figure S6

(A) Y4388 *MAT***a** *SCC1-*Pk_9*::TRP1 ctf4Δ::LEU2*

(B) Y4384 as above
Y4933 *MAT***a** *GAL-SCC1(R180D,R268D)-*HA_3*::LEU2 ctf4Δ::HIS3*

(C) Y4388 as above

Figure S7

(A) Y2269 as above
Y4916 as above
Y4694 as above
Y4926 as above

Y4934 *MAT***a** *Scc1-*Pk_9*::TRP1 chl1*^{DAIA}*-*HA_3*::LEU2*

(B) Y4642 as above

Y4220 *MAT***a** *Scc1-*HA_6*::TRP1*

(C) Y5041 *MAT***a** *scc1Δ::URA3 GAL-SCC1*myc_{18} *CHL1-*HA_6*::HIS3*

Supplemental Experimental Procedures

Yeast Strains and Culture

All strains used in this study were derivatives of W303 and are listed in Table S1. Gene deletions and epitope tagging of endogenous genes were performed by gene targeting using polymerase chain reaction (PCR) products (Knop et al., 1999; Wach et al., 1994). Cells were grown in rich YP medium or in complete synthetic medium lacking methionine, supplemented with either 2% glucose or 2% raffinose as the carbon source (Amberg et al., 2005). To induce gene expression from the *GAL1* promoter, 2% galactose was added to cells grown in raffinose-containing medium. Overexpression of Scc1 in G1 was achieved by *GAL1* promoter-driven expression of a non-cleavable variant, Scc1(R180D,R268D) (Uhlmann et al., 1999). Cells were synchronized in G1 by adding α-factor (0.04 mg/ml) for 2 hours. To arrest cells in early S-phase, G1 synchronized cultures were released from α-factor block into medium containing 0.1 M hydroxyurea (HU) for 60 minutes. Arrest in G2/M was achieved by release into medium containing 5 mg/ml nocodazole for 120 minutes. When cells were released to pass through a complete, synchronous cell cycle, α-factor was readded to the culture, once cells started budding, for re-arrest in the following G1. To inactivate Eco1, its gene promoter was replaced with the methionine-repressible *MET3* promoter and its C-terminus fused to an auxin-inducible degron (Nishimura et al., 2009). Cells were grown in medium lacking methionine and shifted to medium containing methionine and 500 μM of the auxin indole-3-acetic acid (IAA) 1 hour before release from α-factor block into synchronous cell cycle progression. Strains expressing $Chl1^{DAIA}$, $Chl1^{K48R}$ or $Chl1^{K48A}$ were engineered by endogenous gene replacement using an integrative targeting plasmid. To generate the mini-Chl1 derivative, amino acids 53-231 in Chl1 were replaced by the amino acid sequence KSALQYSSERKL (compare Figure S4).

Yeast Molecular Biology Techniques

Coimmunoprecipitation assays were performed as described (Godfrey et al., 2015), except that cell extracts were prepared using buffer containing 100 mM Hepes-KOH (pH 7.9), 300 mM potassium acetate, 10 mM magnesium acetate, 10% glycerol, 0.1% NP-40, 2 mM EDTA, 2 mM β-glycerophosphate, 2 mM sodium fluoride, 1 mM dithiothreitol, a protease inhibitor cocktail (Roche and Sigma) and treated with benzonase (Merck Biosciences), as described (Gambus et al., 2009). Antibodies used for immunoprecipitation and Western blotting were anti-Pk (clone SV5-Pk1, Serotec), anti-HA (clone F-7, Santa Cruz), anti-α-tubulin (clone YOL1/34, Serotec), anti-myc (clone 9E10), an antibody specific to acetylated Smc3 (Borges et al., 2010) and anti-Psf2 and anti-Pol1 antisera that were a kind gift from K. Labib. Quantitative analysis of immunoblots was performed with an ImageQuant LAS 4010 imager (GE Healthcare).

Chromatin immunoprecipitation and microarray analysis were performed as previously described (Lengronne et al., 2004), as was the quantitative analysis of chromatin immunoprecipitates (Lopez-Serra et al., 2014). Analysis of sister chromatid cohesion followed a published procedure (Michaelis et al., 1997).

Chl1 expression and purification and its use in ATPase assays

Wild type and mutant Chl1, fused via a TEV protease recognition sequence to a Strep-tag for affinity purification, were expressed from recombinant baculoviruses in High Five insect cells (Life Technologies). Harvested cells were sonicated in buffer containing 50 mM Tris-HCl (pH 8.5), 300 mM NaCl, 10% glycerol, 0.5 mM TCEP and centrifuged at 35000 x g at 4°C for 1 hour. The supernatant was loaded onto a 5 ml Strep-Tactin superflow plus cartridge (Qiagen) and eluted in buffer containing 1 mg/ml of desthiobiotin. The Strep-tag was cleaved off overnight at 4°C using 0.04 mg/ml TEV protease. The protein was then loaded onto a Superdex 200 10/300 GL column (GE Healthcare), equilibrated with 50 mM Tris-HCl (pH 8.5), 300 mM NaCl and 0.5 mM TCEP. Eluted protein-containing fractions were analyzed for purity by SDS polyacrylamide gel electrophoresis. To measure ATPase activity, a continuous ATP-NADH coupled spectrophotometric assay was performed. The change in absorbance at 340 nm was recorded for 10 minutes at 20°C in a reaction consisting of 100 mM Tris-HCl (pH 8.5), 300 mM NaCl, 10 mM $MgCl_2$, 1 mM ATP, 40 U/ml pyruvate kinase, 50 U/ml lactate dehydrogenase, 0.5 mM phosphoenolpyruvate, 0.2 mM NADH, with or without 3.5 μM (molecules) of DNA substrate. A 20mer ssDNA and 21mer dsDNA with sequences oligo d(T) and 5’-

TCTCCACAGGAAACGGAGGGT-3' respectively (Sigma), were used as substrates. Chl1 was added to start the reaction at a concentration of 3.5 μM.

Protein Expression, Purification and Complex Reconstitution for Electron Microscopy

Ctf4 purification: N-terminally His-tagged *S. cerevisiae* Ctf4 (residues 471-927) was expressed in (DE3)-CodonPlus cells (Stratagene) transformed with a pRSFDuet-1-based construct (a kind gift of Luca Pellegrini) (Simon et al., 2014). Six liters of cells were grown in LB medium to an optical density of 0.6 before induction with 1 mM IPTG, and overnight expression at 20°C. The cleared lysate was incubated with 3 ml Ni-NTA resin (Qiagen), washed with 5 column volumes (CV) of buffer A (50 mM NaH_2PO_4 pH 8.0, 300 mM NaCl, 20 mM imidazole) and eluted with 5 ml of buffer A supplemented with 230 mM imidazole. The eluted protein was further purified by gel filtration chromatography over a Superdex 200 16/60 HiLoad column (GE Healthcare) in 25 mM HEPES-NaOH pH 7.0, 200 mM NaCl and 10% glycerol. Pooled fractions were concentrated to 5 mg/ml and stored at -80°C (in 2 nmol aliquots).

GINS purification: Full-length *S. cerevisiae* GINS was cloned into pET28c (Novagen) and carries an N-terminal Strep III tag on the Psf3 subunit (a kind gift of Karim Labib) (Gambus et al., 2009). This expression construct was transformed into BL21 (DE3)-CodonPlus cells. Lysates from 2 liters LB culture was incubated with with 3 ml StrepTactin resin (IBA Life Sciences), washed with five column volumes of buffer B (25 mM HEPES-NaOH pH 7.5, 300 mM NaCl, 10% Glycerol, 0.5 mM TCEP) and eluted with 4 ml buffer B supplemented with 2.5 mM desthiobiotin (IBA Life Sciences). Proteins were stored at -80°C at a concentration of 1 mg/ml (in 2 nmol aliquots).

Chl1 purification: pFastBac1 plasmids containing the budding yeast *CHL1* gene was used to produce a bacmid, which was subsequently transfected into Sf9 cells to generate recombinant baculovirus expressing Chl1, which was amplified over two rounds in Sf9 cells grown in Graces medium supplemented with 10% FCS. The infection for protein purification was carried out by inoculating 6 liters of Hi5 cells at 10^6 cells/ml by adding 50 ml of virus stock per liter of Hi5 cells. Infected cells were incubated for 48 hours at 27°C, harvested by centrifugation and washed with PBS + 5 mM $MgCl_2$. The collected cells were re-suspended in 200 ml of resuspension buffer (25 mM HEPES-NaOH pH 7.6, 0.02% Tween-20, 10% glycerol, 1 mM EDTA, 1 mM EGTA) supplemented with 15 mM KCl, 2 mM $MgCl_2$, 0.4 mM PMSF, 2 mM 2-mercaptoethanol and the complete protease inhibitors cocktail from Roche Diagnostics. The cell suspension was snap frozen in 10 ml aliquots and stored at -80°C. The cell suspension once thawed was broken using a Dounce homogenizer. This was followed by centrifugation at 14,000 rpm in an Avanti J-26S XP centrifuge for 10 minutes to clear the extract. The cleared extract was incubated with 4 ml of StrepTactin resin (IBA Life Sciences) for 2 hours with continuous end-over-end mixing. The beads were then collected in a 20 ml Poly-Prep disposable chromatography column (BioRad) and washed with 30 ml buffer C (50 mM Tris-HCl pH8.5, 300 mM NaCl, 10% Glycerol and 3 mM DTT). Bound complexes were eluted in buffer C with 2.5 mM desthiobiotin and complete protease inhibitor cocktail. Pooled elution fractions were further purified on a Mono Q (GE Healthcare) ion exchange column, pre-equilibrated in Buffer D (10 mM Tris-HCl pH 8.5, 100 mM NaCl and 3 mM DTT). Bound complexes were eluted with 10 ml of a 100-250 mM NaCl linear gradient and collected in 500 μl fractions. Peak fractions were pooled and dialyzed for 16 hours against 25 mM HEPES-NaOH pH 7.6, 150 mM NaCl, 2 mM DTT. Proteins were stored at -80 °C at a concentration of 5 mg/ml (in 2 nmol aliquots).

To reconstitute the Ctf4-client protein complexes, Ctf4, Chl1 and GINS preparations were first dialyzed separately to 25 mM HEPES-NaOH pH 7.6, 500 mM sodium acetate, 0.5 mM DTT for 1 h at 4 °C in dialysis tubes with a 6,000-8,000 Da molecular weight cutoff (GeBAflex). 2 nmol of each component were then coincubated for 10 minutes on ice in a 100 μl volume. The reconstitution mix was initially dialyzed in 400 mM sodium acetate, 25 mM HEPES-NaOH pH 7.6, 0.5 mM DTT for 1 h at 4°C. The dialysis buffer was changed hourly to contain progressively 300 mM, 200 mM and finally 150 mM sodium acetate. The reconstituted complexes were crosslinked with 0.1% glutaraldehyde for 20 minutes followed by deactivation with 80 mM Glycine.

Electron Microscopy and Single Particle Analysis

Carbon was evaporated onto freshly cleaved mica with a Q150TE coater (Quorum Technologies) and incubated overnight before coating 400 mesh grids (Agar Scientific). Dried carbon grids were glow discharged for 30–60 seconds at 45 mA using a 100x glow discharger (Electron Microscopy Sciences). A 4 μl drop of the reconstituted sample was applied onto the grid and incubated for 1 minute. Grids

were then sequentially stirred over five distinct 75 µl drops of 2% uranyl formate solution, 10 seconds per drop. The excess stain was blotted away until the grids were dry.

Image data from the negative stained grids were collected using a Tecnai LaB6 G2 Spirit transmission electron microscope (FEI) operating at 120 keV. A GATAN Ultrascan 100 camera at a nominal magnification of 30,000x (3.45 Å/pixel at the specimen level) was used to record images. For each dataset between 100 and 150 micrographs were collected. Particle picking, contrast transfer function estimation and correction were all performed using EMAN2.1 software. Multivariate statistical analysis-based classification was performed in Imagic, as previously described (Simon et al., 2014).

Replication Fork Speed Measurements

Replication fork progression was quantitatively analyzed following BrdU incorporation and DNA fibre combing, as previously described (Bianco et al., 2012).

Structural model of *S. cerevisiae* Chl1 and design of mini-Chl1

The 3-dimensional model and alignment of Chl1 were based on the crystal structure of *T. acidophilum* XPD (Wolski et al., 2008) and were generated by the program 3D-JIGSAW (Bates et al., 2001). A three-state protein secondary structure prediction for the Chl1-specific insert predicts it to consist of long helical and coiled segments and is therefore likely to be more prone to large conformational changes compared to the remainder of the Chl1 sub-domain structures, which have less coil segments along with stabilizing strands and, therefore, β-sheet architecture. Perhaps as a consequence of this likely higher degree of flexibility, the insert domain currently has no structural homology to an experimentally determined protein structure and could not be modeled with sufficient accuracy. The link used to connect the first region of the helicase, the region containing the N-terminal ATPase motif, GxGKT, is based on native sequence from XPD, which mainly consists of a helical segment, as visual inspection of a number of 3D alternative models indicated this to be the most parsimonious way to connect sub-domains while maintaining their native packing geometry. Alignment of the motifs required for ATPase activity (see above, and DEAH) and the four cysteine residues that coordinate the 4Fe-4S cluster indicates an acceptable alignment between the Chl1 model and the XPD template (achieving approximately 21% sequence identity).

Supplemental References

Amberg, D.C., Burke, D.J., and Strathern, J.N. (2005). Methods in yeast genetics (Cold Spring Harbor, New York: Cold Spring Harbor Laboratory Press).

Bates, P.A., Kelley, L.A., MacCallum, R.M., and Sternberg, M.J. (2001). Enhancement of protein modeling by human intervention in applying the automatic programs 3D-JIGSAW and 3D-PSSM. Proteins *Suppl 5*, 39-46.

Bianco, J.N., Poli, J., Saksouk, J., Bacal, J., Silva, M.J., Yoshida, K., Lin, Y.-L., Tourriere, H., Lengronne, A., and Pasero, P. (2012). Analysis of DNA replication profiles in budding yeast and mammalian cells using DNA combing. Methods *57*, 149-157.

Borges, V., Lehane, C., Lopez-Serra, L., Flynn, H., Skehel, M., Rolef Ben-Shahar, T., and Uhlmann, F. (2010). Hos1 deacetylates Smc3 to close the cohesin acetylation cycle. Mol. Cell *39*, 677-688.

Godfrey, M., Kuilman, T., and Uhlmann, F. (2015). Nur1 dephosphorylation confers positive feedback to mitotic exit phosphatase activation in budding yeast. PLoS Genet. *11*, e1004907.

Knop, M., Siegers, K., Pereira, G., Zachariae, W., Winsor, B., Nasmyth, K., and Schiebel, E. (1999). Epitope tagging of yeast genes using a PCR-based strategy: more tags and improved practical routines. Yeast *15*, 963-972.

Lengronne, A., Katou, Y., Mori, S., Yokobayashi, S., Kelly, G.P., Itoh, T., Watanabe, Y., Shirahige, K., and Uhlmann, F. (2004). Cohesin relocation from sites of chromosomal loading to places of convergent transcription. Nature *430*, 573-578.

Lopez-Serra, L., Kelly, G., Patel, H., Stewart, A., and Uhlmann, F. (2014). The Scc2-Scc4 complex acts in sister chromatid cohesion and transcriptional regulation by maintaining nucleosome-free regions. Nat. Genet. *46*, 1147-1151.

Michaelis, C., Ciosk, R., and Nasmyth, K. (1997). Cohesins: Chromosomal proteins that prevent premature separation of sister chromatids. Cell *91*, 35-45.

Nishimura, K., Fukagawa, T., Takisawa, H., Kakimoto, T., and Kanemaki, M. (2009). An auxin-based degron system for the rapid depletion of proteins in nonplant cells. Nat. Methods *6*, 917-922.

Wach, A., Brachat, A., Pöhlmann, R., and Philippsen, P. (1994). New heterologous modules for classical or PCR-based gene disruptions in *Saccharomyces cerevisiae*. Yeast *10*, 1793-1808.
